# Allenyl Thianthrenium Salt: A Bench-Stable C_3_ Synthon for Annulation and Cross-Coupling Reactions

**DOI:** 10.1021/jacs.4c10135

**Published:** 2024-09-24

**Authors:** Srija Tewari, Nicolai Klask, Tobias Ritter

**Affiliations:** † Max-Planck-Institut für Kohlenforschung, Kaiser-Wilhelm-Platz 1, 45470 Mülheim an der Ruhr, Germany; ‡ Institute of Organic Chemistry, RWTH Aachen University, Landoltweg 1, 52074 Aachen, Germany

## Abstract

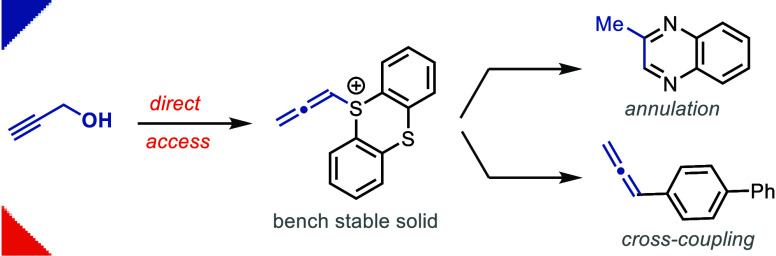

Herein, we report the first bench-stable and nonhygroscopic monosubstituted allenyl sulfonium salt (ATT) synthesized from thianthrene and propargyl alcohol. We demonstrate its use in annulation chemistry to synthesize heterocycles, such as 2-hydroxy morpholine, 2-methyl quinoxalines, and benzodioxepinone derivatives, with an exocyclic double bond. The reagent is the first allenyl sulfonium salt that can undergo palladium-catalyzed cross-coupling reactions to form a C(sp^2^)–C(sp^2^) bond via Suzuki coupling and a C(sp^3^)–C(sp^2^) bond formation via reductive coupling.

Allenes are 1,2-dienes that serve as the backbone for about 150 known natural products^1−5^ and have various industrial applications, such as in dyes, polymers for paints, printable films, etc.^2^ Allenic moieties are also found in pharmacologically active compounds due to their biological activity as part of cytotoxic^6^ or antiviral agents.^7^ Because of their perpendicular π bonds,^2,8^ they also provide potential for diversification via cyclization,^9−13^ enantioselective allylation,^14^ and acylation of amines and alcohols.^15^ However, the synthesis of allenes, especially with allenylating reagents, is challenging. While many propargylating reagents, such as propargyl bromide, are readily available, electrophilic allenylating reagents are not. Typically, allenylation is performed with propargylating reagents through appropriate isomerization (Figure 1A), which often gives product mixtures.^16^ Herein, we report the development of the first bench-stable and nonhygroscopic monosubstituted allenyl sulfonium salt (ATT^**+**^, **1**). The allenyl electrophile is a free-flowing solid that does not exist in dynamic equilibrium with its propargyl isomer. Reagent **1** can be synthesized in one step on a multigram scale and stored in air for at least a year without any signs of decomposition (Figure 1B). It can be used as a versatile C_3_ synthon to synthesize heterocycles, such as benzodioxepinones, quinoxaline derivatives, and dihydrobenzodioxine, via cyclization (Figure 1C). Additionally, reagent **1** can introduce an allenyl functionality directly to aromatics via cross-coupling, which is not currently accessible with propargyl sulfonium salts.

**Figure 1:**
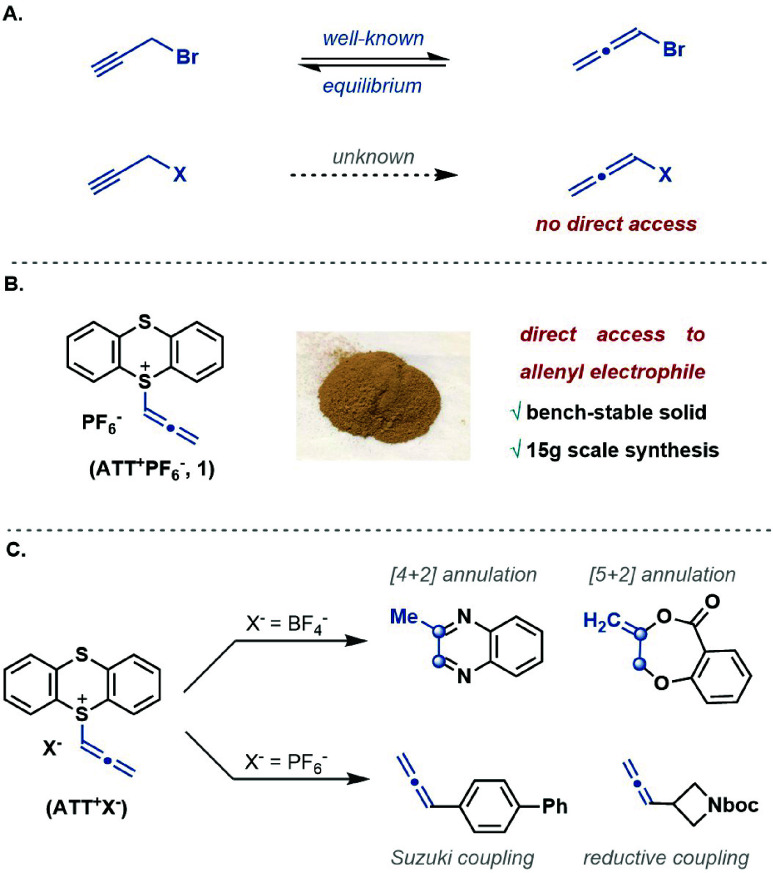
(A) Equilibrium synthesis of allenyl electrophiles, X=^+^SR_2_, (B) Allenyl thianthrenium salt (ATT^+^PF_6_^–^, **1**), and (C) applications of ATT^+^–X^–^.

The Stirling group^15^ reported the first example of a monosubstituted allenyl sulfonium salt (Me_2_S^+^–C_3_H_3_Br^–^), which was formed *in situ* from its propargyl isomer. However, demethylation by the bromide counteranion led to its decomposition. There are recent reports of using tetrahydrothiophenium propargyl bromide for epoxidation^17^ and cyclopropanation.^18^ Propargyl bromide can also undergo isomerization to its allenyl form (resulting in an equilibrium ratio of 3:7 with the latter) upon heating with a catalytic amount of triphenylphosphine.^19^ A series of structurally diverse sulfonium salts ranging from aromatic to aliphatic substituents has been reported.^20−22^ However, a report of a bench-stable allenyl electrophile that can be directly accessed and stored for a prolonged period has, to the best of our knowledge, not been disclosed as of yet.

In this work, we have developed allenyl thianthrenium salt **1**, which is an easy-to-handle solid. The synthesis of reagent **1** proceeds via the formation of propargyl thianthrenium salt (PTT^+^) **1a** upon reaction of thianthrene with propargyl triflate,^22^ formed *in situ* from triflic anhydride and propargyl alcohol in the presence of potassium carbonate as a base (Figure 2). The propargyl thianthrenium triflate **1a** is formed in a 3:1 ratio with allenyl thianthrenium triflate **1-OTf**, possibly by isomerization from **1a**. We found empirically that conversion of propargyl thianthrenium salt **1a** to allenyl thianthrenium salt **1** is achieved by the addition of saturated sodium bicarbonate solution. We speculate that the isomerization proceeds by a deprotonation–reprotonation sequence, but the exact mechanism and the strong dependence of the ratio on the base is currently not understood. Anion exchange with a KPF_6_ solution affords ATT^+^–PF_6_^–^, which was isolated as a free-flowing solid in 75% yield (Figure 2) by precipitation from dichloromethane with pentane. Four equivalents of propargyl alcohol were used to obtain a high yield. However, **1** can also be formed from one equivalent of propargyl alcohol by preforming propargyl triflate, albeit in only 41% yield (see the Supporting Information, page S12). Differential scanning calorimetry/thermogravimetric analysis (DSC-TGA) of purified ATT^+^–PF_6_^–^ shows no sign of decomposition until 127 °C. The crystal structure analysis of ATT^+^–PF_6_^–^ shows that the H atoms at C_13_ and C_15_ were identified experimentally and refined isotropically. The bond angle of S_1_–C_13_–C_14_ in **1** is 122°, indicating C_13_ to be sp^2^-hybridized. Attempts to synthesize other substituted allenyl thianthrenium salts following the same procedure were unsuccessful.

**Figure 2:**
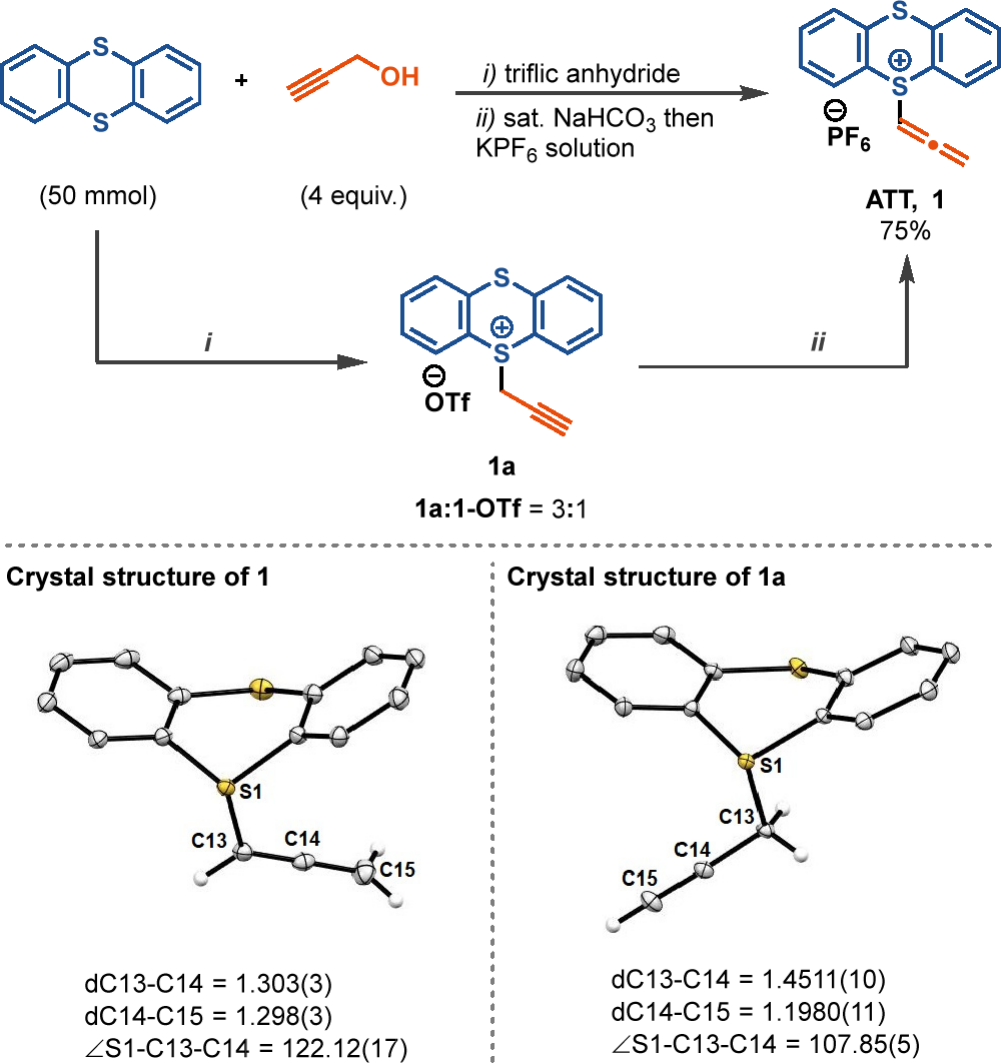
Synthesis of allenyl thianthrenium salt (ATT^+^–PF_6_^–^, **1**). Reaction conditions: (i) 1.2 equiv of triflic anhydride, 4.0 equiv of propargyl alcohol, 3.0 equiv of potassium carbonate, DCM (0.1 M), 4 Å molecular sieves, −78 °C to rt, 16 h; (ii) saturated NaHCO_3_ and then KPF_6_ solution. Crystal structure of ATT^+^**1** and PTT^+^**1a**: thermal ellipsoids are drawn at 50% probability. Counter anions are omitted for clarity. H atoms at C_13_ and C_15_ were found and refined isotropically.

Dimethyl propargyl sulfonium bromide can be used for annulation chemistry,^9−11^ but it often yields sulfide-containing products due to nucleophilic attack by the bromide counteranion on the sulfonium cation. In contrast to our work reported here where a direct nucleophilic attack occurs on the allenyl carbon C_14_ (Figure 2), the previous cyclization techniques relied upon the formation of sulphonium ylides at C_13_ of the propargyl salt, which subsequently yielded the allene adduct.^11^

The allenyl thianthrenium tetrafluoroborate salt **1-BF**_**4**_ gives one-step access (Scheme 1) to quinoxalines (**2**, **3**, **4**), benzoxazine (**5**), and dihydrobenzodioxine (**10**), as well as benzodioxepinones (**6**, **8**, **9**) and benzoxathiepinone (**7**). Traditionally, the synthesis of quinoxaline from 1,2-phenylenediamines requires harsh reaction conditions and Ni/Mn catalysts.^23−26^ Also, the cyclization with catechol leading to the formation of dihydrobenzodioxine (**10**) has previously been achieved by employing a Pd catalyst^27^ or with HgO.^28^ Benzodioxepinone (**6**), which was synthesized via a four-step process,^29,30^ can be obtained in a single step with reagent **1-BF**_**4**_. Similarly, the benzoxathiepinone product **7**, diflunisal-derived product **8**, and the benzodioxepinone product **9** are obtained from their parent (thio-)salicylic acid derivatives in a one-step process. For some compounds, the reagent **1-BF**_**4**_ with a tetrafluoroborate counterion provides a higher yield compared to **1** (see the Supporting Information, page S14) or **1a** (see the Supporting Information, page S34).

**Scheme 1:**
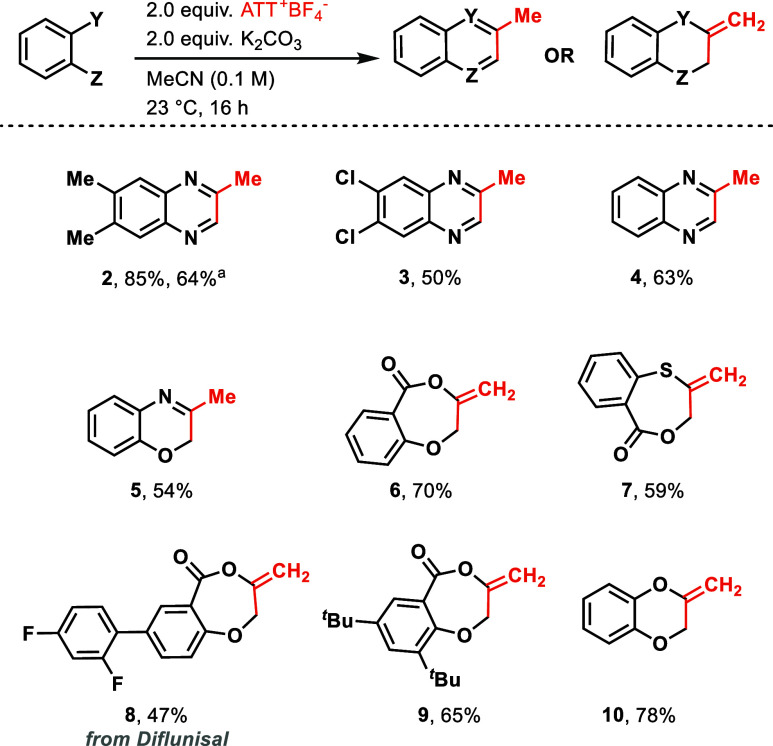
Scope of Aromatic Substrates for Annulation Reactions by ATT^+^-BF_4_^–^ Reactions were carried out at 0.2 mmol scale unless otherwise stated. (a) Reaction carried out at 1 mmol scale.

The mechanism of the reaction with allenylating reagent **1-BF**_**4**_ is most easily explained by a nucleophilic addition/substitution sequence, as shown in Scheme 2, although intermediates were not identified. Product identity is consistent with the initial attack of the stronger nucleophile at the electrophilic C_2_ position of the allenyl fragment with subsequent protonation of the incipient ylide. The resulting allyl thianthrenium fragment is expected to function as reactive electrophile for fast ring closure. Products **5**, **6**, and **7** were isolated as single constitutional isomers consistent with nucleophilic attack of the stronger nucleophile at the electrophilic allenyl C-2 before nucleophilic ring closure, e.g., nitrogen attack in favor of oxygen attack in **5** (Scheme 2).

**Scheme 2:**
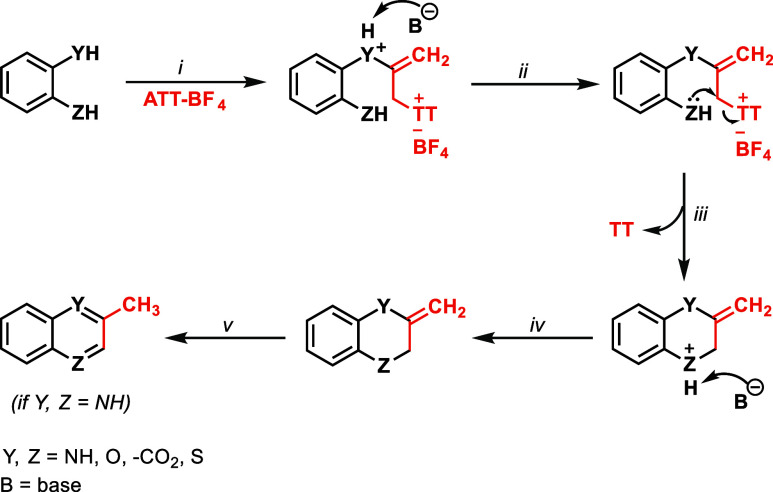
Proposed Mechanism for the Annulation Reaction of Aromatic Substrates with ATT^+^–BF_4_^–^: (i) Nucleophilic Addition, (ii) Proton Abstraction by Base, (iii) Nucleophilic Substitution, (iv) Deprotonation, and (v) Isomerization

Reagent **1-BF**_**4**_ can also be used for the cyclization of aminoalcohols like d-(+)-pseudoephedrine (Scheme 3A) to give a hemiketal product (**11**). In contrast to the cyclization products shown in Scheme 1, it appears that initial attack at the allenyl C_2_ position proceeds by the alcohol, not the amino functionality, which is possibly a consequence of intermolecular hydrogen bonding in pseudoephedrine.^31^ When 3-aminophenol was treated with **1-BF**_**4**_ in the presence of K_2_CO_3_ as a base, the *O*-homoacylation product was obtained (see the Supporting Information, page S24).

**Scheme 3:**
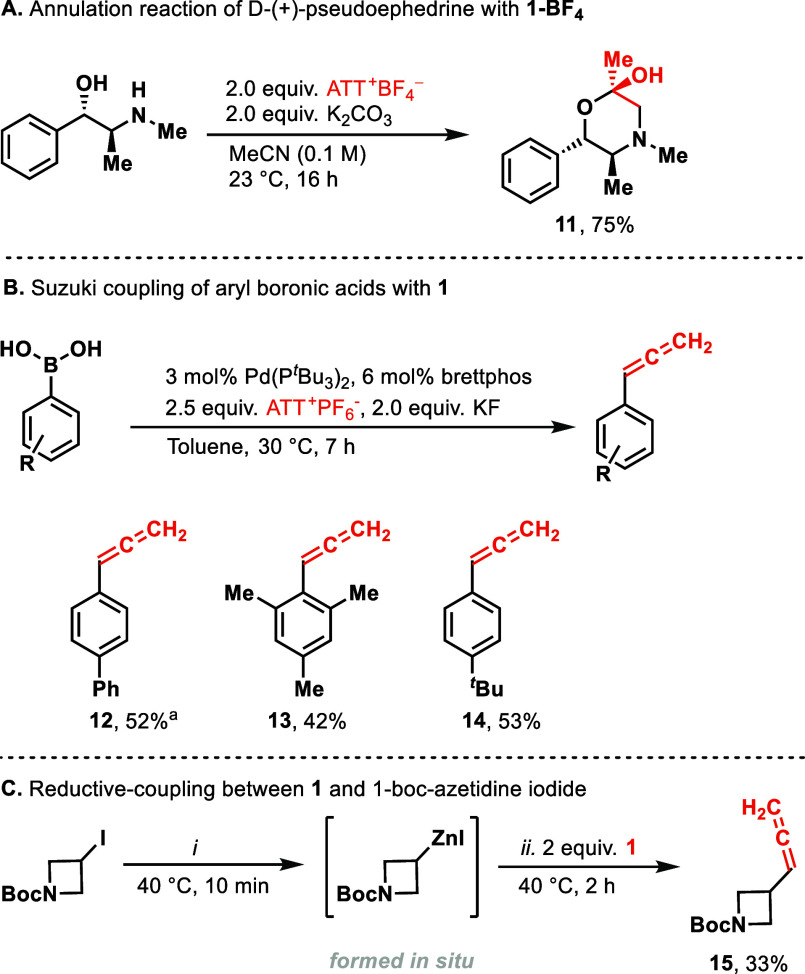
(A) Annulation Reaction of d-(+)-Pseudoephedrine with **1-BF**_**4**_ Carried out on 0.2 mmol Scale, (B) Suzuki Coupling of Aryl Boronic Acids with **1** Carried out on 0.5 mmol Scale (a) at Reaction Time of 12 h, and (C) Reductive Coupling between **1** and 1-Boc-azetidine Iodide Carried out on 0.5 mmol Scale under Reaction Conditions: (i) 5 mol % PdCl_2_(PPh_3_)_2_, 10 mol % Amphos, 1.5 equiv of Zn, 1.5 equiv of MgBr_2_, DMF (0.2 M), 40 °C, 10 min, and (ii) 2.0 equiv of ATT^+^–PF_6_^–^, DCM (0.11 M), 40 °C, 2 h

Reagent **1** is the first electrophilic allenyl sulfonium salt for cross-coupling reactions. Previously, propargyl bromide has been used for Kumada coupling reactions with Grignard reagents^16^ to incorporate an allenyl moiety into aromatic systems, often affording mixtures of propargyl and allenyl products depending on the substrate. When organotitanium reagents^32^ were used in place of Grignard reagents, allenyl products were obtained in good yields for a few sterically unhindered substrates. Suzuki coupling with reagent **1** in the presence of a palladium catalyst to generate the corresponding aryl–allene products **12**, **13**, and **14** were successful (Scheme 3B). No propargyl products were formed. The common side product is the formation of hydrodefunctionalized products. Although reagent **1** is the first of its kind to elicit this cross coupling reactivity, the reactivity in Suzuki reactions is not as robust as in conventional aryl–aryl or aryl–alkenyl coupling reactions under the reaction conditions reported here. For example, the reaction gives no yield for heteroaryl boronic acids, and several functional groups typically tolerated in Suzuki reactions result in significantly lower yields (see Figure S9). Although a large substrate scope with current conditions is beyond the scope of this initial investigation, the distinct reactivity of the reagent beyond previous conventional allenyl electrophiles bodes well for future reaction discovery with **1**.

A preliminary evaluation of reagent **1** for other cross coupling reactions afforded access to the allenyl–azetidine^33^ product **15** (Scheme 3C) via reductive cross-coupling. Similar to our report on reductive coupling of vinyl thianthrenium salt,^34^ the reaction proceeds via a Negishi coupling between reagent **1** and the alkyl zinc species formed *in situ* from the alkyl iodide and activated zinc dust. In the case of larger rings, like *N*-Boc-4-iodopiperidine, the β-hydride elimination product is formed predominantly (see Figure S13). Although a lower yield is observed when compared to that of the vinylthianthrenium reagent, the reactivity of **1** enables reaction chemistry not currently possible with other allenyl electrophiles.

In conclusion, we have developed a bench-stable allenyl thianthrenium salt **1** that can be prepared in one step from commercially available starting materials. Although **1** is currently not as versatile as common aryl or alkenyl electrophiles, as demonstrated by the smaller scope, for example, in Suzuki reactions, the distinct and enabling reactivity beyond conventional propargylating reagents, the lack of observed isomerization to afford product mixtures resulting from propargylated products, and its straightforward synthesis make **1** a promising candidate for future reaction development.

## Supporting Information Available

Experimental procedures and spectral data (PDF)
