# Supplementary material for: Allenyl Thianthrenium Salt: A Bench-Stable C3 Synthon for Annulation and Cross-Coupling Reactions
Source: J Am Chem Soc. 2024 Sep 24;146(40):27282–6. doi: 10.1021/jacs.4c10135 (PMC11468728; doi:10.1021/jacs.4c10135)
Supplement: Supplementary file 1 — ja4c10135_si_001.pdf [file ja4c10135_si_001.pdf]

SUPPORTING INFORMATION

Allenyl Thianthrenium Salt: A bench-stable C_3 synthon for Annulation and Cross-Coupling reactions

Srija Tewari,[1,2] Nicolai Klask,[1,2] Tobias Ritter*[1]

[1]Max-Planck-Institut für Kohlenforschung, Kaiser-Wilhelm-Platz 1, 45470 Mülheim an der Ruhr, Germany.

[2]Institute of Organic Chemistry, RWTH Aachen University, Landoltweg 1, 52074 Aachen, Germany.

*E-mail: ritter@kofo.mpg.de

TABLE OF CONTENTS

TABLE OF CONTENTS 1

MATERIALS AND METHODS 5

EXPERIMENTAL DATA 7

Synthesis of allenyl thianthrenium hexafluorophosphate (**1**) 7

Synthesis of allenyl thianthrenium tetrafluoroborate (**1-BF$_4$**) 10

Synthesis of propargyl thianthrenium triflate (**1a**) 11

Synthesis of allenyl thianthrenium salt **1** with one equivalent of propargyl alcohol 12

Annulation reactions with allenyl thianthrenium salt **1-BF$_4$** 12

General procedure for annulation reaction with allenyl thianthrenium salt **1-BF$_4$** 12

2,6,7-Trimethylquinoxaline (**2**) 13

6,7-Dichloro-(2-methyl)quinoxaline (**3**) 14

2-Methylquinoxaline (**4**) 14

3-Methylbenzoxazine (**5**) 15

Benzodioxepinone derivative **6** 15

Benzoxathiepinone derivative **7** 16

Diflunisal derivative **8** 17

(7,9-Di-*tert*-butyl)benzodioxepinone derivative **9** 17

Benzodioxine derivative **10** 18

Pseudoephedrine derivative **11** 19

Suzuki coupling reactions with allenyl thianthrenium salt **1** 20

General procedure for Suzuki coupling reaction with allenyl thianthrenium salt **1** 20

Allenyl-4-phenylbenzene (**12**) 21

Allenyl-(2,4,6-trimethyl)benzene (**13**) 22

Allenyl-(4-*tert*-butyl)benzene (**14**) 22

Reductive coupling reaction with allenyl thianthrenium salt **1** 23

3-Allenyl-1-boc-azetidine (**15**) 23

3-Aminophenol-derived O-homoacylation product **16** 24

Reaction optimization for Suzuki coupling 26

General procedure for Suzuki coupling with allenyl thianthrenium salt **1** 26

Optimization of yield as a function of catalyst 27

Optimization of yield as a function of base with different catalysts 27

Optimization of yield as a function of solvent 28

Optimization of yield as a function of time and equivalents of **1** 29

Optimization of yield as a function of temperature 29

Unsuccessful substrates for Suzuki coupling reaction with allenyl thianthrenium salt **1**........ 30

Unsuccessful substrates for reductive coupling reaction with allenyl thianthrenium salt **1** 32

General procedure for reductive coupling with **1**........ 32

Reductive coupling reaction of N-boc-4-iodopiperidine with **1** 33

Reactivity study of propargyl thianthrenium salt **1a**........ 34

Annulation reaction of catechol with propargyl thianthrenium tetrafluoroborate 34

Suzuki coupling of 4-biphenyl boronic acid with propargyl thianthrenium hexafluorophosphate........ 35

X-RAY CRYSTALLOGRAPHIC ANALYSIS........ 37

Allenyl thianthrenium hexafluorophosphate (ATT-PF_6, **1**) (CCDC 2336789) 37

Experimental........ 37

Propargyl thianthrenium triflate (PTT-OTf, **1a**) (CCDC 2336790) 40

Experimental........ 40

SPECTROSCOPIC DATA........ 44

^{1}H NMR of allenyl thianthrenium hexafluorophosphate (**1**) 44

^{13}C NMR of allenyl thianthrenium hexafluorophosphate (**1**)........ 45

^{19}F NMR of allenyl thianthrenium hexafluorophosphate (**1**) 46

^{1}H NMR of allenyl thianthrenium tetrafluoroborate (**1-BF$_4$**)........ 47

^{13}C NMR of allenyl thianthrenium tetrafluoroborate (**1-BF$_4$**)........ 48

^{19}F NMR of allenyl thianthrenium tetrafluoroborate (**1-BF$_4$**) 49

^{1}H NMR of propargyl thianthrenium triflate (**1a**) 50

^{13}C NMR of propargyl thianthrenium triflate (**1a**)........ 51

^{19}F NMR of propargyl thianthrenium triflate (**1a**) 52

^{1}H NMR of 2,6,7-trimethylquinoxaline (**2**)........ 53

^{13}C NMR of 2,6,7-trimethylquinoxaline (**2**)........ 54

^{1}H NMR of 6,7-dichloro-(2-methyl)quinoxaline (**3**) 55

^{13}C NMR of 6,7-dichloro-(2-methyl)quinoxaline (**3**) 56

^{1}H NMR of 2-methylquinoxaline (**4**) 57

^{13}C NMR of 2-methylquinoxaline (**4**) 58

^{1}H NMR of 3-methylbenzoxazine (**5**) 59

^{13}C NMR of 3-methylbenzoxazine (**5**) 60

HMBC of 3-methylbenzoxazine (**5**) 61

^{1}H NMR of benzodioxepinone derivative **6** 62

^{13}C NMR of benzodioxepinone derivative **6** 63

HMBC of benzodioxepinone derivative **6** 64

^{1}H NMR of benzoxathiepinone derivative **7** 65

^{13}C NMR of benzoxathiepinone derivative **7** 66

HMBC of benzoxathiepinone derivative **7** 67

^{1}H NMR of diflunisal derivative **8** 68

^{13}C NMR of diflunisal derivative **8** 69

^{19}F NMR of diflunisal derivative **8** 70

HMBC of diflunisal derivative **8** 71

^{1}H NMR of (7,9-di-*tert*-butyl)benzodioxepinone derivative **9** 72

^{13}C NMR of (7,9-di-*tert*-butyl)benzodioxepinone derivative **9** 73

HMBC of (7,9-di-*tert*-butyl)benzodioxepinone derivative **9** 74

^{1}H NMR of benzodioxine derivative **10** 75

^{13}C NMR of benzodioxine derivative **10** 76

^{1}H NMR of pseudoephedrine derivative **11** 77

^{13}C NMR of pseudoephedrine derivative **11** 78

NOESY of pseudoephedrine derivative **11** 79

HMBC of pseudoephedrine derivative **11** 80

^{1}H NMR of allenyl-4-phenylbenzene (**12**) 81

^{13}C NMR of allenyl-4-phenylbenzene (**12**) 82

^{1}H NMR of allenyl-(2,4,6-trimethyl)benzene (**13**) 83

^{13}C NMR of allenyl-(2,4,6-trimethyl)benzene (**13**) 84

^{1}H NMR of allenyl-(4-*tert*-butyl)benzene (**14**) 85

^{13}C NMR of allenyl-(4-*tert*-butyl)benzene (**14**) 86

^{1}H NMR of 3-allenyl-1-boc-azetidine (**15**) 87

^{13}C NMR of 3-allenyl-1-boc-azetidine (**15**) 88

REFERENCES 89

MATERIALS AND METHODS

All air- and moisture-insensitive reactions were carried out under an ambient atmosphere and monitored by thin layer chromatography (TLC). Air- and moisture-sensitive manipulations were performed using standard Schlenk techniques under an atmosphere of argon or inside the glovebox under a nitrogen atmosphere. Concentration under reduced pressure was performed by rotary evaporation between 25 °C – 40 °C at an appropriate pressure. Purified compounds were further dried under a high vacuum (10^{-6}–10^{-3} bar) if required. Yields refer to purified and spectroscopically pure compounds unless otherwise stated. 23 °C is defined as a temperature range from 22 °C – 25 °C.

Solvents

Dry dichloromethane (DCM), dry acetonitrile (MeCN), and dry toluene were obtained from *Phoenix Solvent Drying Systems*. Dry dimethylformamide (DMF) was purchased from *Sigma-Aldrich*. All deuterated solvents were purchased from *Euriso Top.*

Chromatography

Thin layer chromatography (TLC) was performed using EMD TLC plates pre-coated with 250 μm thickness silica gel 60 F_{254} plates and visualized by fluorescence quenching under UV light and $KMnO_4$ stain. Flash column chromatography was performed using pre-packed cartridges (Biotage Sfär Silica, 60 µm particle size) and self-prepared silver impregnated silica gel (40–63 µm particle size). Silver impregnated silica was prepared by dissolving 5.5 g of silver nitrate in 30 mL distilled water in a beaker. The aqueous solution was transferred to a mortar containing 50 g of silica. The mixture was ground in the mortar with a pestle for 5 min. The mixture was transferred into a beaker and dried in an oven at 150 °C for 1 h.[1] The resulting colorless powder was stored in the beaker wrapped in aluminum foil. The detailed solvent composition of the eluents is given for every compound individually.

Spectroscopy and Instruments

NMR spectra were recorded on either a Bruker AV600 spectrometer operating at 600 MHz and 150 MHz for ^{1}H and ^{13}C acquisitions, respectively, or a Bruker Ascend™ 500 spectrometer operating at 500 MHz, 471 MHz, and 126 MHz, for ^{1}H, ^{19}F, and ^{13}C acquisitions, respectively, or a Bruker Ultrashield™ 300 spectrometer operating at 300 MHz, 282 MHz and 75 MHz for 1H, 19F and 13C acquisitions, respectively. Chemical shifts are reported in ppm with the solvent resonance as the internal standard. For ^{1}H NMR: $CDCl_3$, δ 7.26; CD_3CN, δ 1.94. For ^{13}C NMR: $CDCl_3$, δ 77.16; CD_3CN, δ 118.3, 1.3.[2] Data is reported as follows: s = singlet, d = doublet, t = triplet, q = quartet, m = multiplet, br = broad; coupling constants in Hz; integration. UV-Vis absorption spectra were recorded on a UV-2600 Shimadzu spectrophotometer. Cyclic voltammetry was conducted using an Autolab PGSTAT204 potentiostat.

Starting materials

All substrates were used as received from *Sigma Aldrich*, *TCI*, *Thermo Fischer Scientific* and *BLD Pharm*. The palladium catalyst $Pd(P^tBu_3)_2$ was purchased from *Sigma Aldrich*. The amphos and brettphos ligands were purchased from *BLD Pharm*. Care was taken to avoid contact of the palladium catalyst $Pd(P^tBu_3)_2$ or dry DMF with the ambient atmosphere and it was stored under a nitrogen atmosphere once the shipping container was opened.

EXPERIMENTAL DATA

Synthesis of allenyl thianthrenium hexafluorophosphate (1)

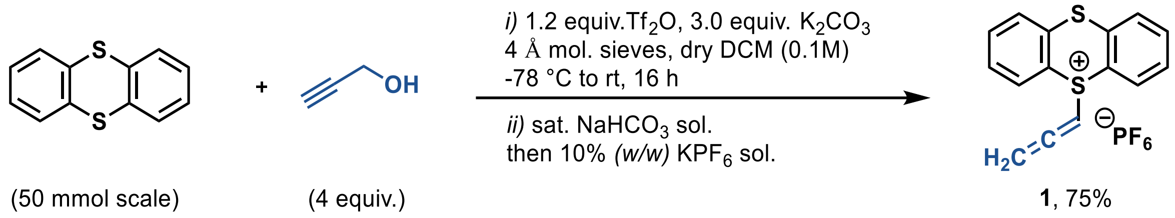

Under an argon atmosphere, a 1 L double-necked round-bottom flask was charged with thianthrene (10.8 g, 50.0 mmol, 1.00 equiv.), potassium carbonate (20.7 g, 0.150 mol, 3.00 equiv.) and activated 4 Å molecular sieves. Then dry DCM (500 mL, c = 0.1 M) was added and the reaction mixture was cooled to –78 °C. Subsequently, triflic anhydride (10.1 mL, 16.9 g, 60.0 mmol, 1.20 equiv.) was added dropwise over 5 minutes while stirring, followed by propargyl alcohol (11.6 mL, 11.2 g, 0.200 mol, 4.00 equiv.). After 30 minutes, the cooling bath was removed and the resulting purple-colored reaction mixture was stirred for 16 hours at 23 °C. After the completion of the reaction indicated by brown-coloration, saturated aqueous $NaHCO_3$ solution (250 mL) was added to the reaction mixture, and the mixture was stirred for 45 minutes at 23 °C. The reaction mixture was filtered and poured into a separating funnel, the phases were separated and the aqueous phase was extracted with DCM (2 × 100 mL). The combined organic phase was washed with a saturated aqueous $NaHCO_3$ solution (3 × 150 mL). Then, the organic layer was partially concentrated to 250 mL and was washed with a 10% (w/w) aqueous KPF_6 solution (3 × 200 mL). The organic phase was further washed with a saturated aqueous $NaHCO_3$ solution (4 × 100 mL) to convert any remaining propargyl thiathrenium salt into its allenyl isomer. Then, the organic phase was dried over Na_2SO_4 and filtered. The solvent was evaporated under reduced pressure resulting in a solid, which was washed with pentane (2 × 100 mL). Then, the solid was dissolved in DCM (70 mL), and pentane (300 mL) was slowly added while stirring, causing precipitation of a solid that was collected by filtration. The collected solid was washed with EtOAc (2 × 70 mL) and pentane (2 × 75 mL), followed by drying under vacuum to obtain the desired product **1** as a yellow-ochre colored solid (15.0 g, 37.4 mmol, 75%).

R_f = 0.38 (DCM/MeOH, 9:1 (v:v)).

NMR Spectroscopy:

^{1}H NMR (500 MHz, CD_3CN, 23 ºC) δ 8.14 (dd, *J* = 8.0, 1.3 Hz, 2H), 7.97 (dd, *J* = 8.0, 1.3 Hz, 2H), 7.83 (td, *J* = 7.7, 1.4 Hz, 2H), 7.72 (td, *J* = 7.7, 1.4 Hz, 2H), 6.22 (t, *J* = 6.0 Hz, 1H), 5.49 (d, *J* = 6.0 Hz, 2H).

^{13}C{^{1}H} NMR (126 MHz, CD_3CN, 23 ºC) δ 210.3, 137.5, 135.7, 134.9, 131.2, 130.5, 118.6, 89.2, 83.1.

^{19}F NMR (471 MHz, CD_3CN, 23 ºC) δ –73.0 Hz (d, *J* = 706.5 Hz).

HRMS-ESIpos (m/z) calc'd for $C_{15}H_{11}S_2$ $[M]^+$, 255.0297; found, 255.0297; deviation: 0.0 ppm.

IR (neat) : ν_{max} (cm^{-1}) = 3074, 3035, 2993, 1964, 1937, 1717, 1570, 1450, 1292, 1265, 903, 880, 829, 756,

706, 660.

Melting point: 139 –141 °C.

Elemental analysis calc'd (%) for $C_{15}H_{11}S_2PF_6$: C 45.00, H 2.77, S 16.02; found: C 44.84, H 2.79, S 15.98.

No decomposition was observed after storing a sample of **1** between 20 °C and 30 °C under air for one year. In the figure below, we show the ^{1}H NMR spectrum of a sample (from a 15-g scale synthesis batch) that was stored in a 20 mL vial without any further precaution for one year.

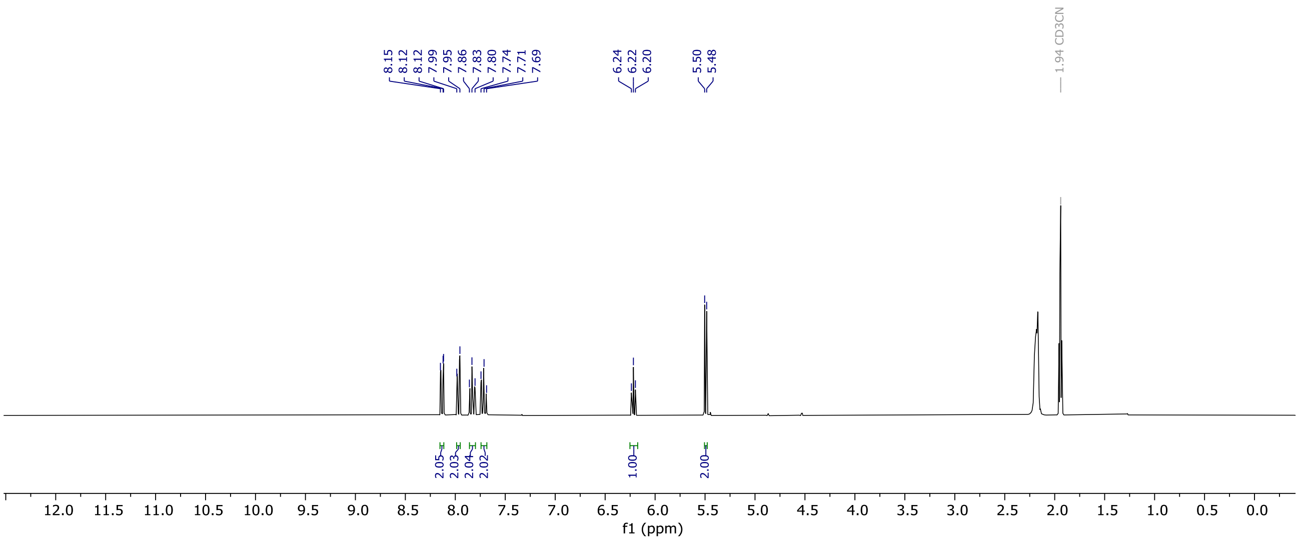

Figure S1. ^{1}H NMR spectrum of a sample of ATT-PF_6 (**1**) stored for one year, CD_3CN, 300 MHz, 23 ºC.

DSC-TGA analysis

A blank group and an experimental group were tested under an argon purge. Both groups used aluminum crucibles with pierced lids (open crucibles) as a container.

Ar gas flow velocity: 50 mL/min

Heating region: from 40 °C to 300 °C

Heating rate: 3K/min

ATT-PF_6 (**1**, powder, 10.12 mg, 0.0253 mmol) was placed in an aluminium crucible for the DSC-TGA measurement. Decomposition of **1** begins at 127 °C.

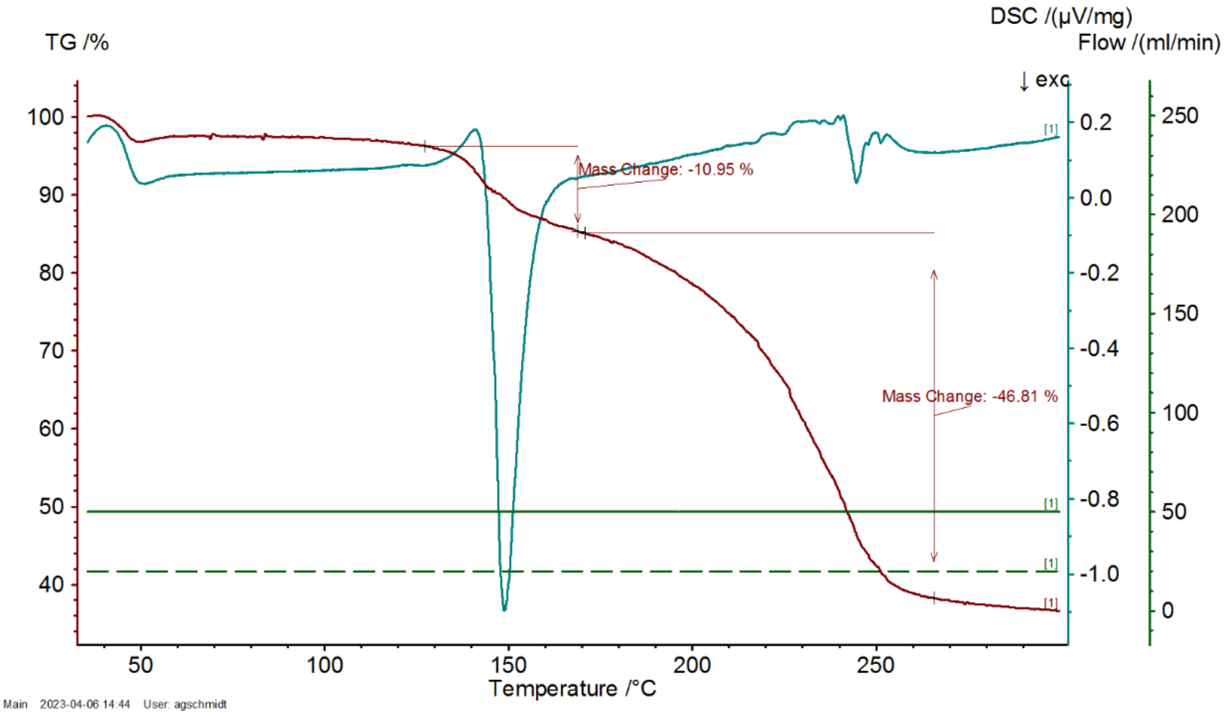

Figure S2. DSC-TGA analysis of **1**.

UV analysis

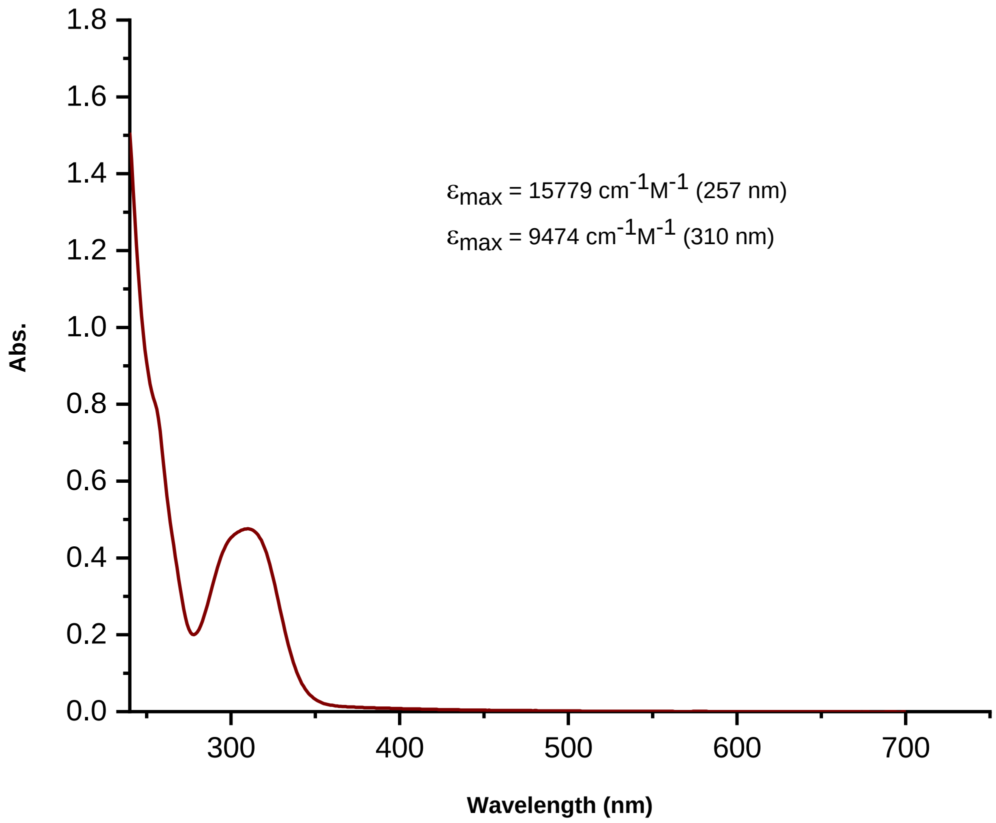

Figure S3. UV-Vis absorption spectrum of **1** in solution (c = 5 × 10^{-5} M in MeCN, 23 ºC).

CV analysis

Cyclic voltammetry was conducted using a potentiostat equipped with a glassy carbon working electrode, an Ag/AgCl reference electrode, and a Pt counter electrode. Samples containing 10 mM of **1** and 0.1 M of tetrabutylammonium tetrafluoroborate were prepared in dry MeCN and degassed before measurement. Voltammograms were measured at 0.1 V/s scan rate. Potential values were converted to SCE subtracting 0.047 V according to tabulated conversions. Potentials for irreversible waves were estimated at half the maximum current ($E_{p/2}$), as previously described by Nicewicz. [3]

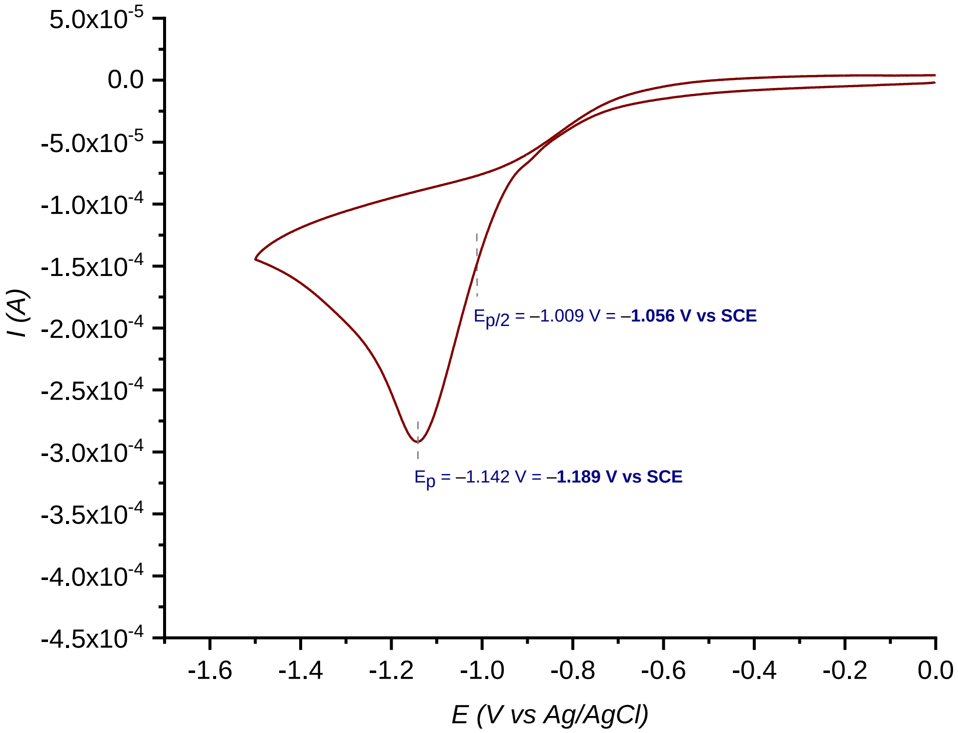

Figure S4. Cyclic voltammogram of **1**.

Synthesis of allenyl thianthrenium tetrafluoroborate (1-BF_4)

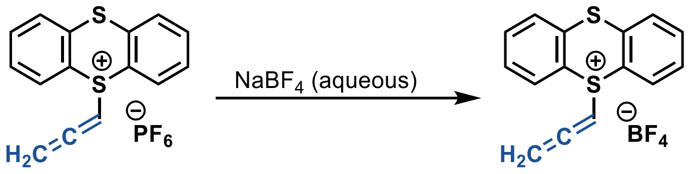

Under air, allenyl thianthrenium hexafluorophosphate **1** (1.46 g, 3.64 mmol, 1.00 equiv.) was dissolved in DCM (100 mL, c = 4 × 10^{-2} M). The solution was washed with aqueous sodium tetrafluoroborate solution (c = 10% w/w, 4 × 100 mL), dried over Na_2SO_4, filtered and the solvent was evaporated under reduced pressure. The crude solid was washed with EtOAc (2 × 20) and pentane (2 × 30), followed by drying under vacuum to afford **1-BF_4** as an off-white solid (997 mg, 2.91 mmol, 80%).

R*f* = 0.25 (DCM/MeOH, 9:1 (v/v))

NMR Spectroscopy:

^{1}H NMR (500 MHz, CD_3CN, 23 ºC) δ 8.14 (dd, *J* = 8.0, 1.4 Hz, 2H), 7.97 (dd, *J* = 8.0, 1.3 Hz, 2H), 7.83 (td, *J* = 7.7, 1.4 Hz, 2H), 7.72 (td, *J* = 7.7, 1.4 Hz, 2H), 6.22 (t, *J* = 6.0 Hz, 1H), 5.49 (d, *J* = 6.0 Hz, 2H).

^{13}C{^{1}H} NMR (126 MHz, CD_3CN, 23 ºC) δ 210.3, 137.5, 135.8, 135.0, 131.2, 130.6, 118.7, 89.3, 83.1.

^{19}F NMR (471 MHz, CD_3CN, 23 ºC) δ –151.2.

HRMS-ESIpos (m/z) calc'd for $C_{15}H_{11}S_2$ $[M]^+$, 255.0297; found, 255.0296; deviation: +0.1 ppm.

Melting point: 146 – 148 °C.

Synthesis of propargyl thianthrenium triflate (1a)

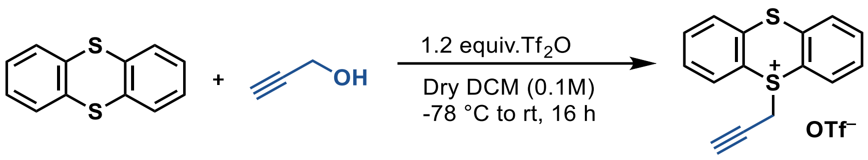

Under an argon atmosphere, a 50 mL double-necked round-bottom flask was charged with thianthrene (216 mg, 1.00 mmol, 1.00 equiv.).Then dry DCM (10 mL, c = 0.1 M) was added and the solution was cooled to –78 °C. Subsequently, triflic anhydride (0.202 mL, 339 mg, 1.20 mmol, 1.20 equiv.) was added dropwise while stirring, followed by propargyl alcohol (0.233 mL, 224 mg, 4.00 mmol, 4.00 equiv.). After 10 minutes, the cooling bath was removed and the resulting purple-colored reaction mixture was stirred for 16 hours at 23 °C. Diethyl ether (30 mL) was slowly added to the resulting brown-colored reaction mixture while stirring, causing the precipitation of a solid which was washed with diethyl ether (2 × 20 mL), followed by drying under vacuum to afford **1a** as a crystalline off-white solid (385 mg, 0.952 mmol, 95%).

R*f* = 0.25 (DCM/MeOH, 9:1, (v/v)).

NMR Spectroscopy:

^{1}H NMR (500 MHz, CD_3CN, 23 ºC) δ 8.15 (dd, *J* = 8.0, 1.4 Hz, 2H), 7.97 (dd, *J* = 8.0, 1.3 Hz, 2H), 7.86 (td, *J* = 7.7, 1.4 Hz, 2H), 7.73 (td, *J* = 7.7, 1.3 Hz, 2H), 4.55 (d, *J* = 2.7 Hz, 2H), 2.93 (t, *J* = 2.7 Hz, 1H).

^{13}C{^{1}H} NMR (126 MHz, CD_3CN, 23 ºC) δ 137.1, 136.2, 135.9, 131.1, 130.5, 122.2 (q, *J* = 315 Hz), 116.9, 81.8, 71.3, 33.2.

^{19}F NMR (471 MHz, CD_3CN, 23 ºC) δ –79.3.

HRMS-ESIpos (m/z) calc'd for $C_{15}H_{11}S_2$ $[M]^+$, 255.0297; found, 255.0296; deviation: +0.5 ppm.

Melting point: 143 – 145 °C.

Synthesis of allenyl thianthrenium salt 1 with one equivalent of propargyl alcohol

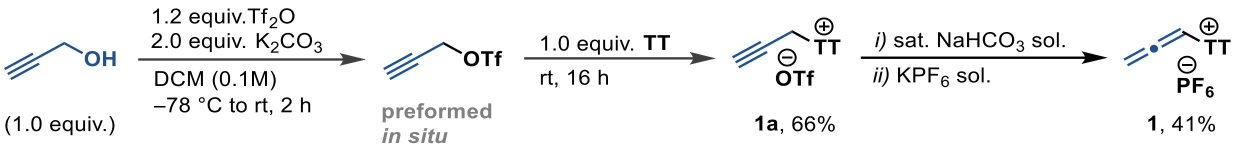

Under an argon atmosphere, a 50 mL double-necked round-bottom flask containing a Teflon-coated magnetic stirring bar was charged with potassium carbonate (276 mg, 2.00 mmol, 2.00 equiv.) and dry DCM (10 mL, c = 0.1 M). The solution was cooled to –78 °C and triflic anhydride (0.202 mL, 339 mg, 1.20 mmol, 1.20 equiv.) was added dropwise, followed by propargyl alcohol (0.058 mL, 56.1 mg, 1.00 mmol, 1.00 equiv.). After 30 minutes, the cooling bath was removed and the reaction mixture was stirred for 2 hours at 23 °C to pre-form the propargyl triflate. After 2 hours, thianthrene (**TT**) (216 mg, 1.00 mmol, 1.00 equiv.) was added at once and the reaction mixture was stirred for 16 hours at 23 °C. After the completion of the reaction, indicated by brown-coloration, the reaction mixture was filtered and diethyl ether (50 mL) was slowly added to the filtrate while stirring, causing precipitation of a solid. The solid was collected by filtration, washed with diethyl ether (2 × 20 mL) and dried under vacuum to afford **1a** as a crystalline off-white solid (265 mg, 0.655 mmol, 66% yield).

*[Note: When the above synthesis of **1a** from one equivalent of propargyl alcohol was carried out in absence of K_2CO_3, **1a** was isolated with 48% yield.]*

Propargyl thianthrenium triflate **1a** was further dissolved in DCM (15 mL) and saturated aqueous $NaHCO_3$ solution (15 mL) was added. The reaction mixture was poured into a separating funnel, shaken vigorously, the phases were separated and the aqueous phase was extracted with DCM (10 mL). The combined organic phase was washed with a saturated aqueous $NaHCO_3$ solution (2 × 20 mL) and then with a 10% (w/w) aqueous KPF_6 solution (3 × 20 mL). The organic phase was further washed with a saturated aqueous $NaHCO_3$ solution (4 × 20 mL), dried over $NaSO_4$ and filtered. The solvent was evaporated under reduced pressure resulting in a solid. The solid was dissolved in DCM (10 mL), followed by slow addition of pentane (50 mL) while stirring, causing precipitation of a solid. The solid was collected by filtration, and was further washed with EtOAc (2 × 10 mL) and pentane (2 × 20 mL). The purified solid was dried under vacuum to afford **1** as a yellow-ochre colored solid (163 mg, 0.407 mmol, 41%). ^{1}H NMR, ^{13}C NMR, ^{19}F NMR, and HRMS data are consistent with the analytical data reported for compound **1** when the reaction was carried out on a 50 mmol scale.

Annulation reactions with allenyl thianthrenium salt 1-BF4

General procedure for annulation reaction with allenyl thianthrenium salt 1-BF4

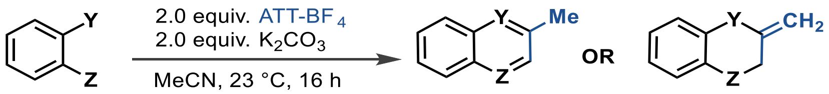

Under air, a 20 mL borosilicate vial containing a Teflon-coated stirring bar, was charged with the starting

material (0.200 mmol, 1.00 equiv.), allenyl thianthrenium tetrafluoroborate salt **1-BF$_4$** (137 mg, 0.400 mmol, 2.00 equiv.) and potassium carbonate (55.3 mg, 0.400 mmol, 2.00 equiv.) at 23 °C. Then, dry MeCN (2.0 mL, c = 0.10 M) was added [*dry MeCN was used for all the annulation reactions to ensure that the moisture content in the solvent remained same*]. The resulting reaction mixture was stirred at 23 °C for 16 hours. Then, the solvent was concentrated under reduced pressure. The resulting residue was purified by column chromatography on silica gel to afford the desired product.

2,6,7-Trimethylquinoxaline (2)

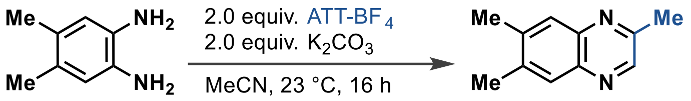

2, 85%

Under air, a 20 mL borosilicate vial was charged with 4,5-dimethyl-(1,2-diamino)benzene (27.2 mg, 0.200 mmol, 1.00 equiv.), allenyl thianthrenium tetrafluoroborate salt **1-BF$_4$** (137 mg, 0.400 mmol, 2.00 equiv.) and potassium carbonate (55.3 mg, 0.400 mmol, 2.00 equiv.) at 23 °C. MeCN (2.0 mL, c = 0.10 M) was added, and the resulting reaction mixture was stirred at 23 °C for 16 hours. Then, the solvent was concentrated under reduced pressure. The resulting residue was purified by column chromatography on silica gel eluting with DCM / MeOH (100% DCM – 20:1, v/v) to afford the desired product as a pale yellow solid (29.4 mg, 0.171 mmol, 85%).

R*$_f$* = 0.44 (DCM/MeOH, 9:1, (v/v)).

NMR Spectroscopy:

^{1}H NMR (500 MHz, $CDCl_3$, 23 ºC) δ 8.62 (s, 1H), 7.79 (s, 1H), 7.74 (s, 1H) 2.72 (s, 3H), 2.47 (s, 6H).

^{13}C{^{1}H} NMR (126 MHz, $CDCl_3$, 23 ºC) δ 152.8, 145.2, 141.2, 140.6, 140.1, 139.4, 128.3, 127.9, 22.6, 20.5, 20.3.

HRMS-EI(m/z) calc'd for $C_{11}H_{12}N_2$ $[M]^+$, 172.0995; found, 172.0997 ; deviation: –1.3 ppm.

Procedure for 1 mmol scale reaction

Under air, a 20 mL borosilicate vial was charged with 4,5-dimethyl-(1,2-diamino)benzene (136 mg, 1.00 mmol, 1.00 equiv.), allenyl thianthrenium tetrafluoroborate salt **1-BF$_4$** (684 mg, 2.00 mmol, 2.00 equiv.) and potassium carbonate (276 mg, 2.00 mmol, 2.00 equiv.) at 23 °C. MeCN (10 mL, c = 0.1 M) was added, and the resulting reaction mixture was stirred at 23 °C for 16 hours. Then, the solvent was concentrated under reduced pressure. The resulting residue was purified by column chromatography on silica gel eluting with DCM / MeOH (100% DCM – 20:1, v/v) to afford the desired product as a yellow solid (109 mg, 0.639 mmol, 64%). ^{1}H NMR, ^{13}C NMR, and HRMS data are consistent with the analytical data reported for compound **2** when the reaction was carried out on a 0.2 mmol scale.

6,7-Dichloro-(2-methyl)quinoxaline (3)

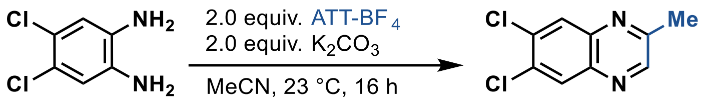

3, 50%

Under air, a 20 mL borosilicate vial was charged with 4,5-dichloro-(1,2-diamino)benzene (35.4 mg, 0.200 mmol, 1.00 equiv.), allenyl thianthrenium tetrafluoroborate salt **1-BF₄** (137 mg, 0.400 mmol, 2.00 equiv.) and potassium carbonate (55.3 mg, 0.400 mmol, 2.00 equiv.) at 23 °C. MeCN (2.0 mL, c = 0.10 M) was added, and the resulting reaction mixture was stirred at 23 °C for 16 hours. Then, the solvent was concentrated under reduced pressure. The resulting residue was purified by column chromatography on silica gel eluting with DCM / MeOH (100% DCM – 20:1, v/v) to afford the desired product as a pale yellow solid (21.1 mg, 0.099 mmol, 50%).

R*f* = 0.34 (DCM/MeOH, 9:1, (v/v)).

NMR Spectroscopy:

^{1}H NMR (500 MHz, $CDCl_3$, 23 ºC) δ 8.72 (s, 1H), 8.18 (s, 1H), 8.13 (s, 1H), 2.76 (s, 3H).

^{13}C{^{1}H} NMR (126 MHz, $CDCl_3$, 23 ºC) δ 155.3, 147.2, 141.1, 139.9, 134.7, 133.6, 130.0, 129.6, 22.8.

HRMS-EI(m/z) calc'd for $C_9H_6N_2Cl_2$ $[M]^+$, 211.9902; found, 211.9905; deviation: –1.1 ppm.

Procedure for reaction with ATT-PF_6 instead of ATT-BF_4:

Under air, a 20 mL borosilicate vial was charged with 4,5-dichloro-(1,2-diamino)benzene (35.4 mg, 0.200 mmol, 1.00 equiv.), allenyl thianthrenium hexafluorophosphate salt **1** (160 mg, 0.400 mmol, 2.00 equiv.) and potassium carbonate (55.3 mg, 0.400 mmol, 2.00 equiv.) at 23 °C. MeCN (2.0 mL, c = 0.10 M) was added, and the resulting reaction mixture was stirred at 23 °C for 16 hours. Then, the solvent was concentrated under reduced pressure. The resulting residue was purified by column chromatography on silica gel eluting with DCM / MeOH (100% DCM – 20:1, v/v) to afford the desired product as a pale yellow solid (14.0 mg, 0.066 mmol, 33%). ^{1}H NMR, ^{13}C NMR, and HRMS data are consistent with the analytical data reported for compound **3** when the reaction was carried out with **1-BF_4**.

2-Methylquinoxaline (4)

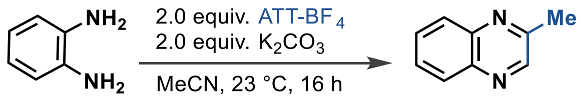

4, 63%

Under air, a 20 mL borosilicate vial was charged with 1,2-diaminobenzene (21.6 mg, 0.200 mmol, 1.00 equiv.), allenyl thianthrenium tetrafluoroborate salt **1-BF_4** (137 mg, 0.400 mmol, 2.00 equiv.) and potassium carbonate (55.3 mg, 0.400 mmol, 2.00 equiv.) at 23 °C. MeCN (2.0 mL, c = 0.10 M) was added, and the resulting reaction mixture was stirred at 23 °C for 16 hours. Then, the solvent was concentrated under

reduced pressure. The resulting residue was purified by column chromatography on silica gel eluting with DCM / MeOH (100% DCM – 20:1, v/v) to afford the desired product as a pale yellow oil (18.2 mg, 0.126 mmol, 63%).

R*f* = 0.47 (DCM/MeOH, 9:1, (v/v)).

NMR Spectroscopy:

^{1}H NMR (500 MHz, $CDCl_3$, 23 ºC) δ 8.74 (s, 1H), 8.07 (dd, *J* = 8.0, 1.9 Hz, 1H), 8.02 (dd, *J* = 8.0, 1.9 Hz, 1H), 7.76 – 7.69 (m, 2H), 2.78 (s, 3H).

^{13}C{^{1}H} NMR (126 MHz, $CDCl_3$, 23 ºC) δ 153.9, 146.2, 142.2, 141.1, 130.2, 129.3, 129.1, 128.8, 22.7.

HRMS-ESIpos(m/z) calc'd for $C_9H_9N_2$ $[M+H]^+$, 145.0760; found, 145.0762; deviation: –1.2 ppm.

3-Methylbenzoxazine (5)

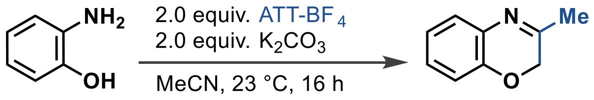

5, 54%

Under air, a 20 mL borosilicate vial was charged with 2-aminophenol (21.8 mg, 0.200 mmol, 1.00 equiv.), allenyl thianthrenium tetrafluoroborate salt **1-BF$_4$** (137 mg, 0.400 mmol, 2.00 equiv.) and potassium carbonate (55.3 mg, 0.400 mmol, 2.00 equiv.) at 23 °C. MeCN (2.0 mL, c = 0.10 M) was added, and the resulting reaction mixture was stirred at 23 °C for 16 hours. Then, the solvent was concentrated under reduced pressure. The resulting residue was purified by column chromatography on silica gel eluting with DCM / MeOH (100% DCM – 20:1, v/v) to afford the desired product as a dark yellow oil (16.0 mg, 0.109 mmol, 54%). The constitution of the product was confirmed from HMBC data.

R*f* = 0.44 (DCM/MeOH, 9:1, (v/v)).

NMR Spectroscopy:

^{1}H NMR (500 MHz, $CDCl_3$, 23 ºC) δ 7.25 (dd, *J* = 7.5, 1.8 Hz, 1H), 7.09 (td, *J* = 7.7, 1.8 Hz, 1H), 6.96 (td, *J* = 8.3, 1.5 Hz, 1H), 6.83 (dd, *J* = 8.0, 1.4 Hz, 1H), 4.54 (s, 2H), 2.15 (s, 3H).

^{13}C{^{1}H} NMR (126 MHz, $CDCl_3$, 23 ºC) δ 163.4, 146.0, 132.8, 128.2, 126.9, 122.2, 115.5, 65.3, 23.5.

HRMS-EI(m/z) calc'd for C_9H_9NO $[M]^+$, 147.0679; found, 147.0680; deviation: –1.1 ppm.

Benzodioxepinone derivative 6

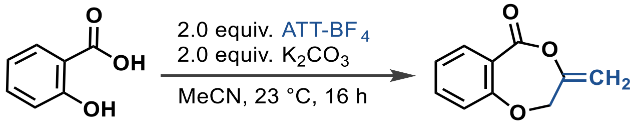

6, 70%

Under air, a 20 mL borosilicate vial was charged with salicylic acid (27.6 mg, 0.200 mmol, 1.00 equiv.), allenyl

thianthrenium tetrafluoroborate salt **1-BF4** (137 mg, 0.400 mmol, 2.00 equiv.) and potassium carbonate (55.3 mg, 0.400 mmol, 2.00 equiv.) at 23 °C. MeCN (2.0 mL, c = 0.10 M) was added, and the resulting reaction mixture was stirred at 23 °C for 16 hours. Then, the solvent was concentrated under reduced pressure. The resulting residue was purified by column chromatography on silica gel eluting with pentane / EtOAc (100% pentane – 20:1, v/v) to afford the desired product as a colorless oil (24.6 mg, 0.139 mmol, 70%). The constitution of the product was confirmed from HMBC data.

R*f* = 0.44 (pentane/EtOAc, 9:1, (v/v)).

NMR Spectroscopy:

^{1}H NMR (500 MHz, $CDCl_3$, 23 ºC) δ 7.93 (dd, *J* = 8.0, 1.8 Hz, 1H), 7.50 (ddd, *J* = 8.8, 7.3, 1.8 Hz, 1H), 7.14 (ddd, *J* = 8.2, 7.3, 1.2 Hz, 1H), 7.04 (dd, *J* = 8.3, 1.2 Hz, 1H), 5.12 (d, *J* = 2.0 Hz, 1H), 4.88 (d, *J* = 2.0 Hz, 1H), 4.75 (s, 2H).

^{13}C{^{1}H} NMR (126 MHz, $CDCl_3$, 23 ºC) δ 165.6, 156.6, 151.1, 135.1, 133.9, 123.2, 120.9, 119.3, 105.7, 71.8.

HRMS-EI(m/z) calc'd for $C_{10}H_8O_3$ $[M]^+$, 176.0468; found, 176.0469; deviation: –0.5 ppm.

Benzoxathiepinone derivative 7

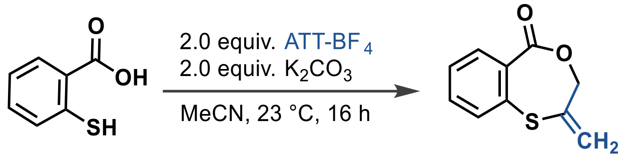

7, 59%

Under air, a 20 mL borosilicate vial was charged with thiosalicylic acid (30.8 mg, 0.200 mmol, 1.00 equiv.), allenyl thianthrenium tetrafluoroborate salt **1-BF4** (137 mg, 0.400 mmol, 2.00 equiv.) and potassium carbonate (55.3 mg, 0.400 mmol, 2.00 equiv.) at 23 °C. MeCN (2.0 mL, c = 0.10 M) was added, and the resulting reaction mixture was stirred at 23 °C for 16 hours. Then, the solvent was concentrated under reduced pressure. The resulting residue was purified by column chromatography on silica gel eluting with pentane / EtOAc (100% pentane – 20:1, v/v) to afford the desired product as a colorless solid (22.6 mg, 0.118 mmol, 59%). The constitution of the product was confirmed from HMBC data.

R*f* = 0.38 (pentane/EtOAc, 5:1, (v/v)).

NMR Spectroscopy:

^{1}H NMR (500 MHz, $CDCl_3$, 23 ºC) δ 7.77 (dd, *J* = 8.1, 1.4 Hz, 1H), 7.51 – 7.43 (m, 3H), 5.33 (s, 1H), 5.26 (s, 1H), 4.75 (s, 2H).

^{13}C{^{1}H} NMR (126 MHz, $CDCl_3$, 23 ºC) δ 169.8, 140.9, 134.9, 133.0, 132.3, 132.0, 129.5, 129.3, 111.7, 70.5.

HRMS-EI(m/z) calc'd for $C_{10}H_8O_2S_1$ $[M]^+$, 192.0240; found, 192.0243; deviation: –1.6 ppm.

Diflunisal derivative 8

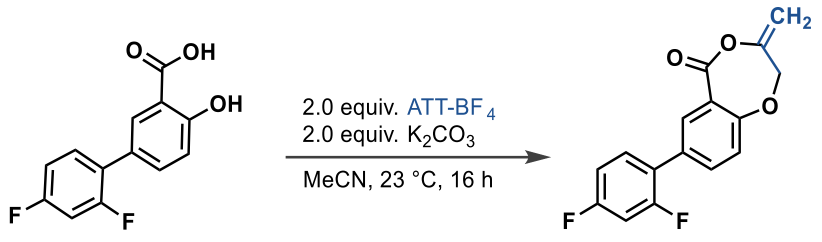

8, 47%

Under air, a 20 mL borosilicate vial was charged with diflunisal (50.0 mg, 0.200 mmol, 1.00 equiv.), allenyl thianthrenium tetrafluoroborate salt **1-BF$_4$** (137 mg, 0.400 mmol, 2.00 equiv.) and potassium carbonate (55.3 mg, 0.400 mmol, 2.00 equiv.) at 23 °C. MeCN (2.0 mL, c = 0.10 M) was added, and the resulting reaction mixture was stirred at 23 °C for 16 hours. Then, the solvent was concentrated under reduced pressure. The resulting residue was purified by column chromatography on silica gel eluting with pentane / EtOAc (100% pentane – 8:2, v/v) to afford the desired product as a colorless solid (27.0 mg, 0.094 mmol, 47%). The constitution of the product was confirmed from HMBC data.

R$_f$ = 0.33 (pentane/EtOAc, 9:1, (v/v)).

NMR Spectroscopy:

^{1}**H NMR** (500 MHz, $CDCl_3$, 23 ºC) δ 8.06 (dd, *J* = 2.4, 1.0 Hz, 1H), 7.65 (dt, *J* = 8.5, 2.1 Hz, 1H), 7.40 (td, *J* = 8.7, 6.3 Hz, 1H) , 7.11 (d, *J* = 9.0 Hz, 1H), 6.98 – 6.88 (m, 2H), 5.18 (d, *J* = 2.1 Hz, 1H), 4.94 (d, *J* = 2.0 Hz, 1H), 4.79 (s, 2H).

^{13}**C{^{1}H} NMR** (151 MHz, $CDCl_3$, 23 ºC) δ 165.2, 162.6 (dd, *J* = 249.8, 11.9 Hz), 159.8 (dd, *J* = 250.6, 11.8 Hz), 156.2, 150.8, 135.6 (d, *J* = 3.8 Hz), 134.2 (d, *J* = 2.4 Hz), 131.3 (dd, *J* = 9.5, 4.7 Hz), 130.0 (d, *J* = 1.3 Hz), 123.5 (dd, *J* = 13.4, 4.0 Hz), 121.1, 119.0, 111.9 (dd, *J* = 21.2, 3.8 Hz), 106.1, 104.7 (t, *J* = 26.7 Hz), 71.7.

^{19}**F NMR** (471 MHz, $CDCl_3$, 23 ºC) δ –110.54 (p, *J* = 7.8 Hz), –113.56 (q, *J* = 8.9 Hz).

HRMS-EI(m/z) calc'd for $C_{16}H_{10}O_3F_2$ $[M]^+$, 288.0592; found, 288.0597; deviation: –1.5 ppm.

(7,9-Di-*tert*-butyl)benzodioxepinone derivative 9

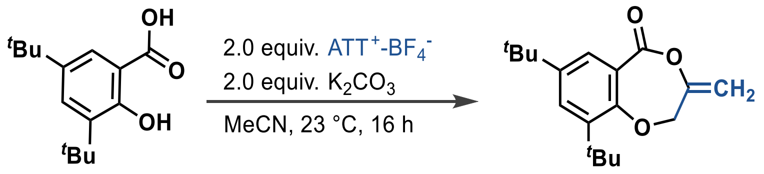

9, 65%

Under air, a 20 mL borosilicate vial was charged with (3,5-di-*tert*-butyl)salicylic acid (50.1 mg, 0.200 mmol, 1.00 equiv.), allenyl thianthrenium tetrafluoroborate salt **1-BF$_4$** (137 mg, 0.400 mmol, 2.00 equiv.), and potassium carbonate (55.3 mg, 0.400 mmol, 2.00 equiv.) at 23 °C. MeCN (2.0 mL, c = 0.10 M) was added,

and the resulting reaction mixture was stirred at 23 °C for 16 hours. Then, the solvent was concentrated under reduced pressure. The resulting residue was purified by column chromatography on silica gel eluting with pentane / EtOAc (100% pentane – 9:1, v/v) to afford the desired product as a colorless solid (37.5 mg, 0.130 mmol, 65%). The constitution of the product was confirmed from HMBC data.

R*f* = 0.47 (pentane/EtOAc, 9:1, (v/v)).

NMR Spectroscopy:

^{1}H NMR (500 MHz, $CDCl_3$, 23 ºC) δ 7.57 (d, *J* = 2.5 Hz, 1H), 7.55 (d, *J* = 2.6 Hz, 1H), 5.08 (d, *J* = 2.1 Hz, 1H), 4.82 (d, *J* = 2.1 Hz, 1H), 4.79 (s, 2H), 1.39 (s, 9H), 1.31 (s, 9H).

^{13}C{^{1}H} NMR (126 MHz, $CDCl_3$, 23 ºC) δ 167.6, 152.4, 151.5, 146.6, 141.8, 129.5, 127.0, 124.0, 105.8, 73.3, 35.5, 34.8, 31.4, 30.7.

HRMS-EI(m/z) calc'd for $C_{18}H_{24}O_3$ $[M]^+$, 288.1720; found, 288.1722; deviation: –0.7 ppm.

Benzodioxine derivative 10

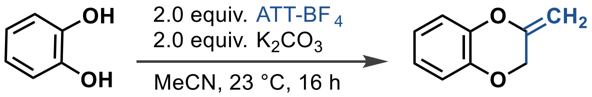

10, 78%

Under air, a 20 mL borosilicate vial was charged with catechol (22.0 mg, 0.200 mmol, 1.00 equiv.), allenyl thianthrenium tetrafluoroborate salt **1-BF$_4$** (137 mg, 0.400 mmol, 2.00 equiv.) and potassium carbonate (55.3 mg, 0.400 mmol, 2.00 equiv.) at 23 °C. MeCN (2.0 mL, c = 0.10 M) was added, and the resulting reaction mixture was stirred at 23 °C for 16 hours. Then, the solvent was concentrated under reduced pressure. The resulting residue was purified by column chromatography on silica gel eluting with pentane to afford the desired product as a colorless oil (23.0 mg, 0.155 mmol, 78%).

R*f* = 0.41 (pentane/EtOAc, 20:1, (v/v)).

NMR Spectroscopy:

^{1}H NMR (500 MHz, $CDCl_3$, 23 ºC) δ 7.00 – 6.86 (m, 4H), 4.75 (d, *J* = 1.9 Hz, 1H), 4.51 (s, 2H), 4.36 (d, *J* = 1.9 Hz, 1H).

^{13}C{^{1}H} NMR (126 MHz, $CDCl_3$, 23 ºC) δ 150.3, 143.9, 142.8, 122.4, 122.3, 117.4, 116.6, 91.4, 64.8.

HRMS-EI(m/z) calc'd for $C_9H_8O_2$ $[M]^+$, 148.0519; found, 148.0518; deviation: +0.2 ppm.

Pseudoephedrine derivative 11

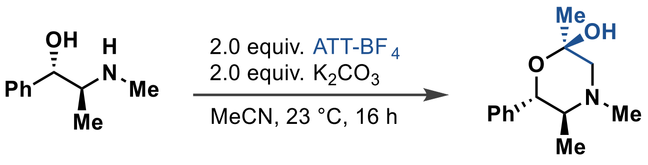

11, 75%

Under air, a 20 mL borosilicate vial was charged with D-(+)-pseudoephedrine (33.0 mg, 0.200 mmol, 1.00 equiv.), allenyl thianthrenium tetrafluoroborate salt **1-BF₄** (137 mg, 0.400 mmol, 2.00 equiv.) and potassium carbonate (55.3 mg, 0.400 mmol, 2.00 equiv.) at 23 °C. MeCN (2.0 mL, c = 0.10 M) was added, and the resulting reaction mixture was stirred at 23 °C for 16 hours. Then, the solvent was concentrated under reduced pressure. The resulting residue was purified by column chromatography on silica gel eluting with DCM / MeOH (100% DCM – 20:1, v/v) to afford the desired product as a colorless solid (33.0 mg, 0.149 mmol, 75%). The constitution and configuration of the product was confirmed by HMBC and NOESY data, respectively.

R_f = 0.56 (DCM/MeOH, 9:1, (v/v)).

NMR Spectroscopy:

^{1}H NMR (600 MHz, $CDCl_3$, 23 ºC) δ 7.35 – 7.31 (m, 4H), 7.30 – 7.26 (m, 1H), 4.50 (d, *J* = 9.5 Hz, 1H), 4.45 (bs, 1H), 2.77 (d, *J* = 11.1 Hz, 1H), 2.47 (d, *J* = 11.1 Hz, 1H), 2.32 (s, 3H), 2.13 (dd, *J* = 9.6, 6.2 Hz, 1H), 1.42 (s, 3H), 0.87 (d, *J* = 6.2 Hz, 3H).

^{13}C{^{1}H} NMR (151 MHz, $CDCl_3$, 23 ºC) δ 139.8, 128.4, 128.4, 128.3, 93.8, 78.6, 64.8, 63.6, 42.8, 25.6, 14.8.

HRMS-ESIpos(m/z) calc'd for $C_{13}H_{20}O_2N_1$ $[M+H]^+$, 222.1488; found, 222.1490; deviation: –0.6 ppm.

Possible mechanism:

It is conceivable that due to intermolecular hydrogen bonding between the OH and NHMe group of D(+)-pseudoephedrine molecules,[4] the nucleophilicity at the oxygen center exceeds that of the nitrogen, possibly by deprotonation in presence of K_2CO_3. Selectivity of N versus O alkylation in aliphatic amino alcohols is known to be influenced by the identity of the base[5], e.g. in some cases using K_2CO_3 favors O-substitution.[6]

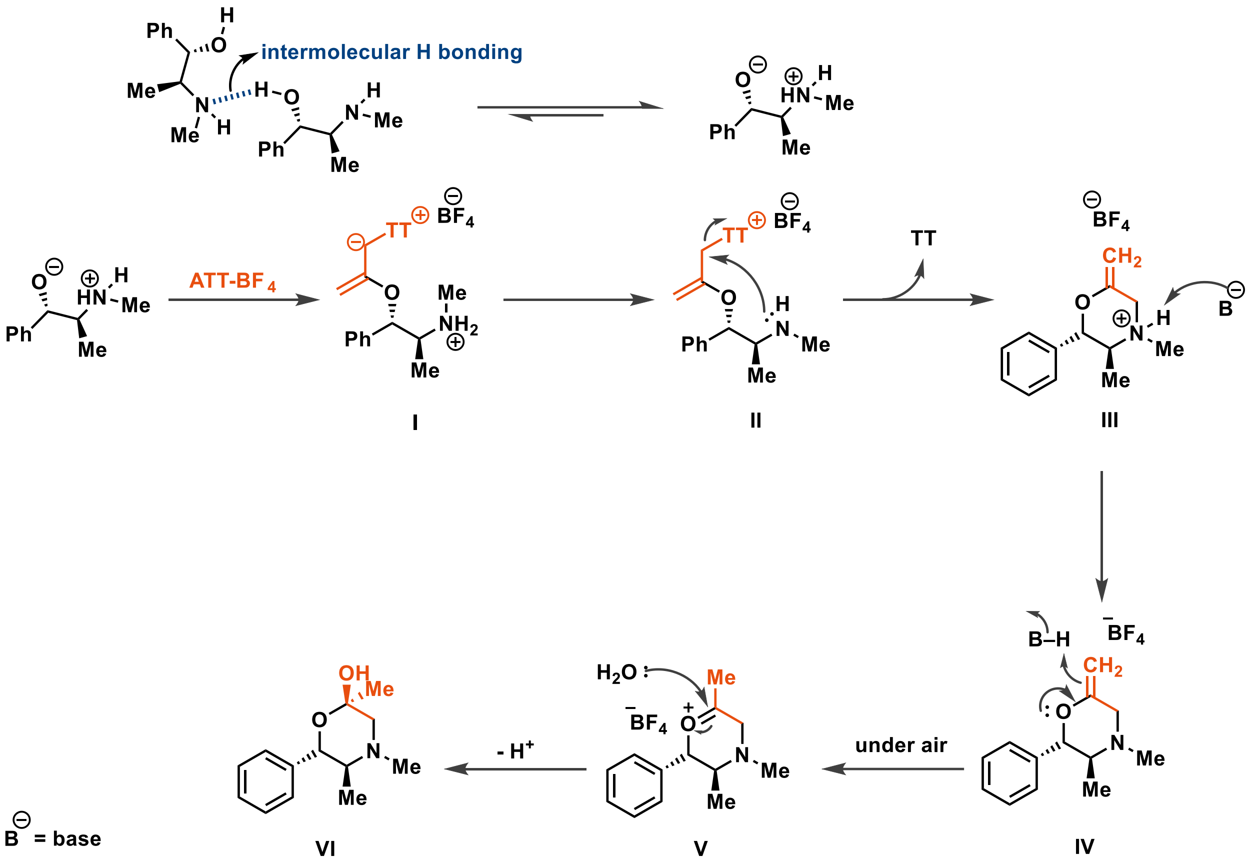

Figure S5: Possible mechanistic pathway to obtain product **11** from D(+)-pseudoephedrine.

Suzuki coupling reactions with allenyl thianthrenium salt 1

General procedure for Suzuki coupling reaction with allenyl thianthrenium salt 1

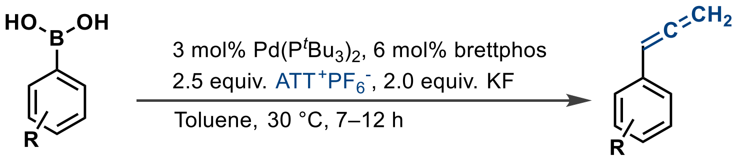

Under air, a 20 mL borosilicate vial (vial 1) was charged with substituted aryl boronic acids (0.500 mmol, 1.00 equiv.), brettphos (16.1 mg, 0.030 mmol, 0.060 equiv.) and potassium fluoride (58.1 mg, 1.00 mmol, 2.00 equiv.) at 23 °C. In another 4 mL borosilicate vial (vial 2), allenyl thianthrenium hexafluorophosphate **1** (0.500 g, 1.25 mmol, 2.50 equiv.) was added at 23 °C. Both vials were sealed with Teflon-lined screw caps and then they were transferred to N_2 filled glovebox. $Pd(P^tBu_3)_2$ (7.70 mg, 0.015 mmol, 0.030 equiv.) was added to vial 1 followed by the addition of dry toluene (10.0 mL, c = 0.050 M). The resulting reaction mixture was stirred (500 rpm) at 23 °C for a minute. Then, allenyl thianthrenium hexafluorophosphate salt **1** from vial 2 was added at once to vial 1 and it was sealed using a Teflon-lined screw cap. The sealed vial 1 was transferred out of the glovebox, placed on a heating block, and stirred (500 rpm) at 30 °C for 7 – 12 hours. Then, the solvent was concentrated under reduced pressure. The resulting residue was purified by column chromatography on silica gel eluting with pentane. A second purification by column chromatography on silver-

impregnated silica gel was carried out in cases where the hydro-defunctionalized product could not be separated from the desired product during the first purification.

Allenyl-4-phenylbenzene (12)

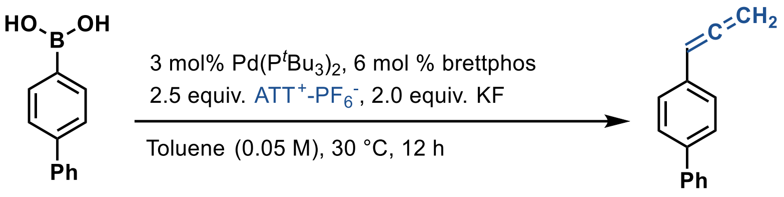

12, 52%

Under air, a 20 mL borosilicate vial (vial 1) was charged with 4-biphenyl boronic acid (99.0 mg, 0.500 mmol, 1.00 equiv.), brettphos (16.1 mg, 0.030 mmol, 0.060 equiv.) and potassium fluoride (58.1 mg, 1.00 mmol, 2.00 equiv.) at 23 °C. In another 4 mL borosilicate vial (vial 2), allenyl thianthrenium hexafluorophosphate **1** (0.500 g, 1.25 mmol, 2.50 equiv.) was added at 23 °C. Both vials were sealed with Teflon-lined screw caps and then they were transferred to a N_2 filled glovebox. $Pd(P^tBu_3)_2$ (7.70 mg, 0.015 mmol, 0.030 equiv.) was added to vial 1 followed by the addition of dry toluene (10.0 mL, c = 0.050 M). The resulting reaction mixture was stirred (500 rpm) at 23 °C for a minute. Then, allenyl thianthrenium hexafluorophosphate salt **1** from vial 2 was added at once to vial 1 and it was sealed using a Teflon-lined screw cap. The sealed vial 1 was transferred out of the glovebox, placed on a heating block, and stirred (500 rpm) at 30 °C for 12 hours. Then, the solvent was concentrated under reduced pressure. The resulting residue was purified by column chromatography on silica gel eluting with pentane. A second purification by column chromatography on silver-impregnated silica gel with pentane yielded the desired product as a colorless solid (50.0 mg, 0.260 mmol, 52%).

[Note: The product decomposed when stored for more than a week in a closed vial under ambient atmosphere]

R*f* = 0.33 (100% pentane).

NMR Spectroscopy:

^{1}H NMR (500 MHz, $CDCl_3$, 23 ºC) δ 7.60 (d, *J* = 7.1 Hz, 2H), 7.55 (d, *J* = 8.3 Hz, 2H), 7.44 (t, *J* = 7.7 Hz, 2H), 7.39 – 7.32 (m, 3H), 6.22 (t, *J* = 6.8 Hz, 1H), 5.19 (d, *J* = 6.8 Hz, 2H).

^{13}C{^{1}H} NMR (126 MHz, $CDCl_3$, 23 ºC) δ 210.2, 141.0, 139.9, 133.1, 128.9, 127.5, 127.4, 127.2, 127.1, 93.8, 79.0.

HRMS-EI(m/z) calc'd for $C_{15}H_{12}$ $[M]^+$, 192.0934; found,192.0934; deviation: –0.1 ppm.

Allenyl-(2,4,6-trimethyl)benzene (13)

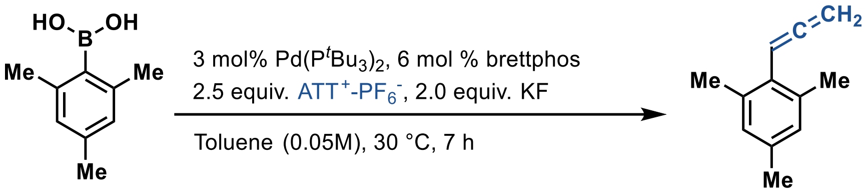

13, 42%

Under air, a 20 mL borosilicate vial (vial 1) was charged with (2,4,6-trimethyl)phenyl boronic acid 97% (84.5 mg, 0.500 mmol, 1.00 equiv.), brettphos (16.1 mg, 0.030 mmol, 0.060 equiv.) and potassium fluoride (58.1 mg, 1.00 mmol, 2.00 equiv.) at 23 °C. In another 4 mL borosilicate vial (vial 2), allenyl thianthrenium hexafluorophosphate salt **1** (0.500 g, 1.25 mmol, 2.50 equiv.) was added at 23 °C. Both vials were sealed with Teflon-lined screw caps and were transferred to N_2 filled glovebox. Pd(P^{t}Bu$_3$)$_2$ (7.70 mg, 0.015 mmol, 0.030 equiv.) was added to vial 1 followed by the addition of dry toluene (10.0 mL, c = 0.050 M). The resulting reaction mixture was stirred (500 rpm) at 23 °C for a minute. Then, allenyl thianthrenium hexafluorophosphate **1** from vial 2 was added at once to vial 1 and was sealed using a Teflon-lined screw cap. The sealed vial 1 was transferred out of the glovebox, placed on a heating block, and stirred (500 rpm) at 30 °C for 7 hours. Then, the solvent was concentrated under reduced pressure. The resulting residue was purified by column chromatography on silica gel eluting with pentane to afford the desired product as a colorless liquid (33 mg, 0.209 mmol, 42%).

[*Note: The product decomposed when stored for more than a week in a closed vial under ambient atmosphere.]*

R*f* = 0.30 (100% pentane)

NMR Spectroscopy:

^{1}H NMR (500 MHz, $CDCl_3$, 23 ºC) δ 6.86 (s, 2H), 6.24 (t, *J* = 7.0 Hz, 1H), 4.91 (d, *J* = 7.0 Hz, 2H), 2.34 (s, 6H), 2.27 (s, 3H).

^{13}C{^{1}H} NMR (126 MHz, $CDCl_3$, 23 ºC) δ 210.5, 136.6, 136.4, 129.1, 128.3, 89.5, 75.9, 21.2, 21.0.

HRMS-EI(m/z) calc'd for $C_{12}H_{14}$ $[M]^+$, 158.1090; found, 158.1092; deviation: –1.5 ppm.

Allenyl-(4-*tert*-butyl)benzene (14)

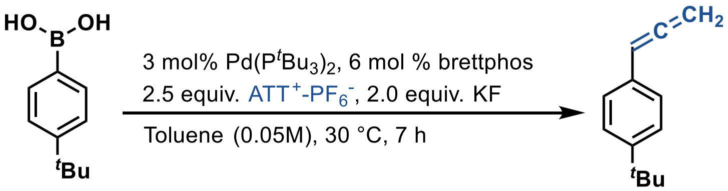

14, 53%

Under air, a 20 mL borosilicate vial (vial 1) was charged with (4-*tert*-butyl)phenyl boronic acid (89.0 mg, 0.500 mmol, 1.00 equiv.), brettphos (16.1 mg, 0.030 mmol, 0.060 equiv.) and potassium fluoride (58.1 mg, 1.00

mmol, 2.00 equiv.) at 23 °C. In another 4 mL borosilicate vial (vial 2), allenyl thianthrenium hexafluorophosphate salt **1** (0.500 g, 1.25 mmol, 2.50 equiv.) was added at 23 °C. Both vials were sealed with Teflon-lined screw caps and were transferred to N_2 filled glovebox. Pd(P^tBu_3)$_2$ (7.70 mg, 0.015 mmol, 0.030 equiv.) was added to vial 1 followed by the addition of dry toluene (10.0 mL, c = 0.050 M). The resulting reaction mixture was stirred (500 rpm) at 23 °C for a minute. Then, allenyl thianthrenium hexafluorophosphate **1** from vial 2 was added at once to vial 1 and was sealed using a Teflon-lined screw cap. The sealed vial 1 was transferred out of the glovebox, placed on a heating block, and stirred (500 rpm) at 30 °C for 7 hours. Then, the solvent was concentrated under reduced pressure. The resulting residue was purified by column chromatography on silica gel eluting with pentane to afford the desired product as a colorless liquid (46.0 mg, 0.267 mmol, 53%).

[Note: The product decomposed when stored for more than 24 hours in a closed vial under ambient atmosphere.]

R_f = 0.30 (100% pentane)

NMR Spectroscopy:

^{1}H NMR (500 MHz, $CDCl_3$, 23 ºC) δ 7.34 (d, *J* = 8.4 Hz, 2H), 7.24 (d, *J* = 8.4 Hz, 2H), 6.16 (t, *J* = 6.8 Hz, 1H), 5.13 (d, *J* = 6.8 Hz, 2H), 1.32 (s, 9H).

^{13}C{^{1}H} NMR (126 MHz, $CDCl_3$, 23 ºC) δ 209.9, 150.1, 131.1, 126.5, 125.7, 93.7, 78.7, 34.7, 31.5.

HRMS-EI(m/z) calc'd for $C_{13}H_{16}$ $[M]^+$, 172.1246; found, 172.1249; deviation: –1.7 ppm.

Reductive coupling reaction with allenyl thianthrenium salt 1

3-Allenyl-1-boc-azetidine (15)

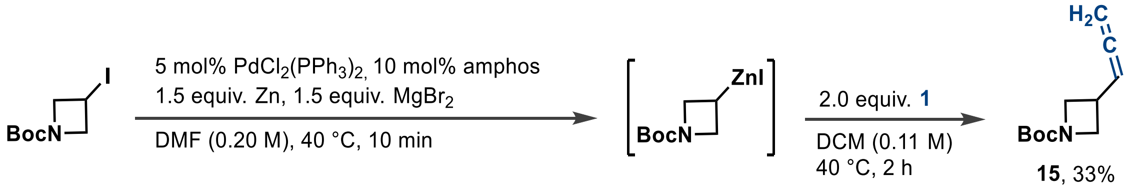

Following our reported procedure for reductive coupling with alkyl iodides and vinyl thianthrenium salt,[7] a 20 mL borosilicate vial (vial 1) was charged with amphos (13.2 mg, 0.050 mmol, 0.100 equiv.) and $PdCl_2(PPh_3)_2$ (17.5 mg, 0.025 mmol, 0.050 equiv.) under air at 23 °C. In another 4 mL borosilicate vial (vial 2), allenyl thianthrenium hexafluorophosphate **1** (0.400 g, 1.00 mmol, 2.00 equiv.) was added at 23 °C. Both vials were sealed with Teflon-lined screw caps and then they were transferred to a N_2 filled glovebox. Activated zinc powder 100-mesh 99.5% (49.0 mg, 0.750 mmol, 1.50 equiv.) (prepared by reported procedure[7]) and self-made anhydrous $MgBr_2$ (138 mg, 0.750 mmol, 1.50 equiv.) (prepared by reported procedure[7]) were added to vial 1 followed by the addition of dry DMF (3 mL, c = 0.2 M). Subsequently, 1-Boc-3-iodoazetidine (86.8 µL, 142 mg, 0.500 mmol, 1.00 equiv.) was added to vial 1 at 23 °C. Vial 1 was sealed with a vial adapter, removed from the glovebox, and placed in a heating block preheated to 40 °C where the reaction mixture was

stirred rigorously (850 rpm) for 10 minutes at 40 °C. In that time, a color change from yellow to dark green/brown was observed. In the meantime, dry DCM (9 mL, c = 0.1 M) was added to vial 2 containing allenyl thianthrenium hexafluorophosphate salt **1** in a N_2 filled glovebox. The solution of allenyl thianthrenium hexafluorophosphate **1** in DCM from vial 2 was drawn into a syringe and transferred out of the glovebox. After stirring the reaction mixture in vial 1 for 10 minutes, the solution of allenyl thianthrenium hexafluorophosphate salt **1** was added dropwise to vial 1 under an argon atmosphere over 9 minutes at a flowrate of approximately 1 mL per minute. Once all the contents of vial 2 were added, vial 1 was sealed and the reaction mixture was stirred (850 rpm) at 40 °C for 2 h. The reaction mixture was cooled to 23 °C, concentrated under reduced pressure, poured into a separatory funnel, and subsequently diluted with ethyl acetate (40 mL). The organic layer was washed with water (1 × 20 mL), then the aqueous layer was extracted with ethyl acetate (2 × 20 mL). The combined organic layer was washed with brine (1 × 60 mL). The organic layer was dried over $MgSO_4$, filtered and concentrated under reduced pressure. The resulting residue was purified by column chromatography on silica gel eluting with a solvent mixture of hexanes / EtOAc (10:0 gradient to 8:2 (v/v)). A second purification by column chromatography on silver-impregnated silica gel with a solvent mixture of hexanes / EtOAc (100% hexanes – 8:2 (v/v)) yielded the desired product as a colorless oil (32 mg, 0.164 mmol, 33%).

R*f* = 0.48 (pentane/EtOAc, 9:1, (v/v)).

NMR Spectroscopy:

^{1}H NMR (500 MHz, $CDCl_3$, 23 ºC) δ 5.29 (q, *J* = 6.7 Hz, 1H), 4.83 (dd, *J* = 6.6, 2.7 Hz, 2H), 4.07 (t, *J* = 8.4 Hz, 2H), 3.75 (dd, *J* = 8.5, 5.9 Hz, 2H), 3.16 – 3.09 (m, 1H), 1.43 (s, 9H).

^{13}C{^{1}H} NMR (126 MHz, $CDCl_3$, 23 ºC) δ 208.1,156.5, 92.0, 79.5, 77.4, 55.1, 28.5, 27.3.

HRMS-ESIpos(m/z) calc'd for $C_{11}H_{17}NO_2Na$ $[M+Na]^+$, 218.1152; found, 218.1149; deviation: +1.0 ppm.

3-Aminophenol-derived O-homoacylation product 16

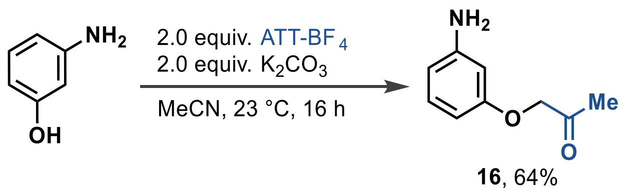

16, 64%

Under air, a 20 mL borosilicate vial was charged with 3-aminophenol (21.8 mg, 0.200 mmol, 1.00 equiv.), allenyl thianthrenium tetrafluoroborate salt **1-BF$_4$** (137 mg, 0.400 mmol, 2.00 equiv.) and potassium carbonate (55.3 mg, 0.400 mmol, 2.00 equiv.) at 23 °C. MeCN (2.0 mL, c = 0.10 M) was added, and the resulting reaction mixture was stirred at 23 °C for 16 hours. Then, the solvent was concentrated under reduced pressure. The resulting residue was purified by column chromatography on silica gel eluting with DCM / MeOH (100% DCM – 20:1, v/v) to afford the desired product as a dark yellow oil (21.0 mg, 0.127 mmol, 64%).

R*f* = 0.68 (DCM/MeOH, 9:1, (v/v)).

NMR Spectroscopy:

^{1}H NMR (500 MHz, $CDCl_3$, 23 ºC) δ 7.06 (t, *J* = 8.0 Hz, 1H), 6.34 (dd, *J* = 8.0, 3.0 Hz, 1H), 6.27 – 6.21 (m, 2H), 4.48 (s, 2H), 3.69 (s, 2H), 2.26 (s, 3H).

$^{13}C\{^1H\}$ NMR (126 MHz, $CDCl_3$, 23 ºC) δ 206.6, 159.1, 148.1, 130.5, 109.0, 104.3, 101.8, 73.2, 26.8.

HRMS-EI(m/z) calc'd for $C_9H_{11}NO_2$ $[M]^+$, 165.0784; found, 165.0786; deviation: –1.3 ppm.

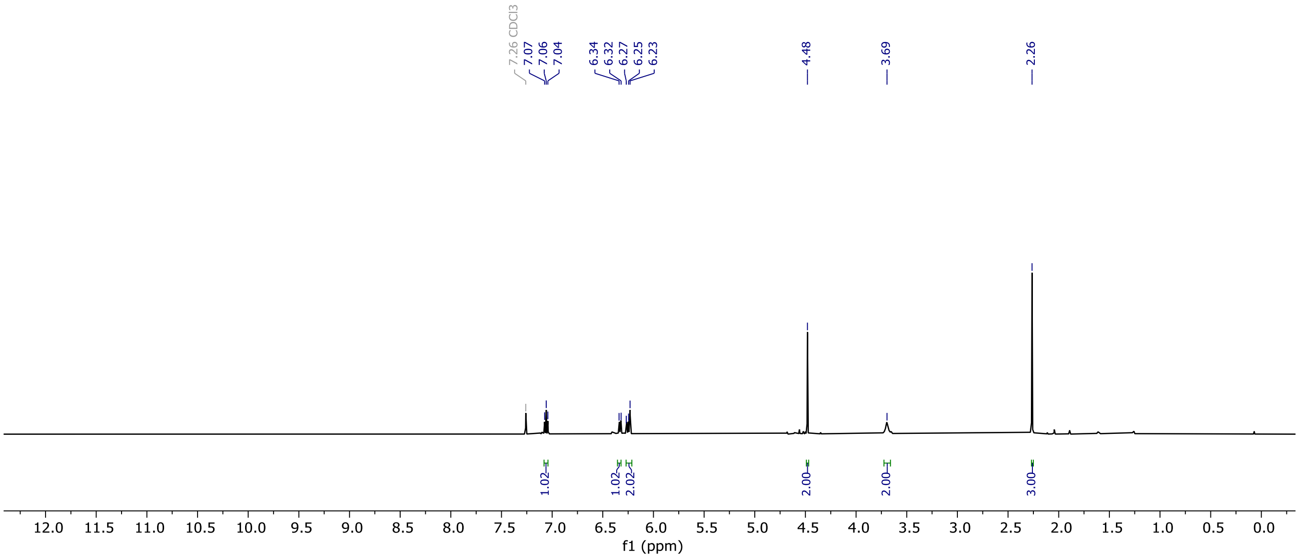

Figure S6. ^{1}H NMR spectra of **16** in $CDCl_3$, 500 MHz, 23 ºC

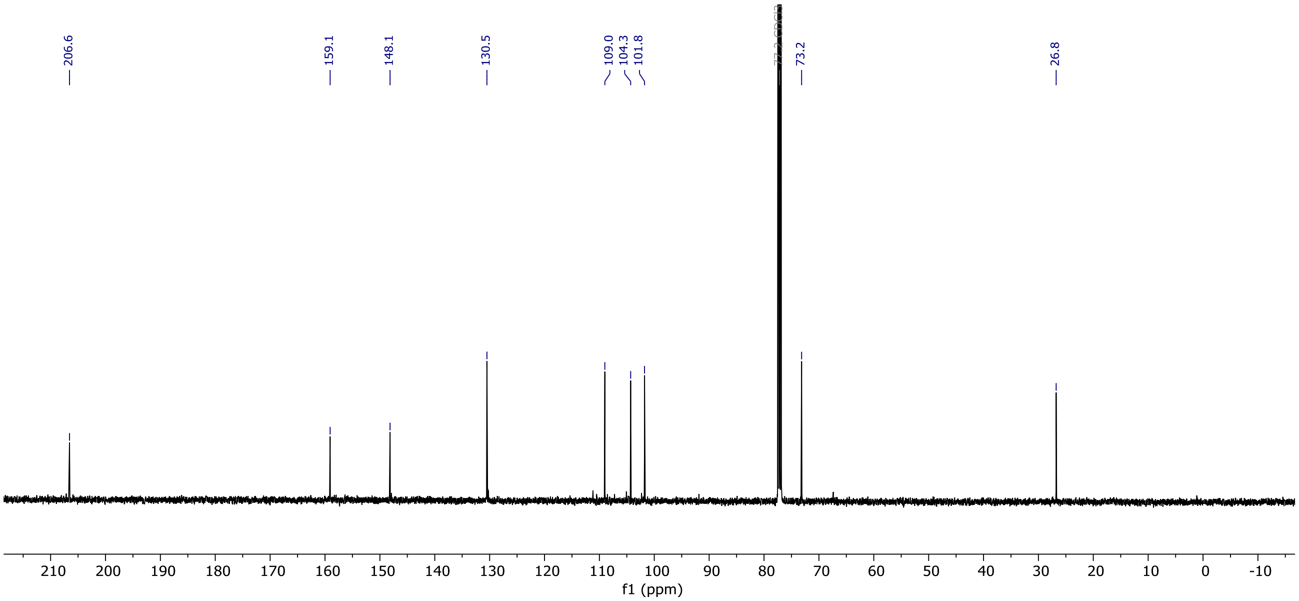

Figure S7. ^{13}C NMR spectra of **16** in $CDCl_3$, 500 MHz, 23 ºC

Reaction optimization for Suzuki coupling

General procedure for Suzuki coupling with allenyl thianthrenium salt 1

Under air, a 4 mL borosilicate vial (vial 1) was charged with 4-biphenyl boronic acid (9.90 mg, 0.050 mmol, 1.00 equiv.), catalyst (if moisture insensitive), ligand, and base at 23 °C. In another 1 mL glass vial (vial 2), allenyl thianthrenium hexafluorophosphate salt **1** (50.0 mg, 0.125 mmol, 2.50 equiv.) was added at 23 °C. Both vials were sealed with Teflon-lined screw caps and then they were transferred to N_2 filled glovebox. Moisture-sensitive catalysts (if any), were added to vial 1 followed by the addition of dry toluene (1.0 mL, c = 0.05 M). The resulting reaction mixture was stirred (500 rpm) at 23 °C for a minute. Then, allenyl thianthrenium hexafluorophosphate salt **1** from vial 2 was added at once to vial 1 and it was sealed using a Teflon-lined screw cap. The sealed vial 1 was transferred out of the glovebox, placed on a heating block, and stirred (500 rpm) at 30 °C for 7–12 hours. Then, the solvent was concentrated under reduced pressure and diluted with 0.7 mL $CDCl_3$, followed by the addition of mesitylene as an internal standard (7.0 μL, 0.05 mmol, 1.00 equiv.). The resulting mixture was passed through celite and submitted for ^{1}H NMR measurement to check the NMR yield. The ^{1}H NMR resonances of the allenyl protons of the product at 6.3 ppm and 5.3 ppm were integrated relative to the ^{1}H NMR resonances of the aromatic protons of mesitylene at 6.9 ppm. N.D = not determined.

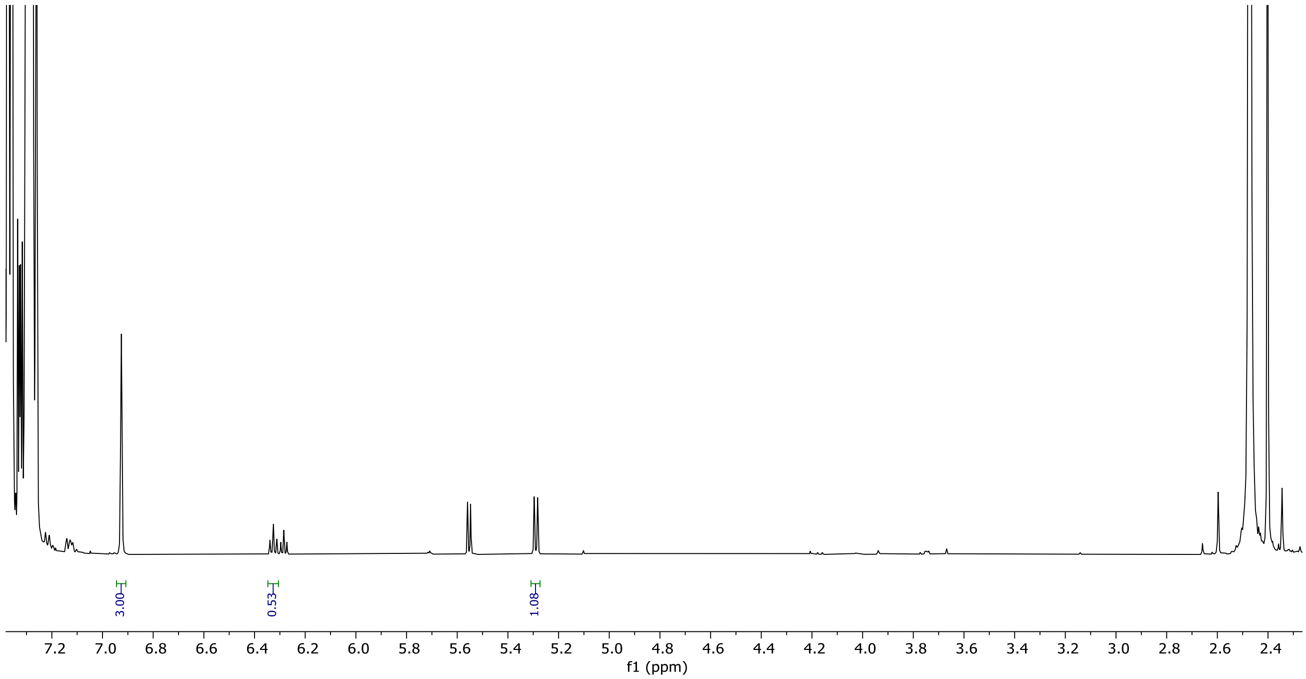

Figure S8: Determination of the yield of **12** via ^{1}H NMR analysis, $CDCl_3$, 500 MHz, 23 ºC

Table S1: Optimization of yield as a function of catalyst

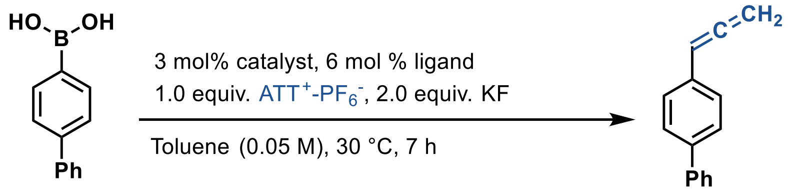

12

Entry	Catalyst	Yield
1	$Pd(dba)_2 + PCy_3$	20%
2	$Pd(P^tBu_3)_2$+ PCy_3	24%
3	$Pd(P^tBu_3)_2 + PCy_3$, 10 °C	5%
4	$Pd(P^tBu_3)_2 + PCy_3$, 50 °C	2%
5	$PdCl_2(PCy_3)_2$	N.D.
6	$Pd(dba)_2 + HP^tBu_3BF_4$, 18 h at rt	33%
7	$Pd(dba)_2$ + XPhos	4%
**8**	**$Pd(P^tBu_3)_2$ + brettphos**	**37%**
9	$Pd(OAc)_2 + PCy_3$	N.D.
10	Brettphos Pd G4	3%
11	No catalyst	N.D.
12	$Pd(P^tBu_3)_2$	17%
13	$NiCl_2(PPh_3)_2$	N.D.
14	$Pd(dba)_2$ + P(*o*-tol)$_3$	4%

Table S2: Optimization of yield as a function of base with different catalysts

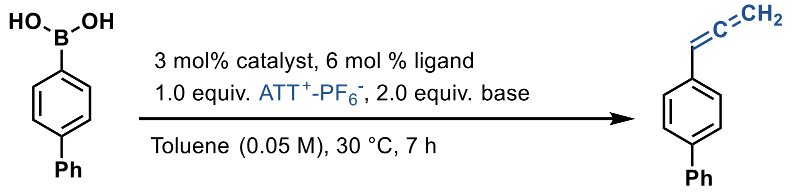

12

Entry	Catalyst	Base	Yield
**1**	**$Pd(P^tBu_3)_2$ + brettphos**	**KF**	**37%**
2	$Pd(P^tBu_3)_2$, 70 °C	KF	<2%
3	$Pd(dba)_2 + PCy_3$	KF	22%
4	$Pd(dba)_2 + PCy_3$	NaF	N.D.
5	$Pd(P^tBu_3)_2$ + brettphos	CsF	N.D.
6	$Pd(P^tBu_3)_2$ + brettphos	NaF	N.D.
7	$Pd(dba)_2 + PCy_3$	CsF	N.D.
8	$Pd(dba)_2 + PCy_3$	Li^tBuO	5%
9	$Pd(dba)_2 + PCy_3$	K^tBuO	N.D.
10	$Pd(dba)_2 + PCy_3$	NaOH	N.D.
11	$Pd(P^tBu_3)_2 + PCy_3$	NaOH	12%
12	$Pd(dba)_2 + PCy_3$	TMAF	5%
13	$Pd(P^tBu_3)_2$ + brettphos	no base	<2%
14	$Pd(P^tBu_3)_2 + PCy_3$	no base	4%
15	$Pd(dba)_2$ + P(*o*-tol)$_3$	Li^tBuO	4%

Table S3: Optimization of yield as a function of solvent

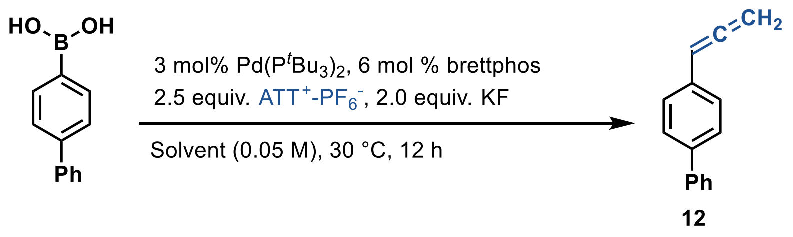

Entry	Solvent	Yield
1	DMF	N.D.
2	THF	N.D.
3	DCM	N.D.
4	MeCN	23%

**5**	**Toluene**	**53%**
6	Benzene	30%
**7**	**benzene-d_6**	**58%**
8	Dioxane	3%

Table S4: Optimization of yield as a function of time and equivalents of 1

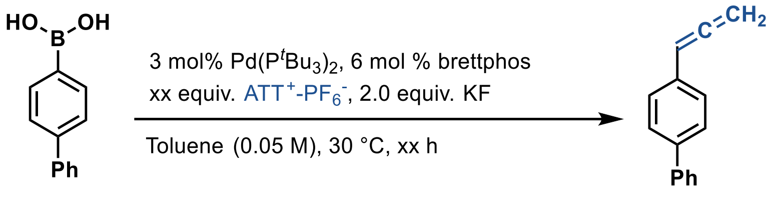

12

Entry	Reaction conditions	Yield
**1**	**2.5 equiv., 12 h**	**53%**
2	2.5 equiv., 7 h	42%
3	2.5 equiv., 20 h	21%
4	1.0 equiv., 7 h	37%
5	2.0 equiv., 12 h	41%
6	3.0 equiv., 12 h	50%
7	2.5 equiv. **1-BF$_4$**, 12 h	30%

Table S5: Optimization of yield as a function of temperature

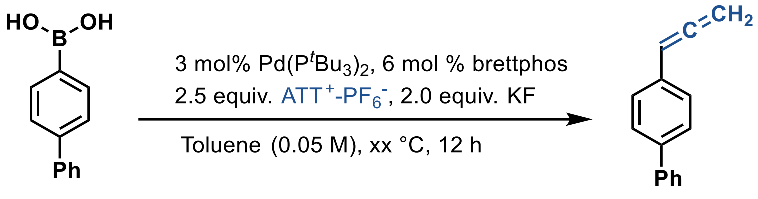

12

Entry	temperature	Yield
**1**	**30 °C**	**53%**
2	22 °C	47%
3	50 °C	22%

Unsuccessful substrates for Suzuki coupling reaction with allenyl thianthrenium salt 1

The following aryl boronic acids with a variety of different functional groups were used to study the reactivity of allenyl thianthrenium hexafluorophosphate **1**. The reactions produced the desired products in less than or equal to 22% yield. The main side product in every case was the hydro-defunctionalized product as identified from ^{1}H NMR and GC-MS data of the crude samples.

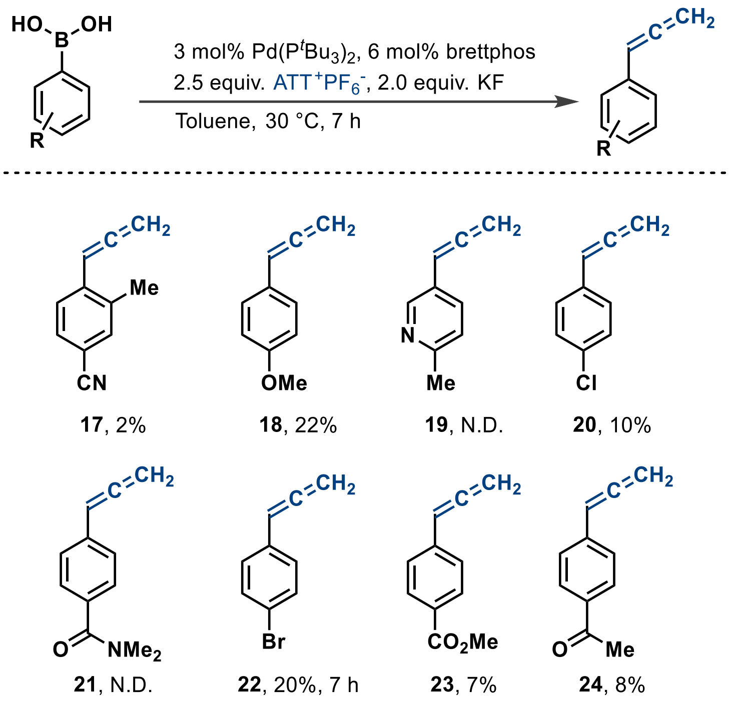

Figure S9. List of unsuccessful substrates for Suzuki coupling with **1**. The yields were measured from the ^{1}H NMR spectra of the crude samples using mesitylene as an internal standard. N.D.= not determined.

Reaction with aryl boronic acid S-25

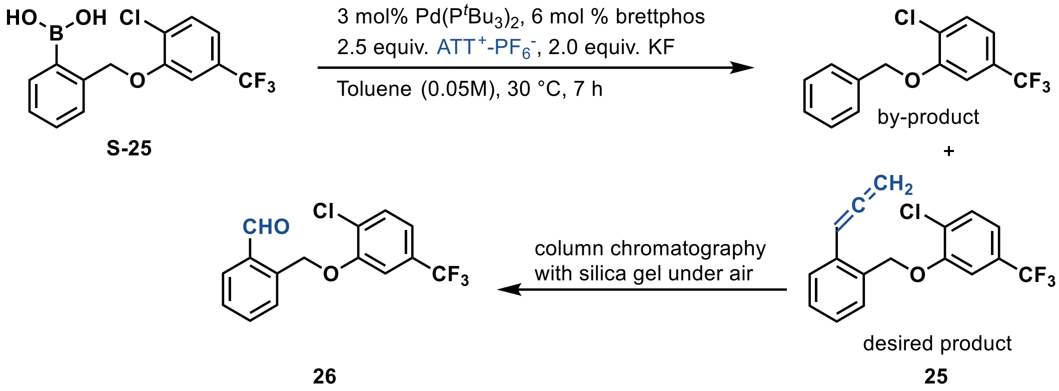

Under air, a 20 mL borosilicate vial (vial 1) was charged with **S-25** of 95% purity (174 mg, 0.500 mmol, 1.00 equiv.), brettphos (16.1 mg, 0.030 mmol, 0.060 equiv.) and potassium fluoride (58.1 mg, 1.00 mmol, 2.00 equiv.) at 23 °C. In another 4 mL borosilicate vial (vial 2), allenyl thianthrenium hexafluorophosphate salt **1** (0.500 g, 1.25 mmol, 2.50 equiv.) was added at 23 °C. Both vials were sealed with Teflon-lined screw caps

and were transferred to a N_2 filled glovebox. Pd(P^{t}Bu$_3$)$_2$ (7.70 mg, 0.015 mmol, 0.030 equiv.) was added to vial 1 followed by the addition of dry toluene (10.0 mL, c = 0.050 M). The resulting reaction mixture was stirred (500 rpm) at 23 °C for a minute. Then, allenyl thianthrenium hexafluorophosphate **1** from vial 2 was added at once to vial 1 and was sealed using a Teflon-lined screw cap. The sealed vial 1 was transferred out of the glovebox, placed on a heating block, and stirred (500 rpm) at 30 °C for 7 hours. Then, the solvent was concentrated under reduced pressure. The resulting residue was purified by column chromatography on silica gel eluting with pentane.

The reaction with **S-25** yielded 60% of the desired product, as determined by NMR. However, the desired product was found to decompose after column purification. When purification by column chromatography with silica gel was performed, both the desired product and the hydro-defunctionalized products were obtained together. When a second column purification was performed using silver-impregnated silica gel, most of the allenyl product **25** was found to react to the corresponding aldehyde **26**. Thus, the desired product **25** was always obtained as a mixture with the hydro-defunctionalized product. The ^{1}H NMR of a fraction of isolated product **26** is shown below. Here, it is to be noted that the decomposition is possibly taking place either due to the air-sensitive nature of the final product or its unstable nature in solution with $CDCl_3$. Similar decompositions were observed for compounds **12**, **13,** and **14** when stored for a prolonged period under an ambient atmosphere.

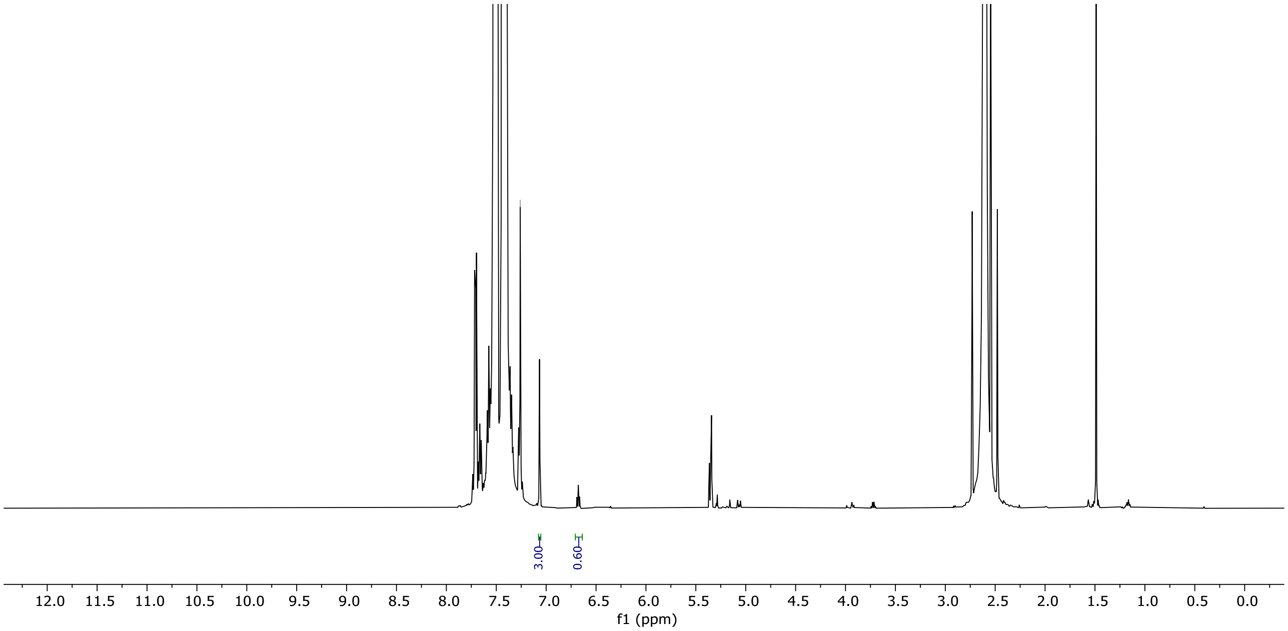

Figure S10. ^{1}H NMR spectrum of a crude sample of compound **25** (0.05 mmol scale) in $CDCl_3$, 500 MHz, 23 ºC using mesitylene as an internal standard.

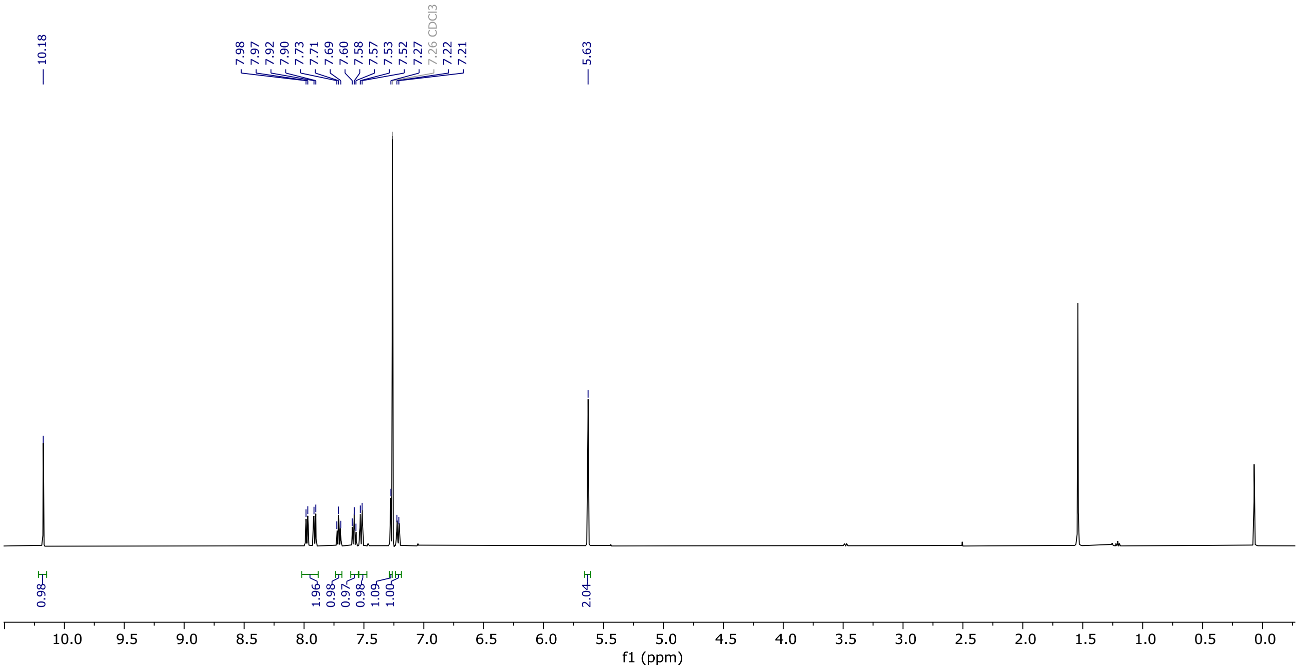

Figure S11. ^{1}H NMR spectrum of compound **26** in $CDCl_3$, 500 MHz, 23 ºC.

Unsuccessful substrates for reductive coupling reaction with allenyl thianthrenium salt 1

General procedure for reductive coupling with 1

Following our reported procedure for reductive coupling with alkyl iodide and vinyl thianthrenium salt,[7] a 4 mL borosilicate vial (vial 1) was charged with amphos (1.30 mg, 0.005 mmol, 0.100 equiv.) and $PdCl_2(PPh_3)_2$ (1.80 mg, 0.002 mmol, 0.050 equiv.) under air at 23 °C. In another 4 mL borosilicate vial (vial 2), allenyl thianthrenium hexafluorophosphate salt **1** (40.0 mg, 0.100 mmol, 2.00 equiv.) was added at 23 °C. Both vials were sealed with Teflon-lined screw caps and then they were transferred to a N_2 filled glovebox. Activated zinc powder 100-mesh 99.5% (4.90 mg, 0.075 mmol, 1.50 equiv.) (prepared by reported procedure[7]) and self-made anhydrous $MgBr_2$ (13.8 mg, 0.075 mmol, 1.50 equiv.) (prepared by reported procedure[7]) were added to vial 1 followed by the addition of dry DMF (0.3 mL, c = 0.2 M). Subsequently, alkyl iodide (0.050 mmol, 1.00 equiv.) was added to vial 1 at 23 °C. Vial 1 was sealed with a vial adapter, removed from the glovebox, and placed in a heating block preheated to 40 °C where the reaction mixture was stirred rigorously (850 rpm) for 10 minutes at 40 °C. In that time, a color change from yellow to dark green/brown was observed. In the meantime, dry DCM (0.9 mL, c = 0.1 M) was added to vial 2 containing allenyl thianthrenium hexafluorophosphate salt **1** in a N_2 filled glovebox. The solution of allenyl thianthrenium hexafluorophosphate salt **1** in DCM from vial 2 was drawn into a syringe and transferred out of the glovebox. After stirring the reaction mixture in vial 1 for 10 minutes, the solution of allenyl thianthrenium hexafluorophosphate salt **1** was added dropwise to vial 1 under an argon atmosphere over 9 minutes at a flowrate of approximately 0.1 mL per minute. Once all the contents of vial 2 were added, vial 1 was sealed and the reaction mixture was stirred (850 rpm) at 40 °C for 2 h. The reaction mixture was cooled to 23 °C, concentrated under reduced

pressure and, diluted with $CDCl_3$ (1 mL), followed by the addition of mesitylene as an internal standard (7.0 μL, 0.05 mmol, 1.0 equiv.). Subsequently, water (1 mL) was added to the vial and shaken vigorously. The organic layer was separated, passed through a silica plug and submitted for ^{1}H NMR.

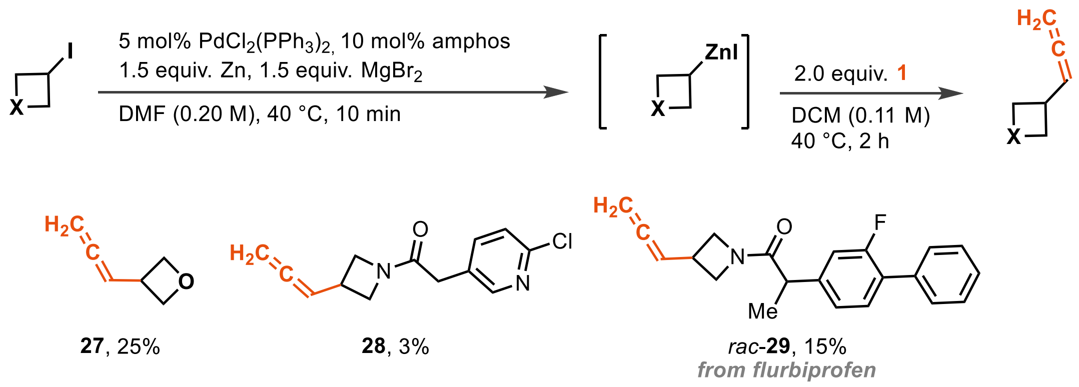

Figure S12. List of unsuccessful substrates for reductive coupling with **1**. The yields were measured from the ^{1}H NMR spectra of the crude samples using mesitylene as an internal standard.

To study the reductive coupling between alkyl iodides and allenyl thianthrenium hexafluorophosphate **1,** three additional substrates containing four-membered ring systems were investigated, namely compounds **27**, **28**, and **29** (Figure S12). All the three substrates yielded less than or equal to 25% of the desired product, giving the hydro-defunctionalized product as the major product. Additionally, in the case of larger rings like N-boc-4-iodopiperidine, the β-hydride elimination product is formed predominantly (Figure S13). When compared to the reductive coupling of vinyl thianthrenium salt[7] with alkyl iodide starting materials of compounds **15**, **28** and **29**, the allenyl thianthrenium salt seems to give a lower yield in all the cases. The unstable nature of the allenyl sulfonium salt or the allenyl products in presence of highly polar solvents like DMF (which is essential to carry out the zinc insertion in the first step[7]) is believed to be the reason behind such reduced reactivity.

Reductive coupling reaction of N-boc-4-iodopiperidine with 1

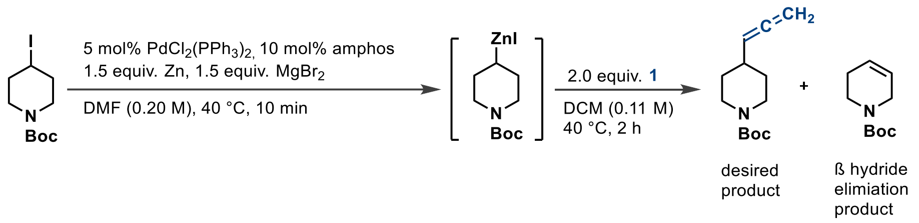

Following the general procedure as mentioned earlier, when the reaction was carried out with 0.05 mmol of N-boc-4-iodopiperidine we mainly obtained the corresponding ß-hydride elimination product in 22% yield, as determined by ^{1}H NMR.

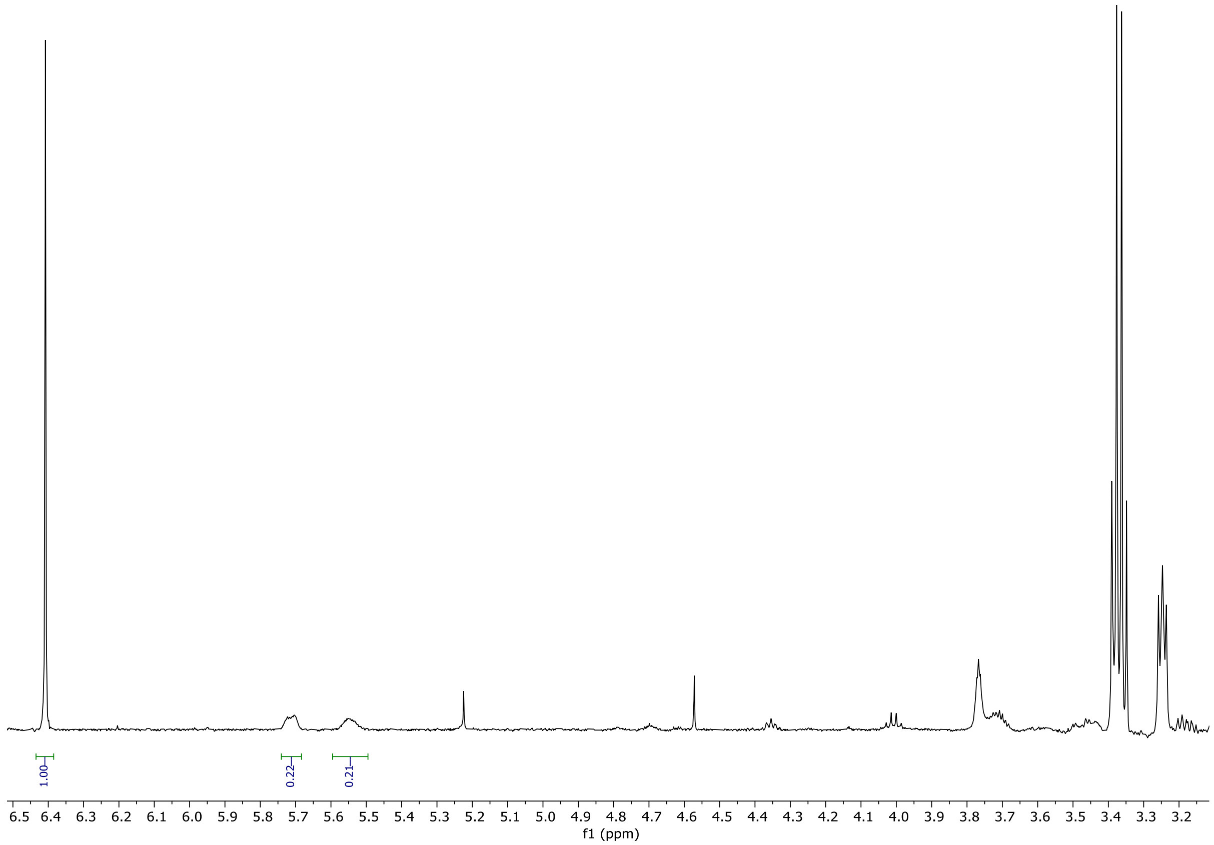

Figure S13. ^{1}H NMR spectra of a crude sample taken after the reaction of N-boc-4-iodopiperidine with **1**, $CDCl_3$, 500 MHz, 23 ºC using trichloroethylene (6.4 ppm) as an internal standard.

Reactivity study of propargyl thianthrenium salt 1a

The reactivity of the newly synthesized propargyl thianthrenium salt **1a** towards annulation and Suzuki coupling reaction was checked. In order to compare its reactivity with the allenyl isomer, the counter anions for both the reactions were the same as for the allenyl thianthrenium salt. The propargyl thianthrenium tetrafluoroborate and propargyl thianthrenium hexafluorophosphate were synthesized following the same anion exchange procedure used for the synthesis of **1-BF₄** from **1**.

Annulation reaction of catechol with propargyl thianthrenium tetrafluoroborate

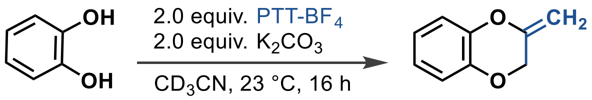

10

Under air, a 20 mL borosilicate vial was charged with catechol (5.50 mg, 0.050 mmol, 1.00 equiv.), propargyl thianthrenium tetrafluoroborate salt (34.2 mg, 0.100 mmol, 2.00 equiv.) and potassium carbonate (13.8 mg, 0.100 mmol, 2.00 equiv.) at 23 °C. Deuterated-MeCN (0.5 mL, c = 0.1 M) was added, and the resulting reaction mixture was stirred at 23 °C for 16 hours. Then, mesitylene (7.0 μL, 0.05 mmol, 1.00 equiv.).was

added as an internal standard. The resulting reaction mixture was filtered and submitted for ^{1}H NMR measurements. From the ^{1}H NMR measurement, it was confirmed that product **10** is formed with 50% yield.

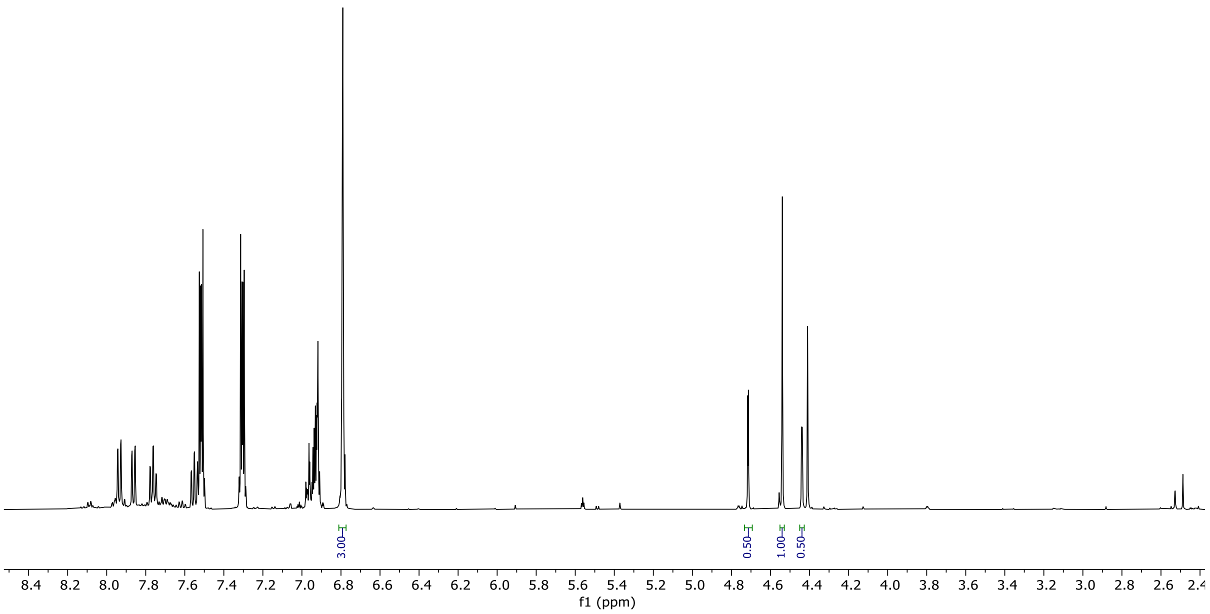

Figure S14. ^{1}H NMR spectra of the crude sample taken after the reaction of catechol with propargyl thianthrenium tetrafluoroborate in CD_3CN, 500 MHz, 23 ºC using mesitylene as an internal standard.

Suzuki coupling of 4-biphenyl boronic acid with propargyl thianthrenium hexafluorophosphate

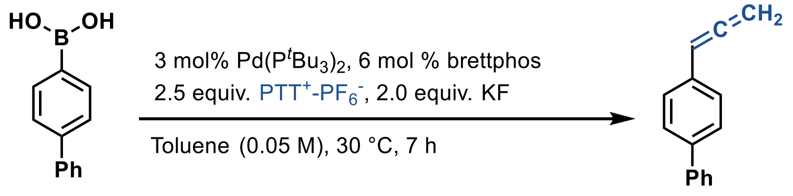

12

Under air, a 20 mL borosilicate vial (vial 1) was charged with 4-biphenyl boronic acid (9.90 mg, 0.050 mmol, 1.00 equiv.), brettphos (1.61 mg, 0.003 mmol, 0.060 equiv.) and potassium fluoride (5.81 mg, 0.100 mmol, 2.00 equiv.) at 23 °C. In another 4 mL borosilicate vial (vial 2), propargyl thianthrenium hexafluorophosphate (50.0 mg, 0.125 mmol, 2.50 equiv.) was added at 23 °C. Both vials were sealed with Teflon-lined screw caps and then they were transferred to a N_2 filled glovebox. $Pd(P^tBu_3)_2$ (0.800 mg, 0.015 mmol, 0.030 equiv.) was added to vial 1 followed by the addition of dry toluene (1.0 mL, c = 0.050 M). The resulting reaction mixture was stirred (500 rpm) at 23 °C for a minute. Then, propargyl thianthrenium hexafluorophosphate salt from vial 2 was added at once to vial 1 and it was sealed using a Teflon-lined screw cap. The sealed vial 1 was transferred out of the glovebox, placed on a heating block, and stirred (500 rpm) at 30 °C for 7 hours. Then, the solvent was concentrated under reduced pressure and diluted with 0.7 mL $CDCl_3$, followed by the addition of mesitylene as an internal standard (7.0 μL, 0.05 mmol, 1.0 equiv.). The resulting mixture was

passed through celite and submitted for ^{1}H NMR measurement to check the NMR yield. From the ^{1}H NMR measurement it was confirmed that product **12** is formed with 23% yield.

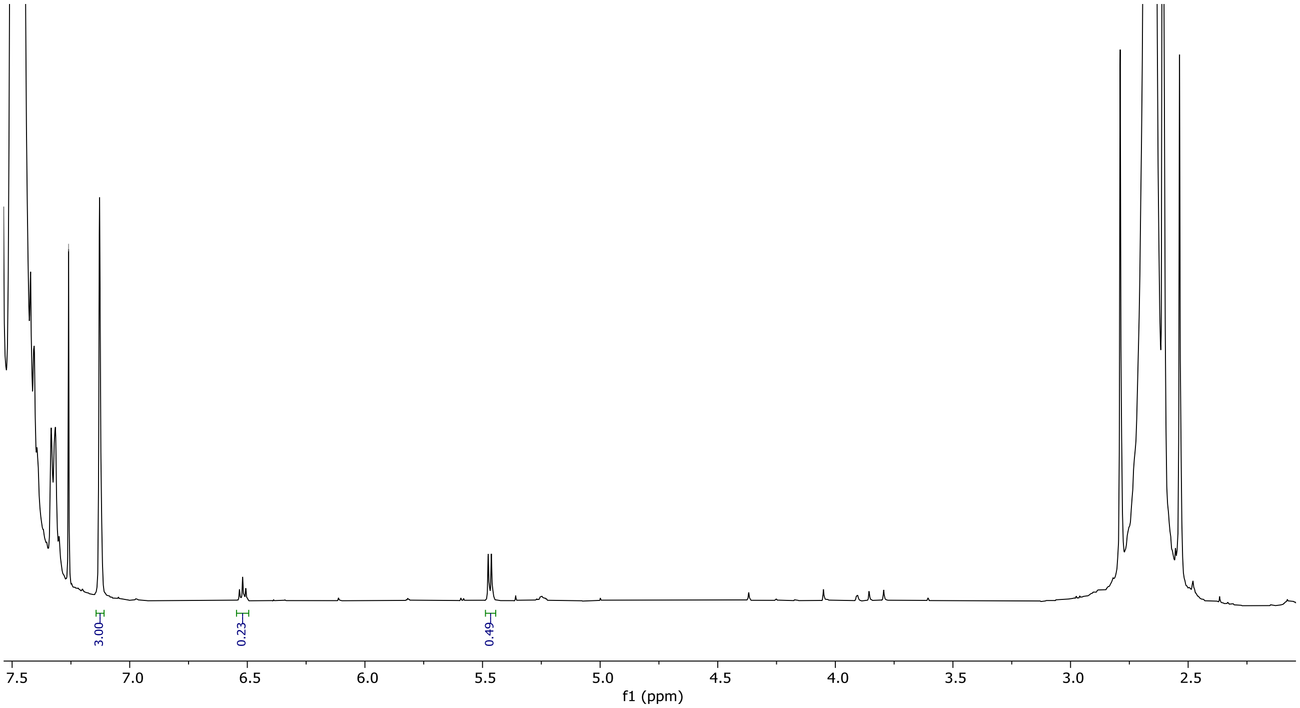

Figure S15. ^{1}H NMR spectra of the crude sample taken after the reaction of 4-biphenyl boronic acid with propargyl thianthrenium hexafluorophosphate, $CDCl_3$, 500 MHz, 23 ºC using mesitylene as an internal standard.

From the above results, it was interpreted that the propargyl thianthrenium salts reacted in a similar way as that of its allenyl isomers **1-BF$_4$** and **1**, although producing lower yields, as determined by the ^{1}H NMR.

X-RAY CRYSTALLOGRAPHIC ANALYSIS

Allenyl thianthrenium hexafluorophosphate (ATT-PF_6, 1) (CCDC 2336789)

Experimental

ATT (**1**) was crystallized from dichloromethane/pentane. The atoms are depicted with 50% probability ellipsoids. The crystallographic data are summarized in the following table.

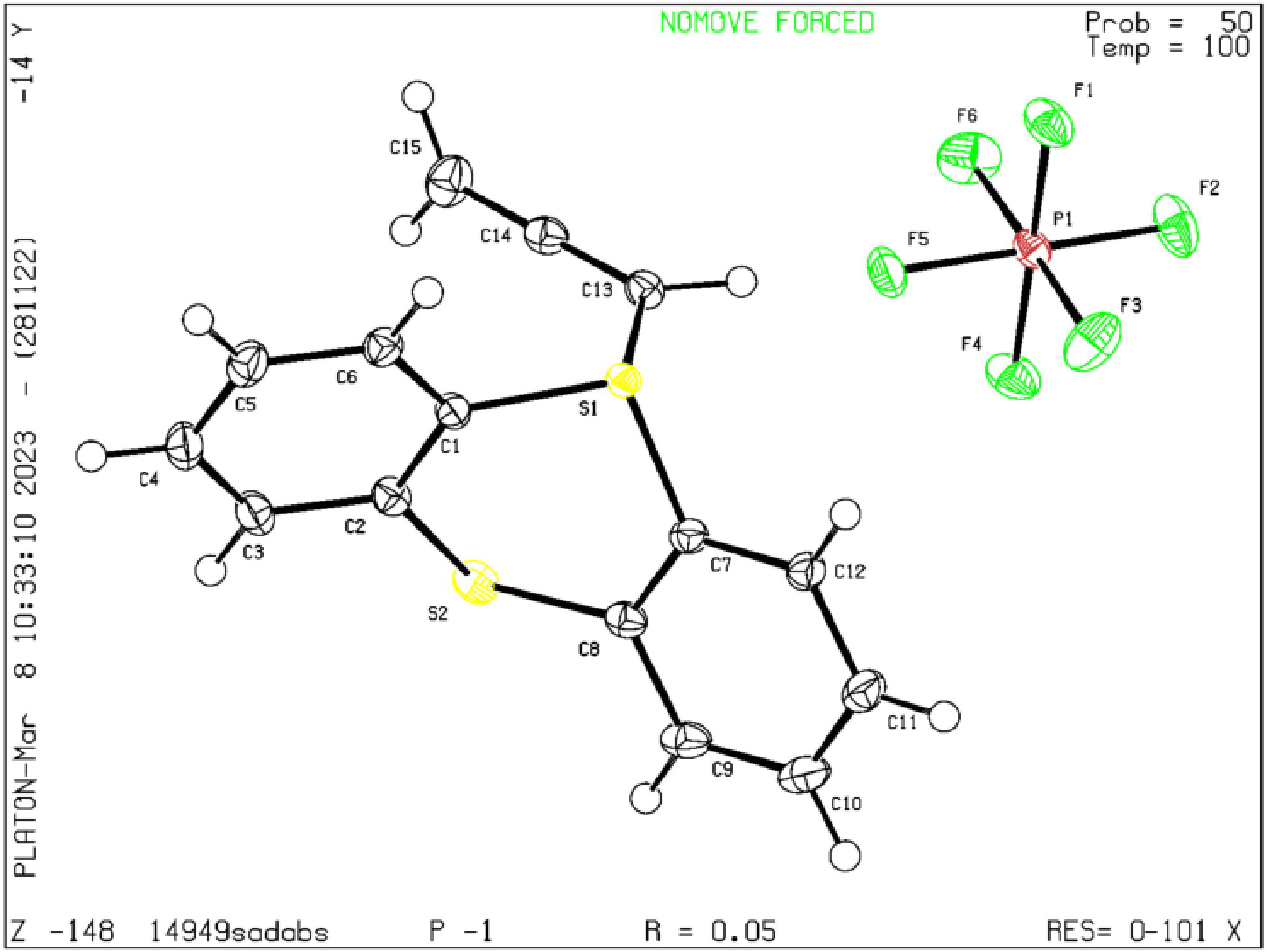

Figure S16: X-ray structure of ATT-PF_6 (**1**)

Table 1. Crystal data and structure refinement.

Identification code	14949
Empirical formula	$C_{15}H_{11}F_6PS_2$
Color	colourless
Formula weight	400.351 g · mol^{-1}
Temperature	100(2) K
Wavelength	0.71073 Å

Crystal system	TRICLINIC	
Space group	**P$\bar{1}$, (no. 2)**	
Unit cell dimensions	a = 9.2395(10) Å	α= 75.056(4)°.
	b = 9.6103(10) Å	β= 80.237(4)°.
	c = 9.7623(10) Å	γ = 73.293(4)°.
Volume	797.89(15) $Å^3$	
Z	2	
Density (calculated)	1.666 Mg · m^{-3}	
Absorption coefficient	0.492 mm^{-1}	
F(000)	404.991 e	
Crystal size	0.448 x 0.160 x 0.071 mm^3	
θ range for data collection	2.17 to 33.21°.	
Index ranges	-14 ≤ h ≤ 14, -14≤ k ≤ 14, -15 ≤ l ≤ 15	
Reflections collected	107890	
Independent reflections	6089 [R_{int} = 0.0534]	
Reflections with I>2σ(I)	5734	
Completeness to θ = 25.2417°	99.59 %	
Absorption correction	Semi-empirical from equivalents	
Max. and min. transmission	1.00 and 0.91	
Refinement method	Full-matrix least-squares on F^2	
Data / restraints / parameters	6089 / 0 / 229	
Goodness-of-fit on F^2	1.0114	
Final R indices [I>2σ(I)]	R_1 = 0.0462	wR^2 = 0.1357
R indices (all data)	R_1 = 0.0479	wR^2 = 0.1366
Largest diff. peak and hole	0.8 and -0.5 e · $Å^{-3}$	

Table 2. Bond lengths [Å] and angles [°].

S(1)-C(1)	1.760(2)	S(1)-C(7)	1.763(2)
S(1)-C(13)	1.795(2)	S(2)-C(2)	1.761(2)
S(2)-C(8)	1.759(2)	C(1)-C(2)	1.396(3)
C(1)-C(6)	1.395(3)	C(2)-C(3)	1.394(3)
C(3)-C(4)	1.393(4)	C(4)-C(5)	1.389(4)
C(5)-C(6)	1.388(3)	C(7)-C(8)	1.397(3)
C(7)-C(12)	1.393(3)	C(8)-C(9)	1.396(3)

C(9)-C(10)	1.387(4)	C(10)-C(11)	1.390(4)
C(11)-C(12)	1.389(3)	C(13)-C(14)	1.303(3)
C(14)-C(15)	1.298(3)	P(1)-F(1)	1.5955(16)
P(1)-F(2)	1.5923(16)	P(1)-F(3)	1.6049(17)
P(1)-F(4)	1.6055(16)	P(1)-F(5)	1.6124(16)
P(1)-F(6)	1.5998(17)		

C(7)-S(1)-C(1)	102.80(10)	C(13)-S(1)-C(1)	104.07(10)
C(13)-S(1)-C(7)	102.77(10)	C(8)-S(2)-C(2)	101.96(10)
C(2)-C(1)-S(1)	121.71(16)	C(6)-C(1)-S(1)	116.35(16)
C(6)-C(1)-C(2)	121.9(2)	C(1)-C(2)-S(2)	123.42(17)
C(3)-C(2)-S(2)	118.38(17)	C(3)-C(2)-C(1)	118.2(2)
C(4)-C(3)-C(2)	120.1(2)	C(5)-C(4)-C(3)	121.0(2)
C(6)-C(5)-C(4)	119.7(2)	C(5)-C(6)-C(1)	119.0(2)
C(8)-C(7)-S(1)	121.19(16)	C(12)-C(7)-S(1)	116.61(16)
C(12)-C(7)-C(8)	122.2(2)	C(7)-C(8)-S(2)	123.83(17)
C(9)-C(8)-S(2)	118.04(18)	C(9)-C(8)-C(7)	118.1(2)
C(10)-C(9)-C(8)	120.2(2)	C(11)-C(10)-C(9)	120.8(2)
C(12)-C(11)-C(10)	120.2(2)	C(11)-C(12)-C(7)	118.5(2)
C(14)-C(13)-S(1)	122.12(17)	C(15)-C(14)-C(13)	178.9(2)
F(2)-P(1)-F(1)	90.44(9)	F(3)-P(1)-F(1)	90.29(10)
F(3)-P(1)-F(2)	90.31(10)	F(4)-P(1)-F(1)	179.08(9)
F(4)-P(1)-F(2)	90.46(9)	F(4)-P(1)-F(3)	89.48(10)
F(5)-P(1)-F(1)	89.43(8)	F(5)-P(1)-F(2)	179.85(9)
F(5)-P(1)-F(3)	89.61(9)	F(5)-P(1)-F(4)	89.67(9)
F(6)-P(1)-F(1)	90.36(10)	F(6)-P(1)-F(2)	90.71(10)
F(6)-P(1)-F(3)	178.78(11)	F(6)-P(1)-F(4)	89.84(10)
F(6)-P(1)-F(5)	89.38(10)		

Propargyl thianthrenium triflate (PTT-OTf, 1a) (CCDC 2336790)

Experimental

PTT (**1a**) was crystallized from dichloromethane/diethyl ether. The atoms are depicted with 50% probability ellipsoids. The crystallographic data are summarized in the following table.

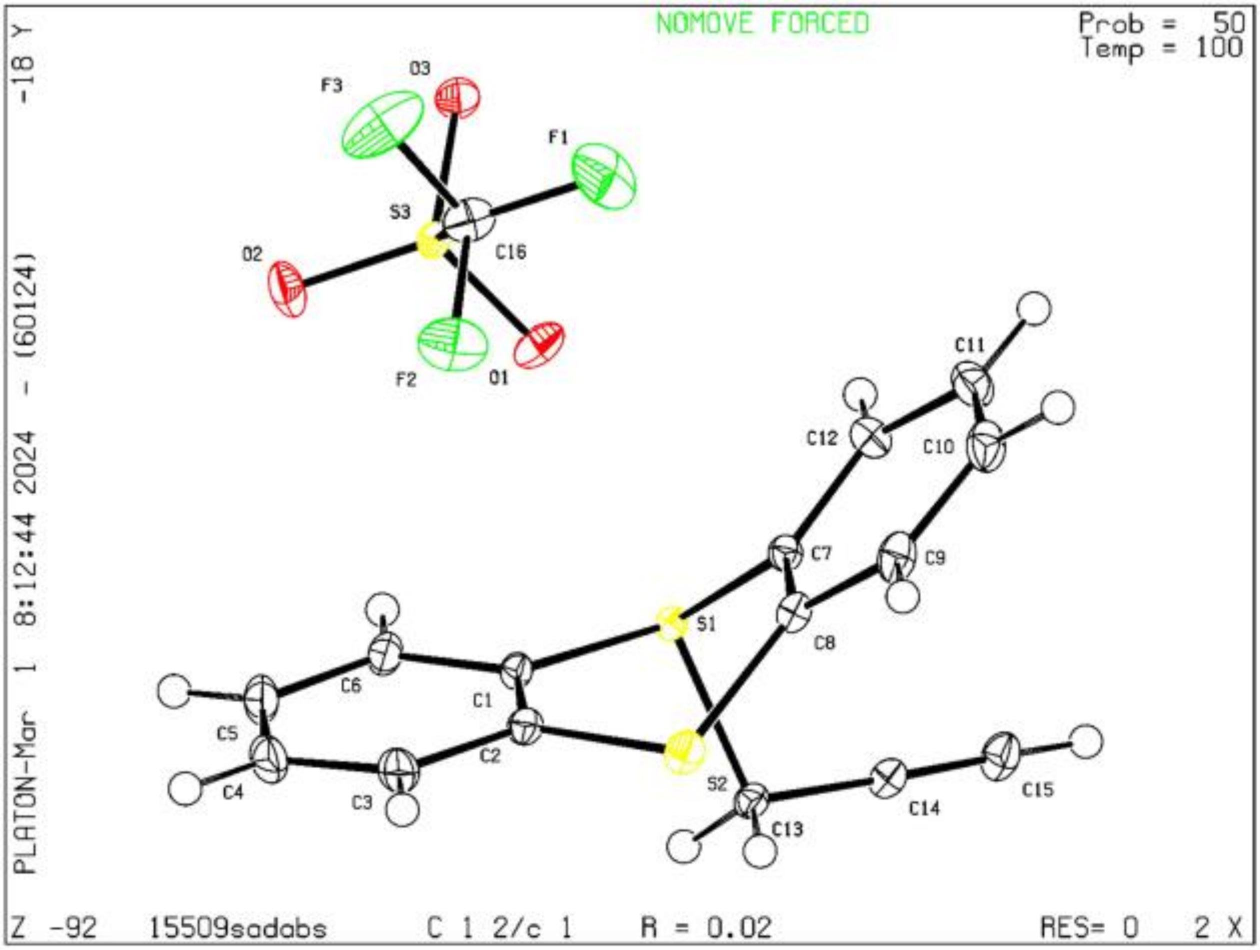

Figure S17: X-ray structure of PTT-OTf (**1a**)

Table 1. Crystal data and structure refinement.

Identification code	15509
Empirical formula	$C_{16} H_{11} F_3 O_3 S_3$
Color	colourless
Formula weight	404.43 g·mol^{-1}
Temperature	100(2) K
Wavelength	0.71073 Å

Crystal system	MONOCLINIC	
Space group	**C2/c, (no. 15)**	
Unit cell dimensions	a = 16.6423(12) Å	α= 90°.
	b = 9.1069(6) Å	β= 103.584(2)°.
	c = 23.0774(16) Å	γ = 90°.
Volume	3399.8(4) $Å^3$	
Z	8	
Density (calculated)	1.580 Mg · m^{-3}	
Absorption coefficient	0.480 mm^{-1}	
F(000)	1648 e	
Crystal size	0.38 x 0.187 x 0.16 mm^3	
θ range for data collection	1.816 to 34.888°.	
Index ranges	-26 ≤ h ≤ 26, -14≤ k ≤ 14, -37 ≤ l ≤ 37	
Reflections collected	65652	
Independent reflections	7396 [R_{int} = 0.0189]	
Reflections with I>2σ(I)	6975	
Completeness to θ = 25.242°	100.0 %	
Absorption correction	Gaussian	
Max. and min. transmission	0.94 and 0.85	
Refinement method	Full-matrix least-squares on F^2	
Data / restraints / parameters	7396 / 0 / 270	
Goodness-of-fit on F^2	1.060	
Final R indices [I>2σ(I)]	R_1 = 0.0248	wR^2 = 0.0704
R indices (all data)	R_1 = 0.0266	wR^2 = 0.0717
Largest diff. peak and hole	0.6 and -0.5 e · $Å^{-3}$	

Table 2. Bond lengths [Å] and angles [°].

S(1)-C(1)	1.7642(7)	S(1)-C(7)	1.7598(7)
S(1)-C(13)	1.8410(7)	S(2)-C(2)	1.7525(8)
S(2)-C(8)	1.7513(8)	C(1)-C(2)	1.3971(10)
C(1)-C(6)	1.3951(10)	C(2)-C(3)	1.3995(11)
C(3)-H(3)	0.976(15)	C(3)-C(4)	1.3837(13)
C(4)-H(4)	0.929(15)	C(4)-C(5)	1.3944(14)
C(5)-H(5)	0.914(15)	C(5)-C(6)	1.3872(11)
C(6)-H(6)	0.957(14)	C(7)-C(8)	1.3961(10)
C(7)-C(12)	1.3942(10)	C(8)-C(9)	1.3974(11)
C(9)-H(9)	0.957(15)	C(9)-C(10)	1.3877(12)
C(10)-H(10)	0.973(15)	C(10)-C(11)	1.3931(13)
C(11)-H(11)	0.952(15)	C(11)-C(12)	1.3883(11)
C(12)-H(12)	0.986(14)	C(13)-H(13A)	0.973(14)
C(13)-H(13B)	0.943(13)	C(13)-C(14)	1.4511(10)
C(14)-C(15)	1.1980(11)	C(15)-H(15)	0.899(15)
S(3)-O(1)	1.4427(6)	S(3)-O(2)	1.4392(6)
S(3)-O(3)	1.4434(6)	S(3)-C(16)	1.8286(8)
F(1)-C(16)	1.3360(11)	F(2)-C(16)	1.3353(10)
F(3)-C(16)	1.3256(10)		

C(1)-S(1)-C(13)	101.44(3)	C(7)-S(1)-C(1)	103.03(3)
C(7)-S(1)-C(13)	102.23(3)	C(8)-S(2)-C(2)	103.10(3)
C(2)-C(1)-S(1)	121.44(5)	C(6)-C(1)-S(1)	116.85(6)
C(6)-C(1)-C(2)	121.70(7)	C(1)-C(2)-S(2)	124.89(5)
C(1)-C(2)-C(3)	118.39(7)	C(3)-C(2)-S(2)	116.67(6)
C(2)-C(3)-H(3)	119.7(9)	C(4)-C(3)-C(2)	120.17(8)
C(4)-C(3)-H(3)	120.1(9)	C(3)-C(4)-H(4)	119.5(10)
C(3)-C(4)-C(5)	120.73(8)	C(5)-C(4)-H(4)	119.7(10)
C(4)-C(5)-H(5)	119.2(10)	C(6)-C(5)-C(4)	120.05(8)
C(6)-C(5)-H(5)	120.7(10)	C(1)-C(6)-H(6)	119.6(9)
C(5)-C(6)-C(1)	118.88(8)	C(5)-C(6)-H(6)	121.5(9)
C(8)-C(7)-S(1)	122.36(5)	C(12)-C(7)-S(1)	115.91(5)
C(12)-C(7)-C(8)	121.68(7)	C(7)-C(8)-S(2)	124.00(5)
C(7)-C(8)-C(9)	118.23(7)	C(9)-C(8)-S(2)	117.77(6)
C(8)-C(9)-H(9)	118.8(9)	C(10)-C(9)-C(8)	120.20(7)

C(10)-C(9)-H(9)	121.0(9)	C(9)-C(10)-H(10)	120.9(9)
C(9)-C(10)-C(11)	121.01(7)	C(11)-C(10)-H(10)	118.1(9)
C(10)-C(11)-H(11)	121.2(9)	C(12)-C(11)-C(10)	119.43(8)
C(12)-C(11)-H(11)	119.3(9)	C(7)-C(12)-H(12)	120.6(8)
C(11)-C(12)-C(7)	119.34(7)	C(11)-C(12)-H(12)	120.0(8)
S(1)-C(13)-H(13A)	108.3(8)	S(1)-C(13)-H(13B)	103.7(8)
H(13A)-C(13)-H(13B)	110.2(12)	C(14)-C(13)-S(1)	107.85(5)
C(14)-C(13)-H(13A)	113.6(8)	C(14)-C(13)-H(13B)	112.6(8)
C(15)-C(14)-C(13)	178.38(8)	C(14)-C(15)-H(15)	178.9(9)
O(1)-S(3)-O(3)	114.48(4)	O(1)-S(3)-C(16)	102.32(4)
O(2)-S(3)-O(1)	115.12(4)	O(2)-S(3)-O(3)	115.25(4)
O(2)-S(3)-C(16)	103.47(4)	O(3)-S(3)-C(16)	103.79(4)
F(1)-C(16)-S(3)	110.48(6)	F(2)-C(16)-S(3)	111.38(6)
F(2)-C(16)-F(1)	107.38(7)	F(3)-C(16)-S(3)	111.87(6)
F(3)-C(16)-F(1)	107.54(8)	F(3)-C(16)-F(2)	107.98(8)

SPECTROSCOPIC DATA

^{1}H NMR of allenyl thianthrenium hexafluorophosphate (1)

CD_3CN, 23 ºC

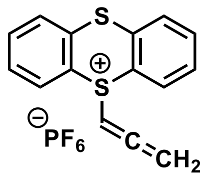

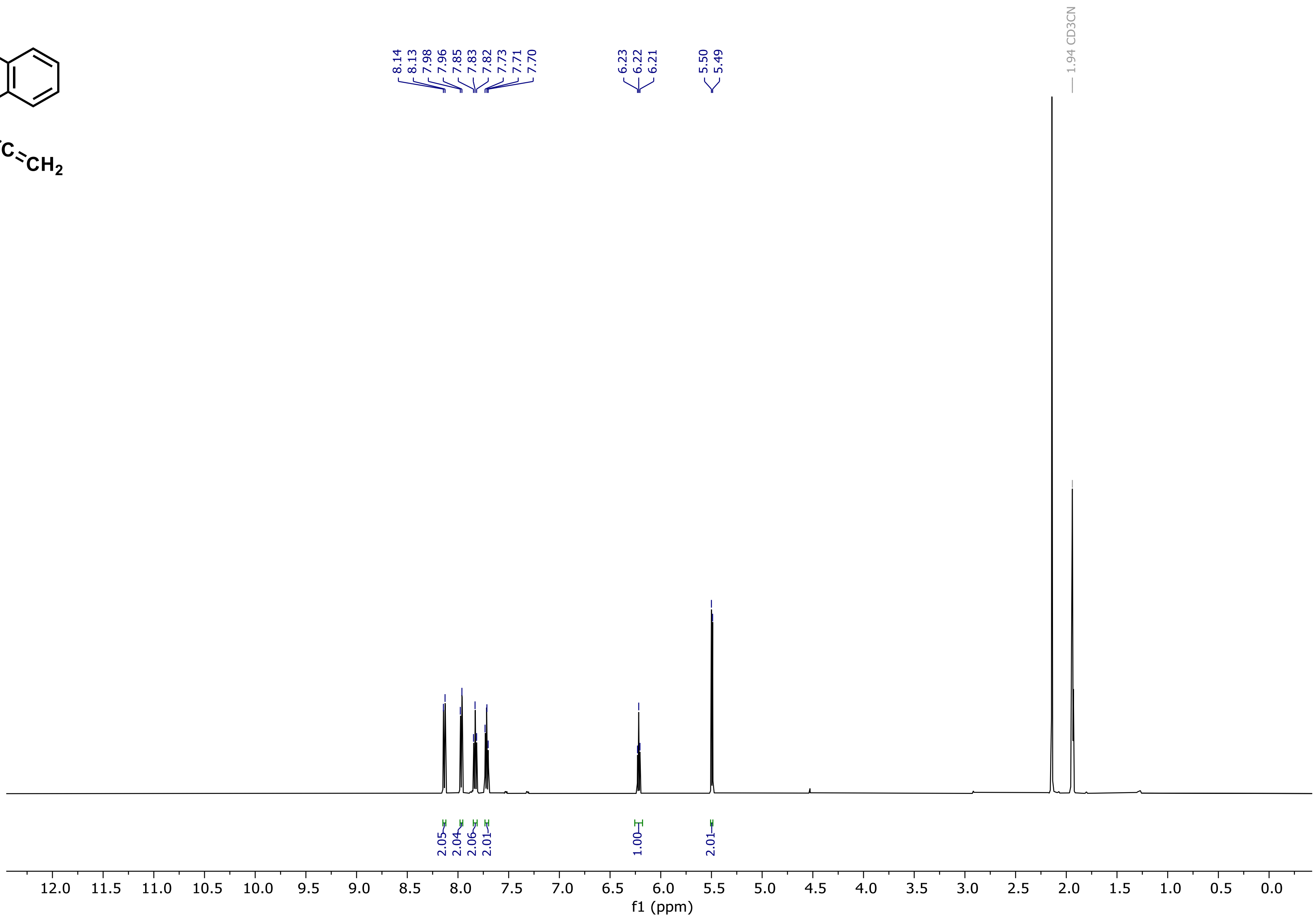

^{13}C NMR of allenyl thianthrenium hexafluorophosphate (1)

CD_3CN, 23 ºC

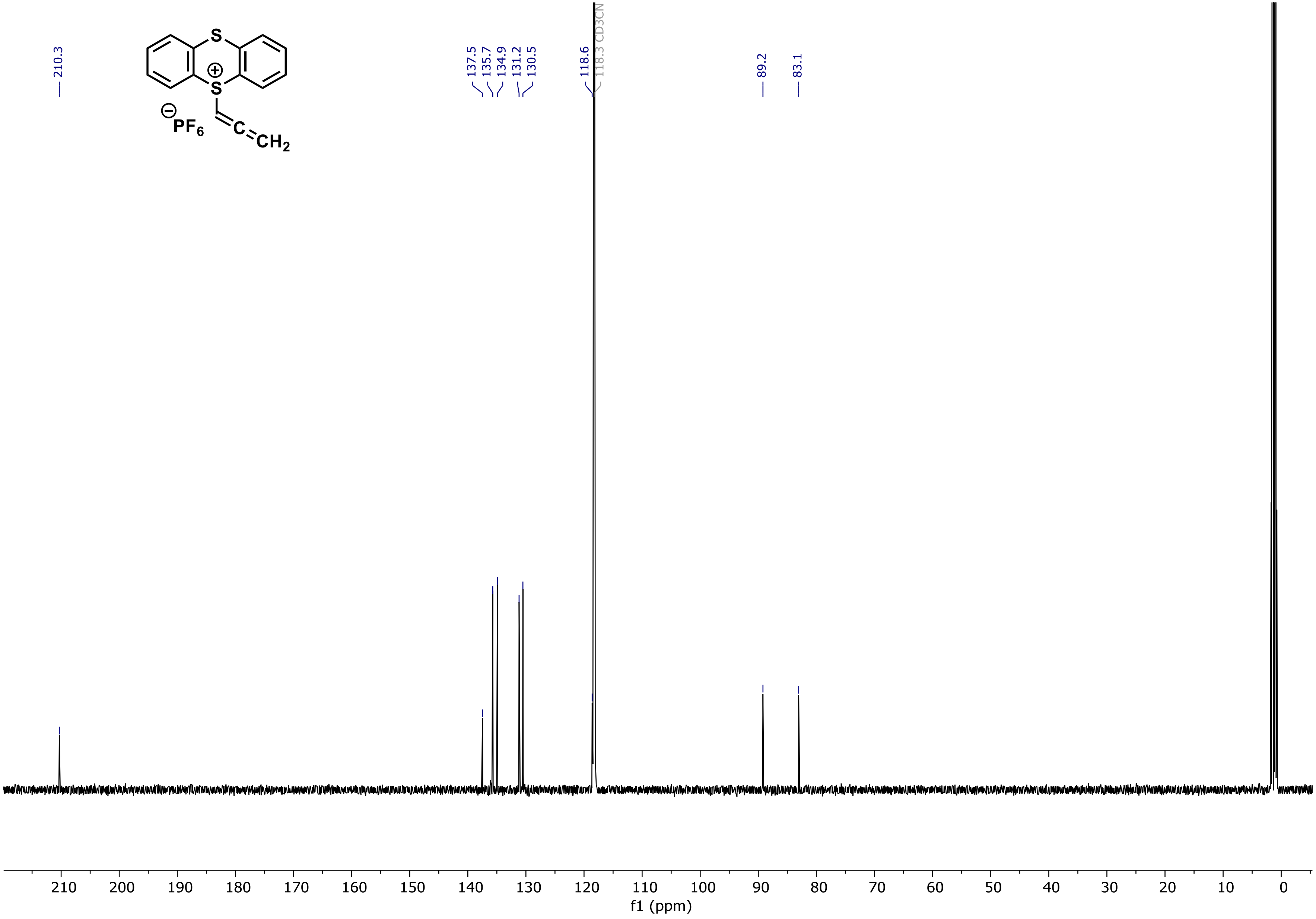

^{19}F NMR of allenyl thianthrenium hexafluorophosphate (1)

CD_3CN, 23 ºC

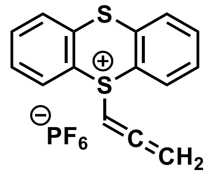

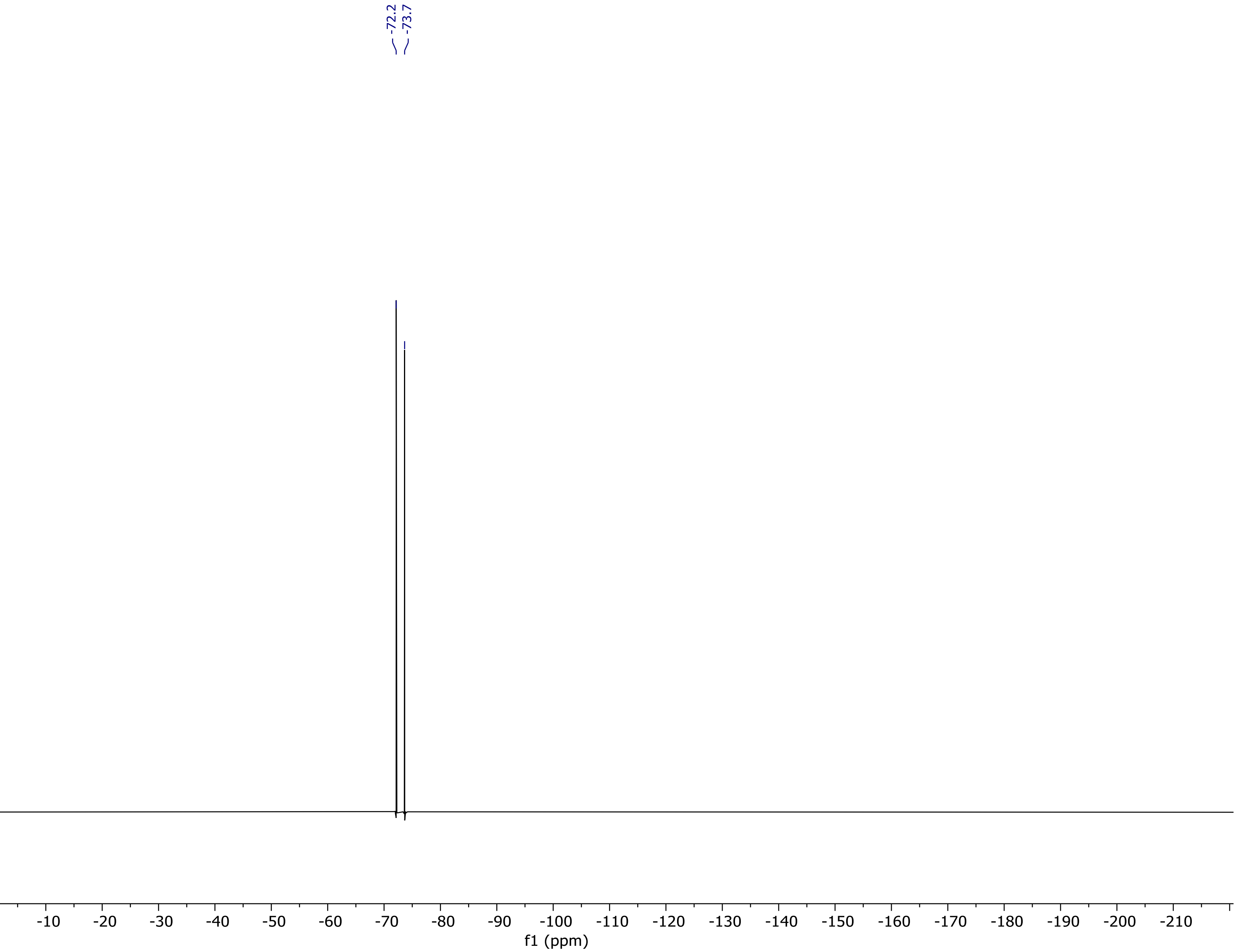

^{1}H NMR of allenyl thianthrenium tetrafluoroborate (1-BF_4)

CD_3CN, 23 ºC

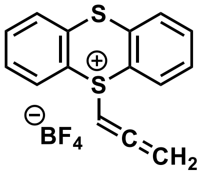

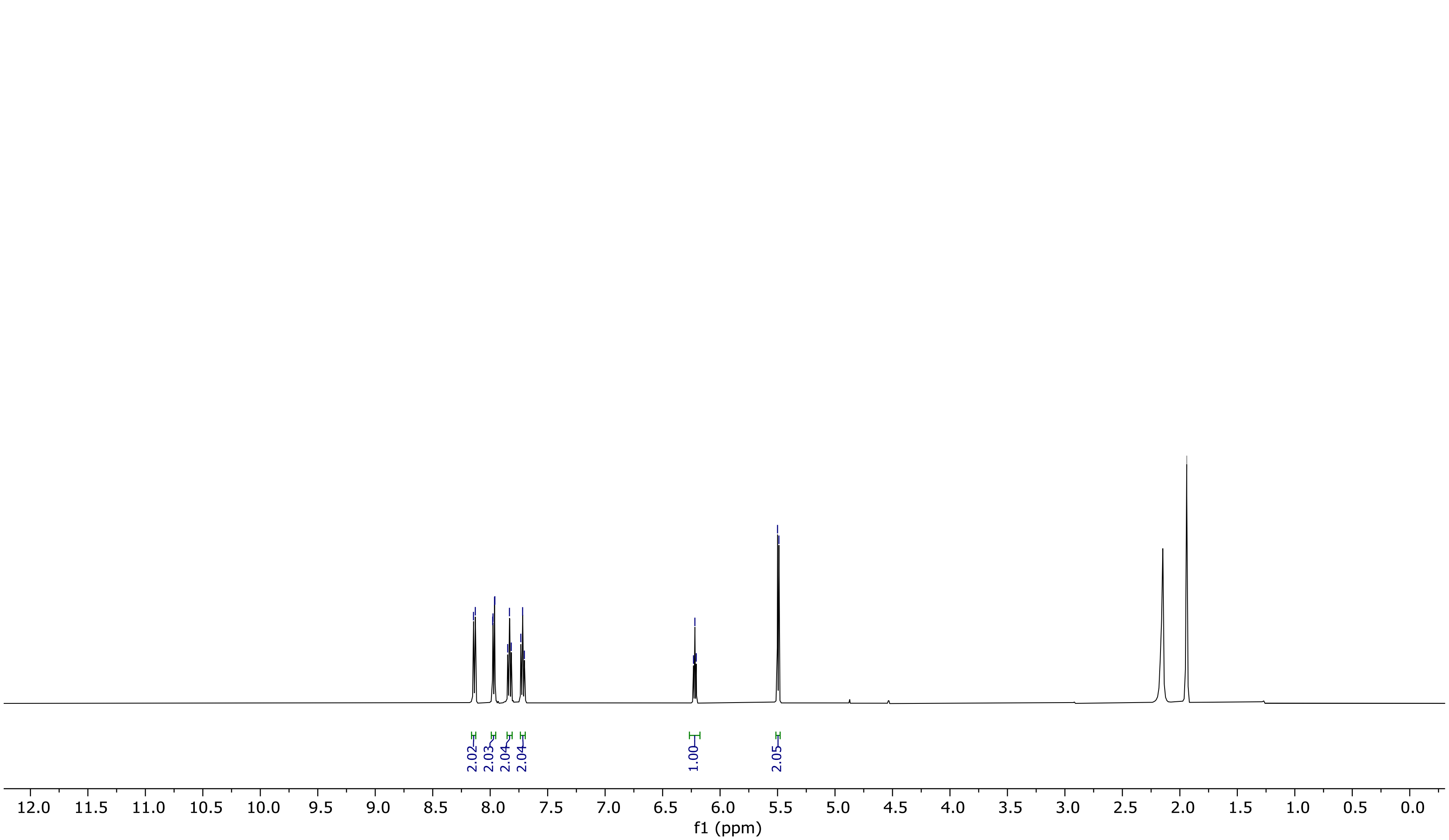

^{13}C NMR of allenyl thianthrenium tetrafluoroborate (1-BF_4)

CD_3CN, 23 ºC

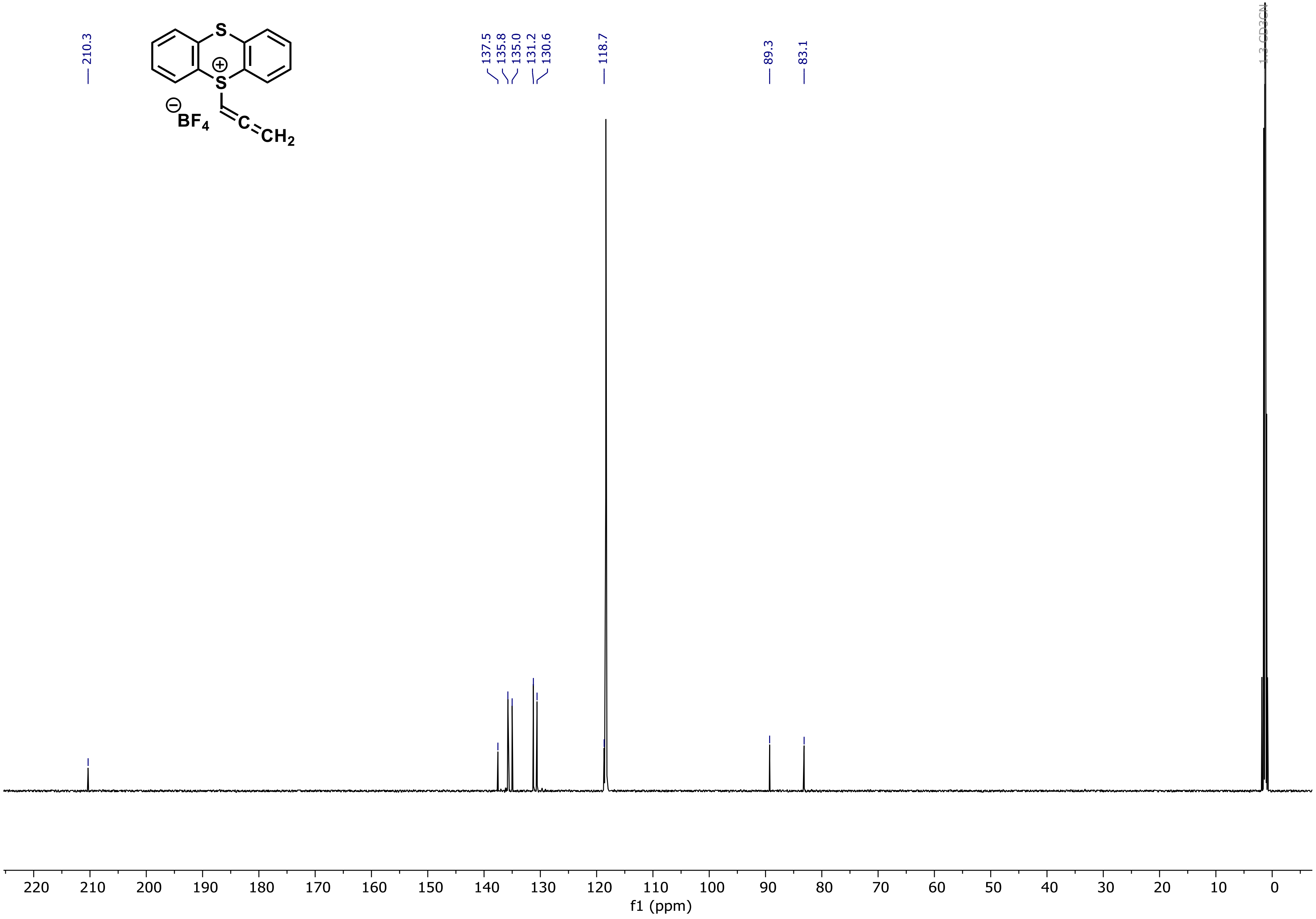

^{19}F NMR of allenyl thianthrenium tetrafluoroborate (1-BF_4)

CD_3CN, 23 ºC

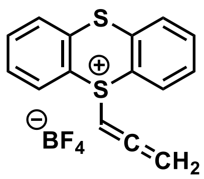

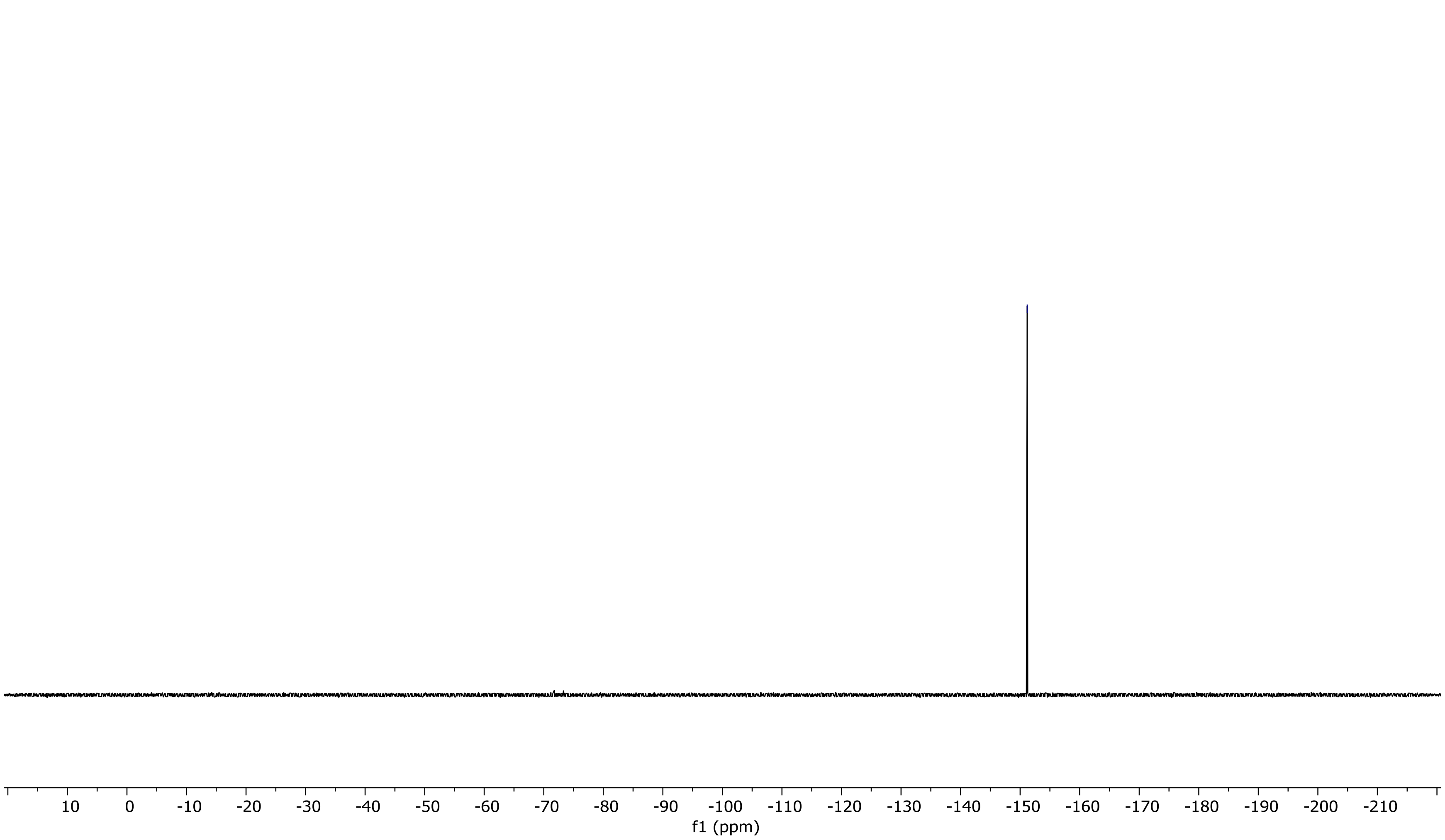

^{1}H NMR of propargyl thianthrenium triflate (1a)

CD_3CN, 23 ºC

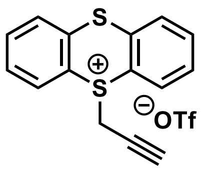

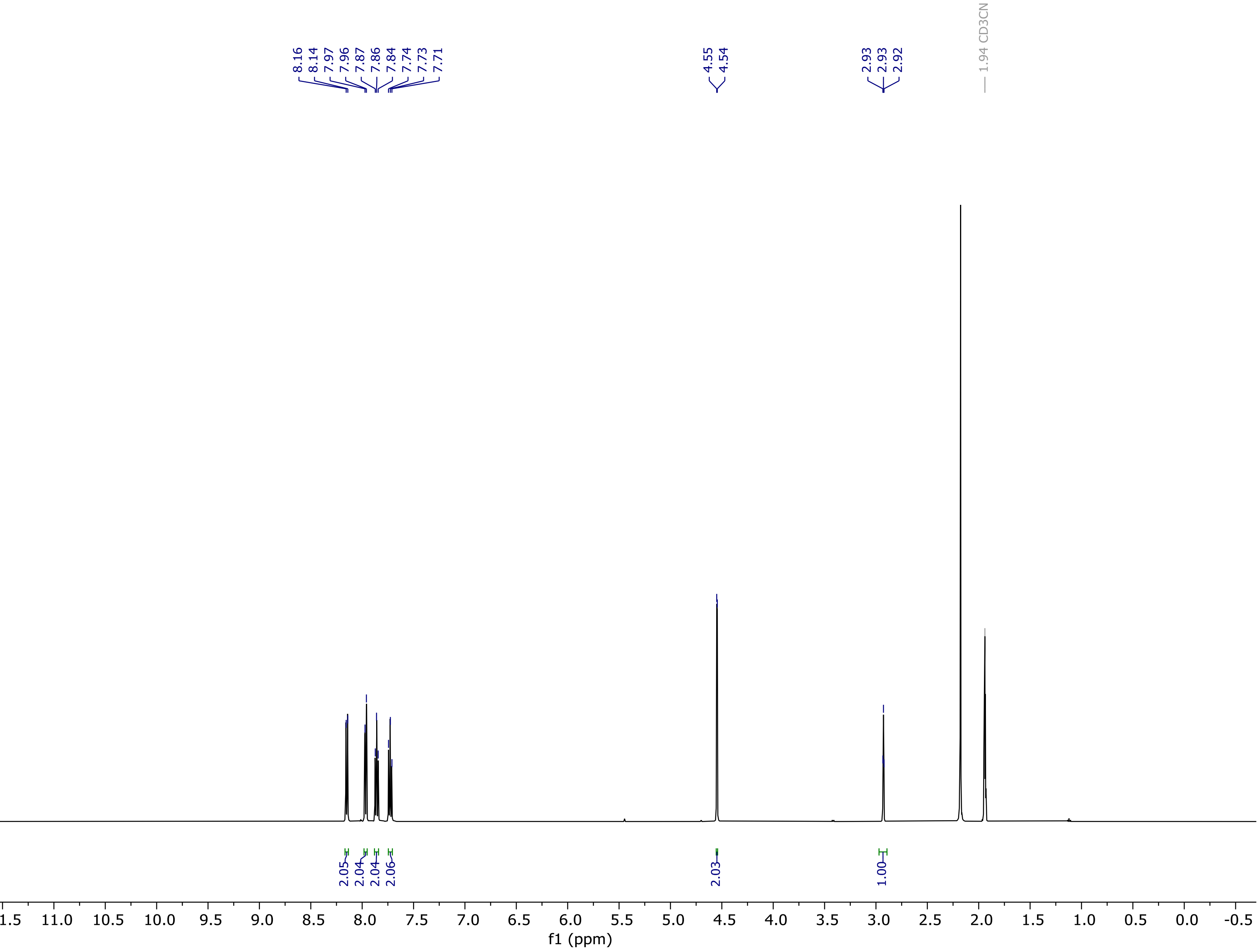

^{13}C NMR of propargyl thianthrenium triflate (1a)

CD_3CN, 23 ºC

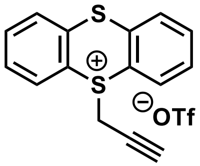

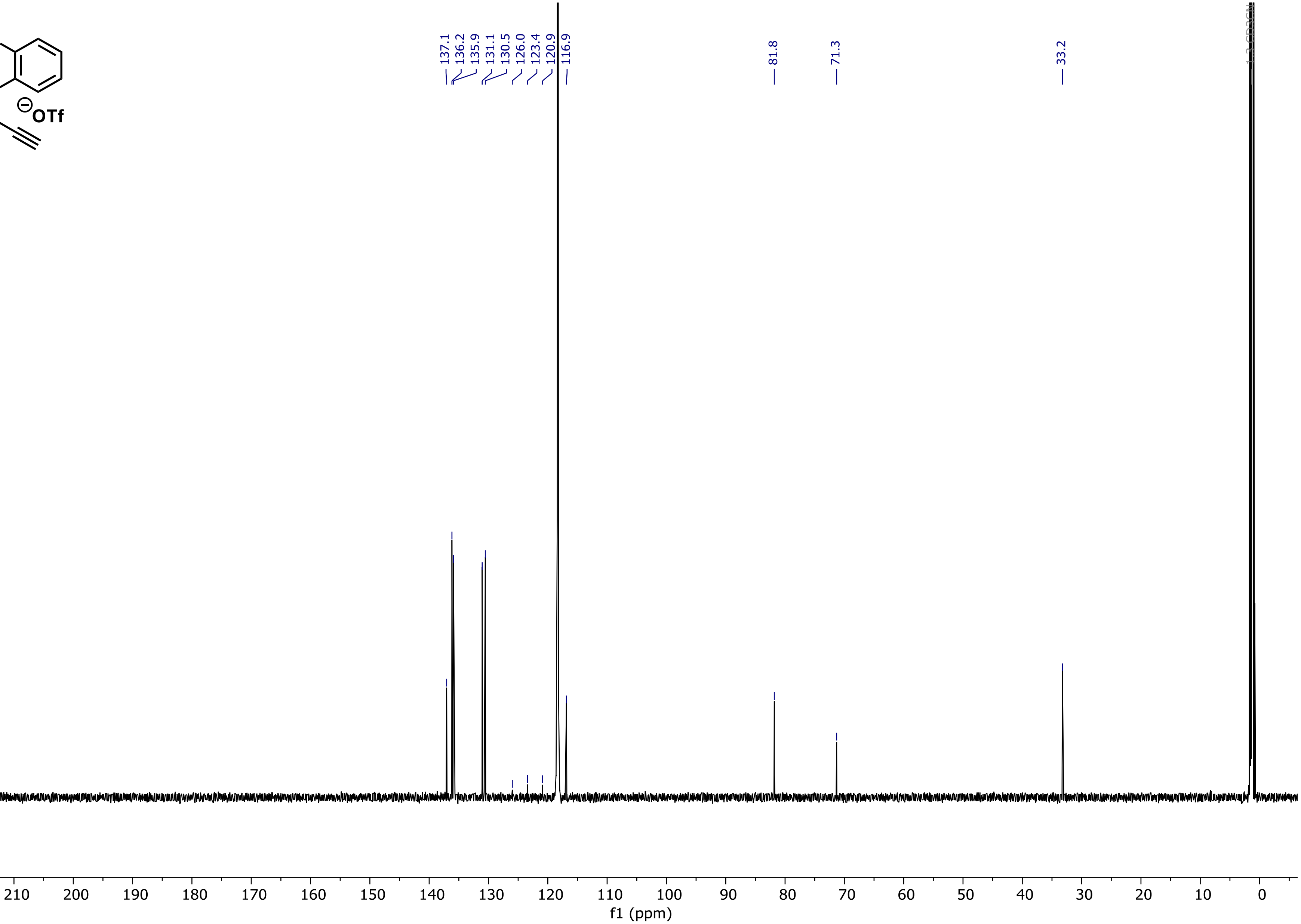

^{19}F NMR of propargyl thianthrenium triflate (1a)

CD_3CN, 23 ºC

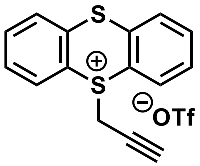

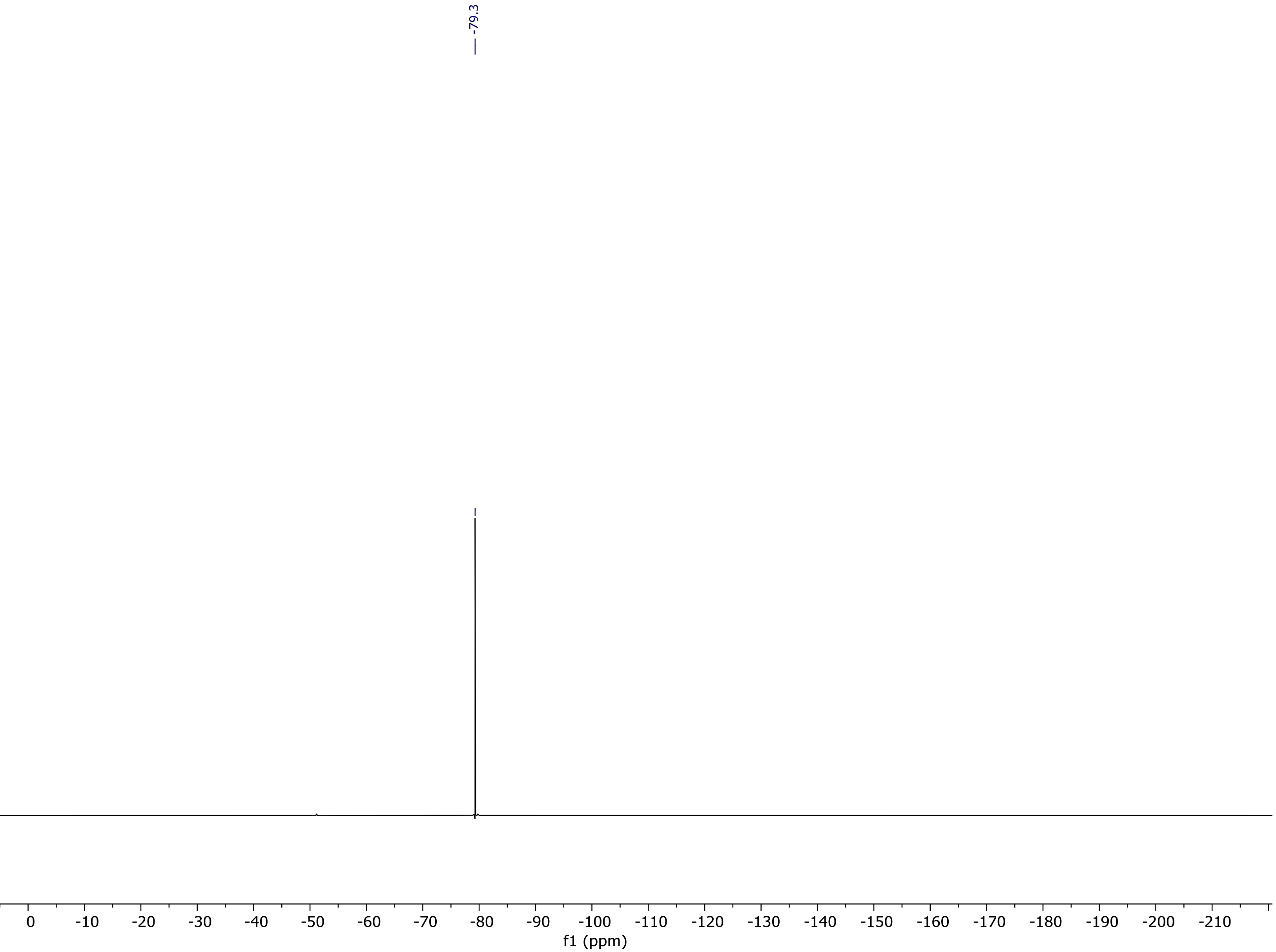

^{1}H NMR of 2,6,7-trimethylquinoxaline (2)

$CDCl_3$, 23 ºC

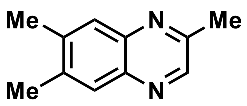

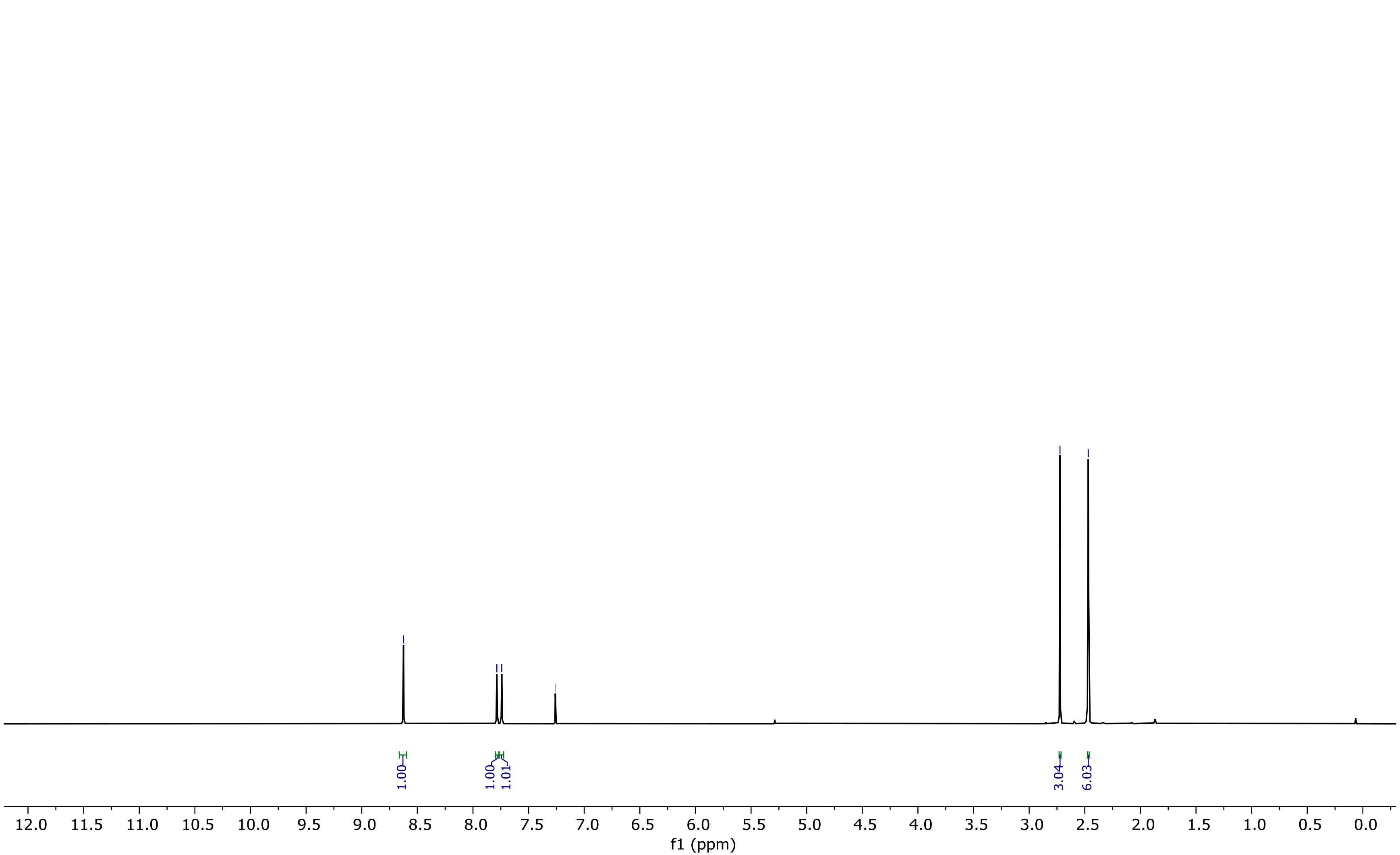

^{13}C NMR of 2,6,7-trimethylquinoxaline (2)

$CDCl_3$, 23 ºC

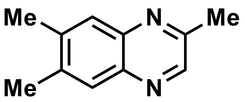

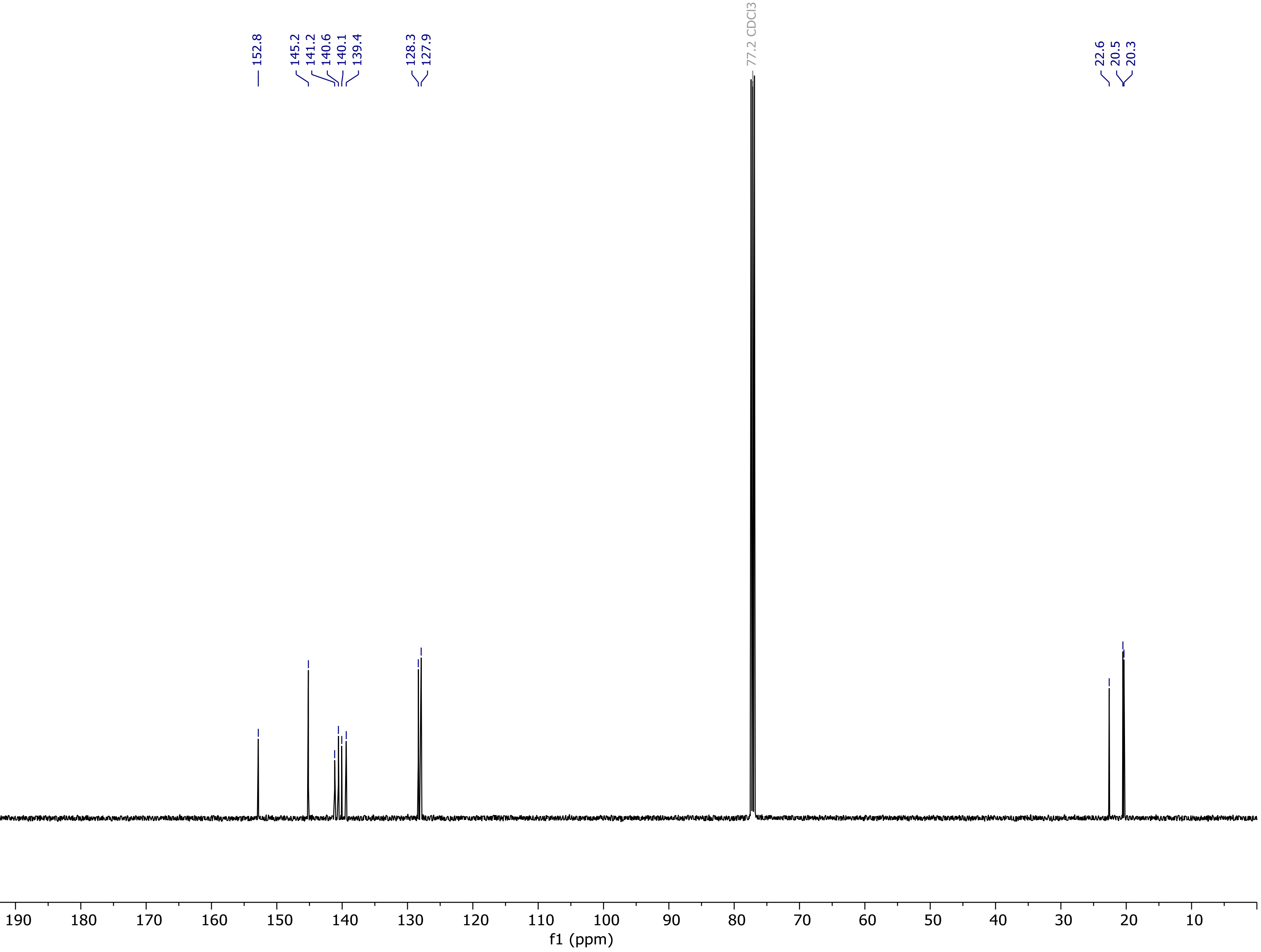

^{1}H NMR of 6,7-dichloro-(2-methyl)quinoxaline (3)

$CDCl_3$, 23 ºC

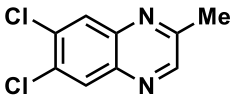

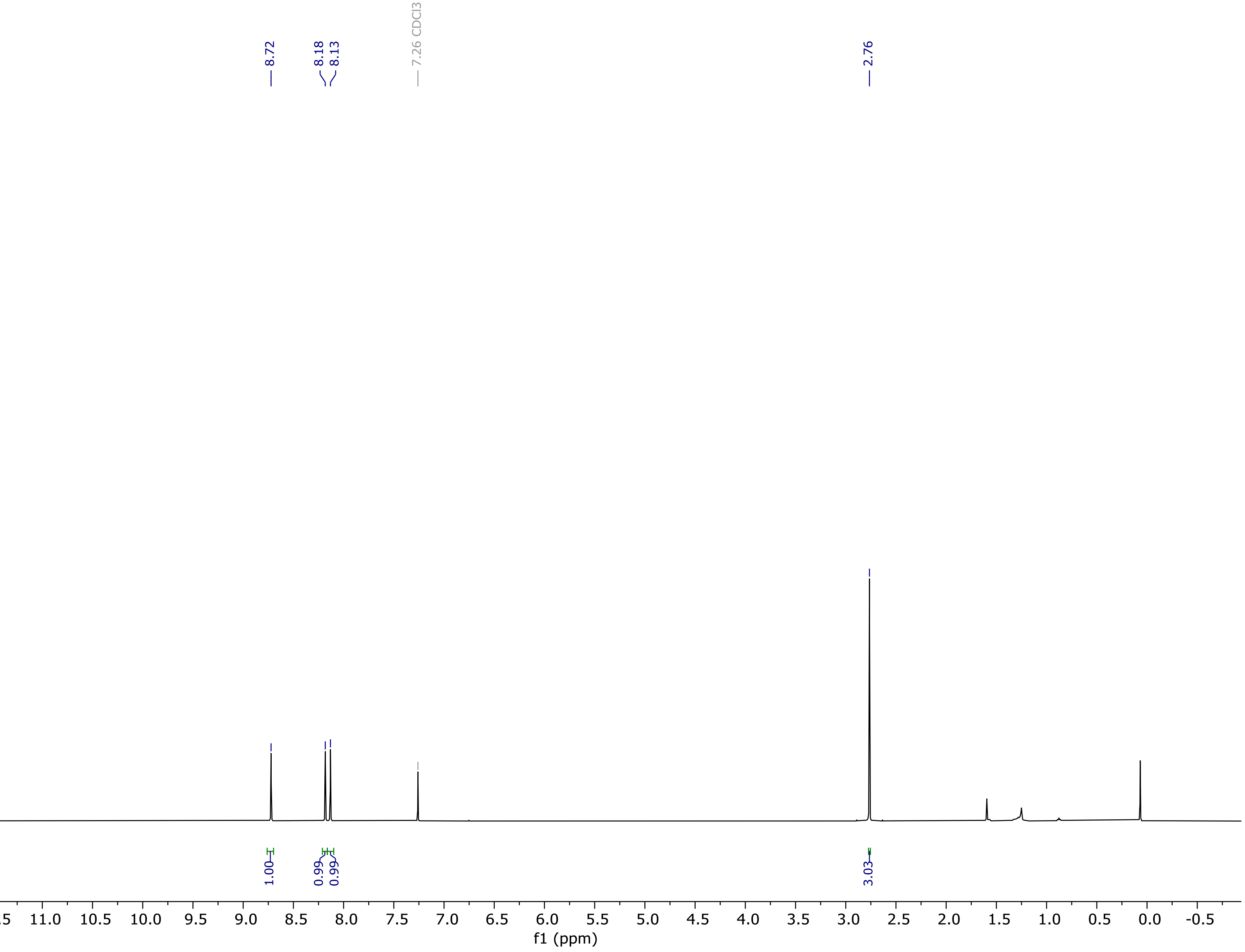

^{13}C NMR of 6,7-dichloro-(2-methyl)quinoxaline (3)

$CDCl_3$, 23 ºC

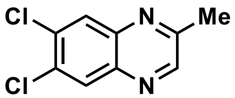

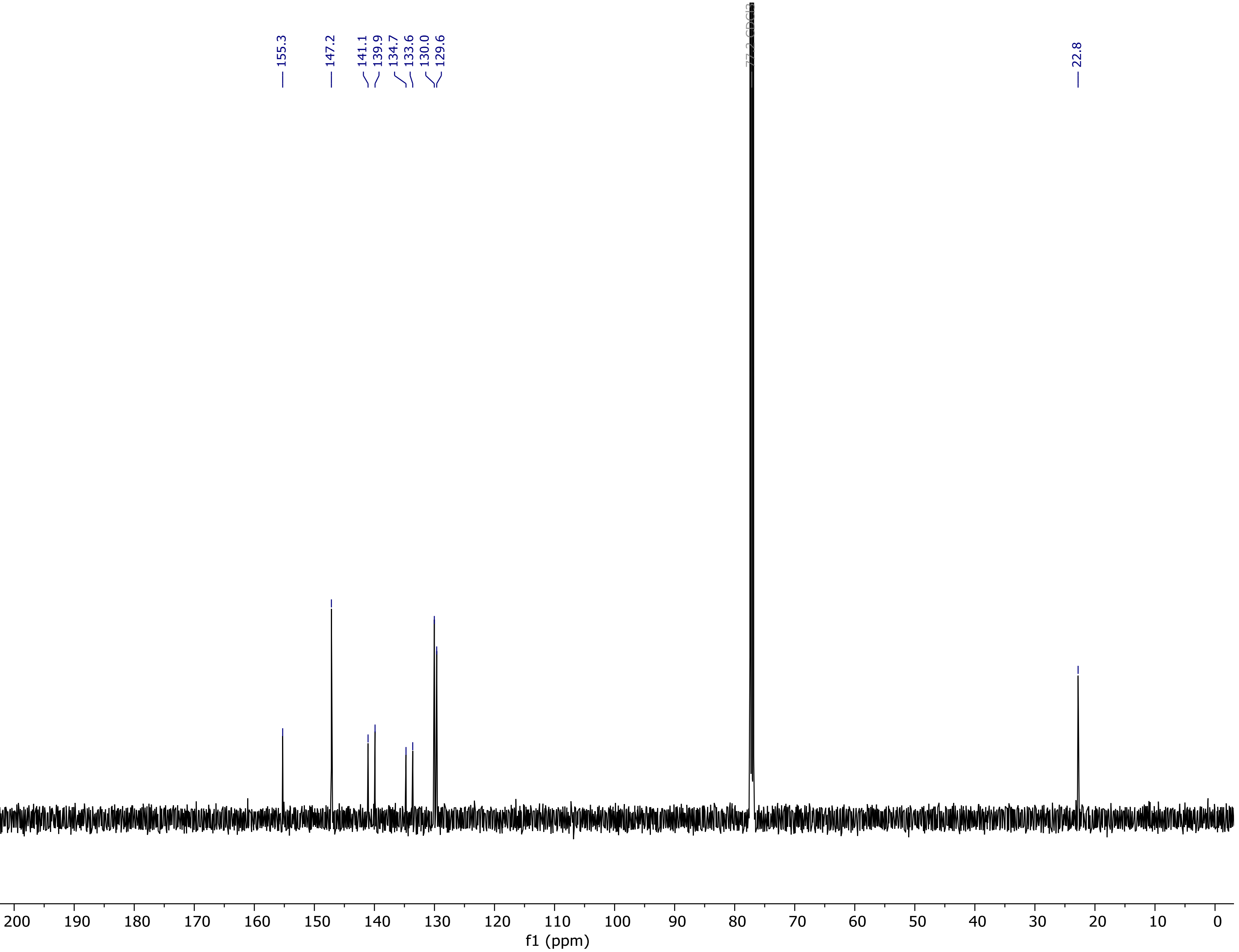

^{1}H NMR of 2-methylquinoxaline (4)

$CDCl_3$, 23 ºC

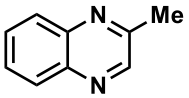

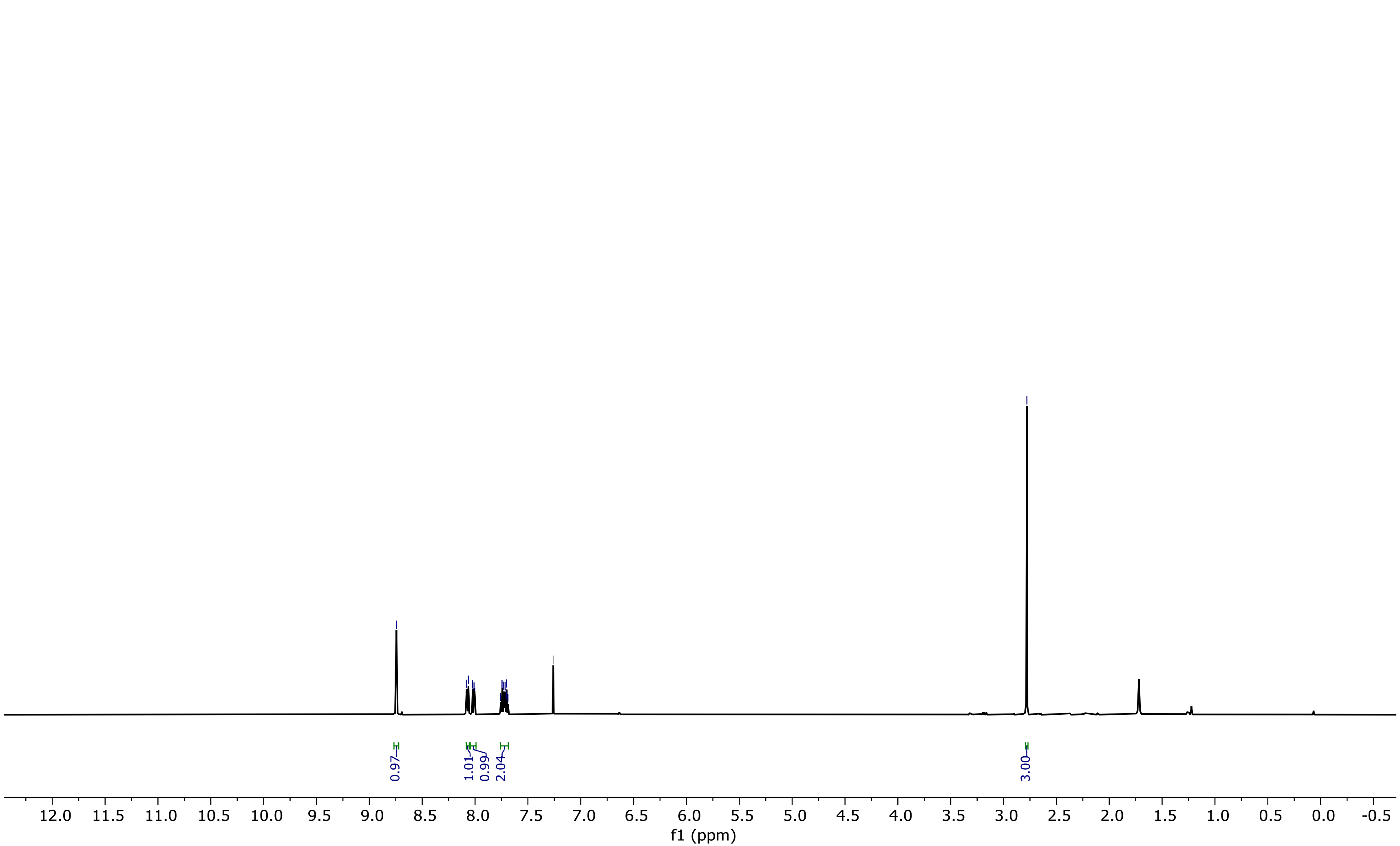

^{13}C NMR of 2-methylquinoxaline (4)

$CDCl_3$, 23 °C

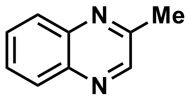

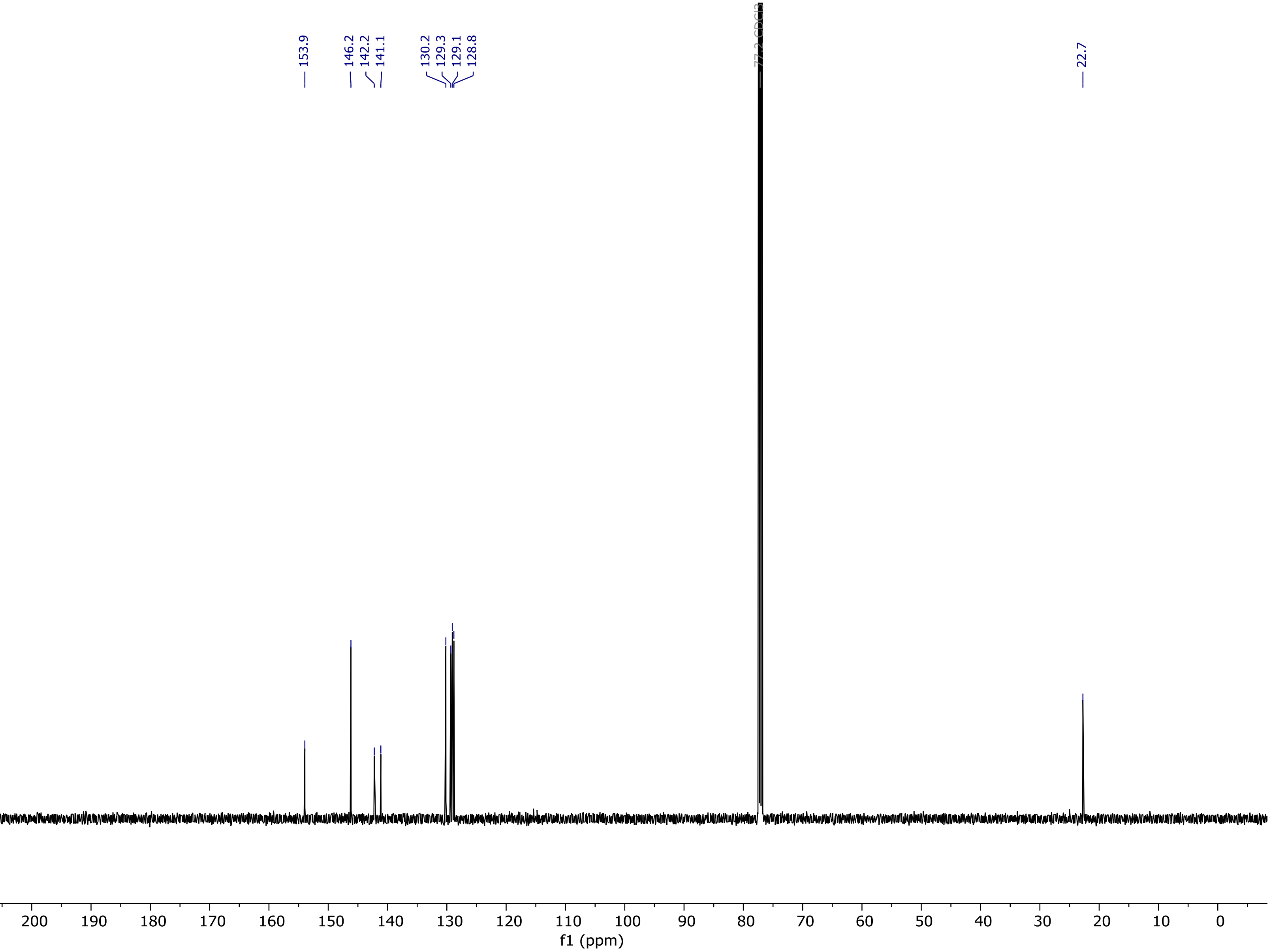

^{1}H NMR of 3-methylbenzoxazine (5)

$CDCl_3$, 23 ºC

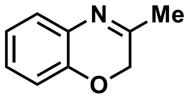

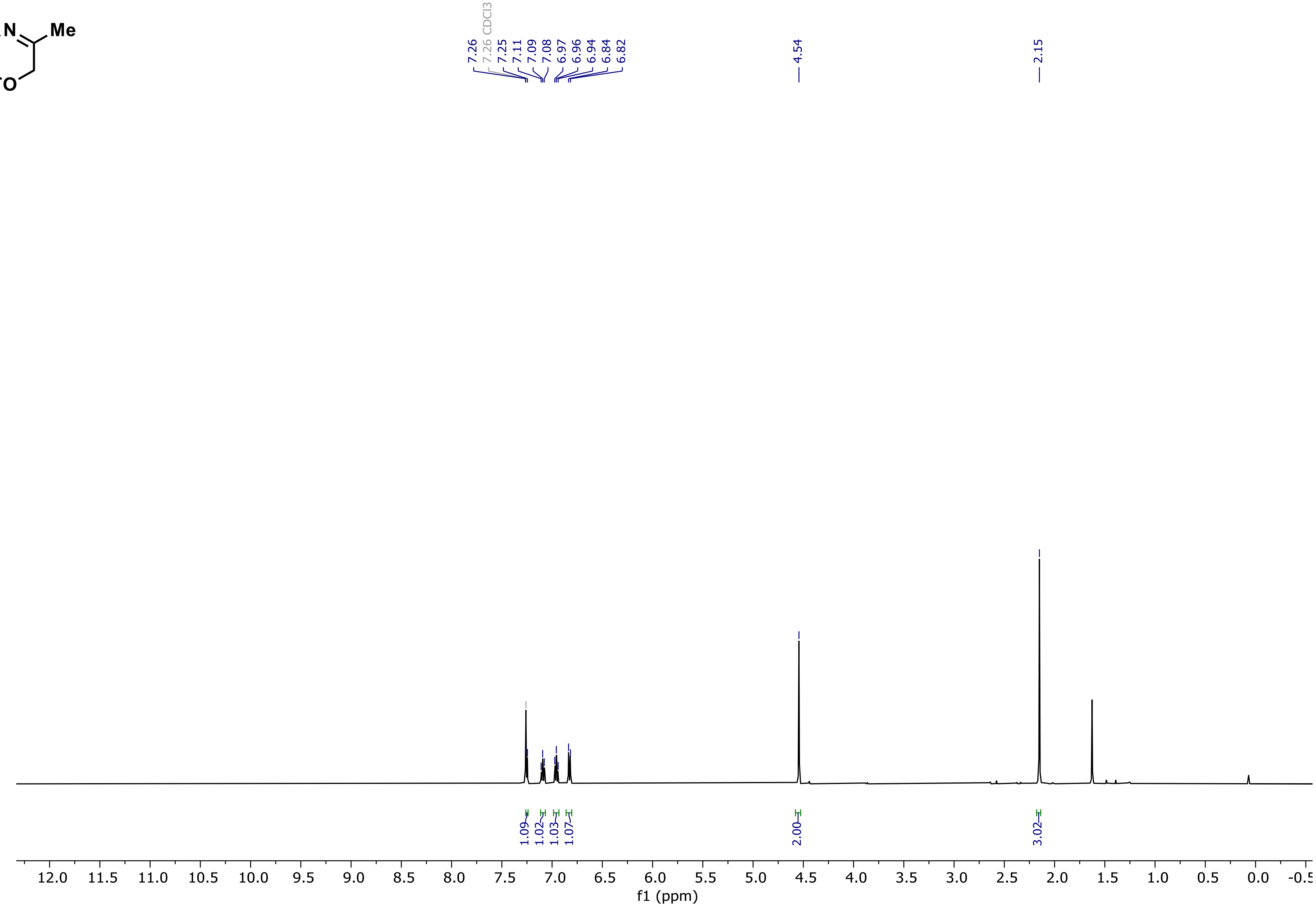

^{13}C NMR of 3-methylbenzoxazine (5)

$CDCl_3$, 23 ºC

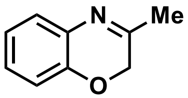

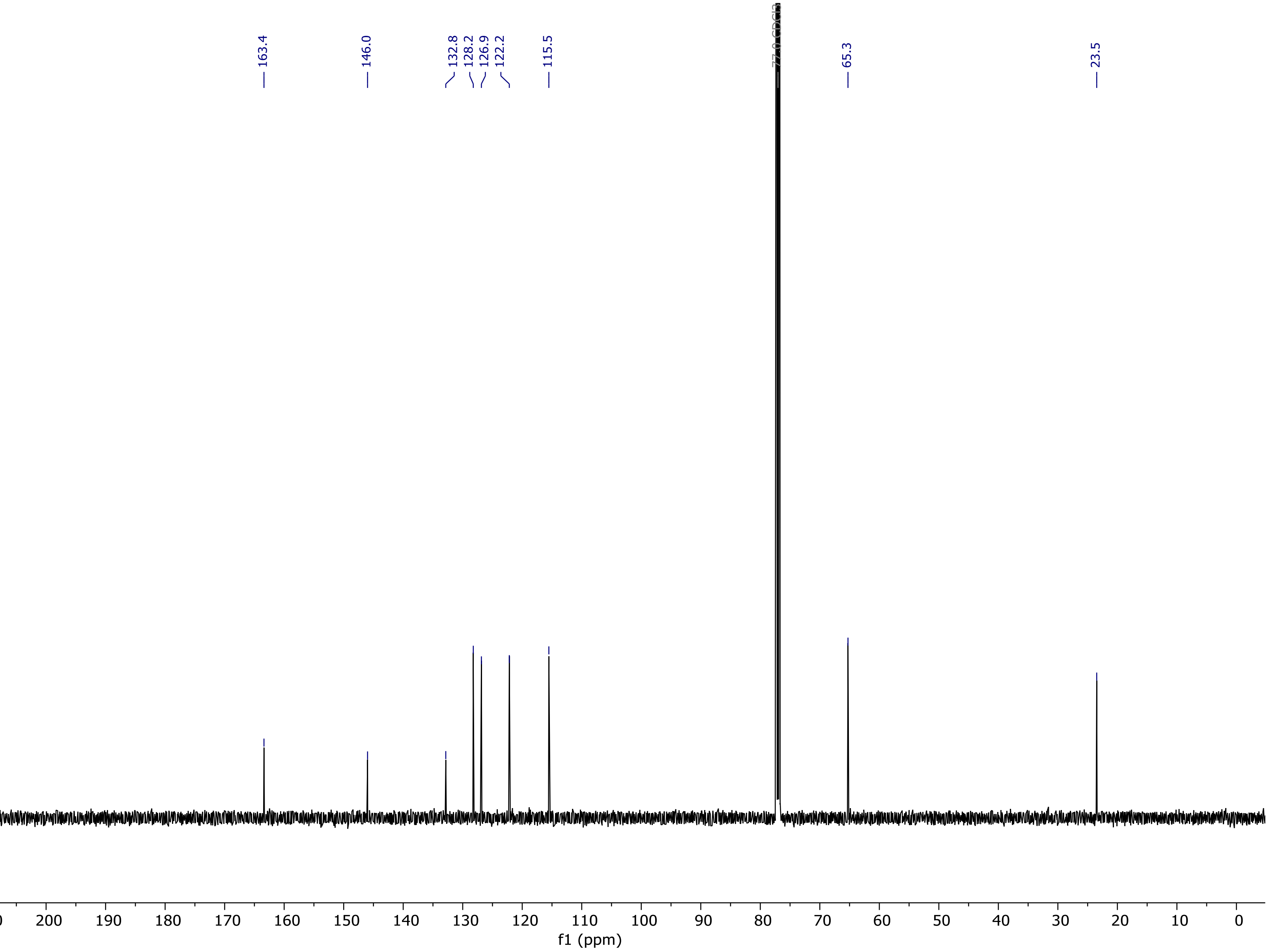

HMBC of 3-methylbenzoxazine (5)

$CDCl_3$, 23 ºC

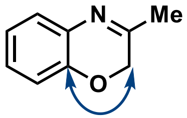

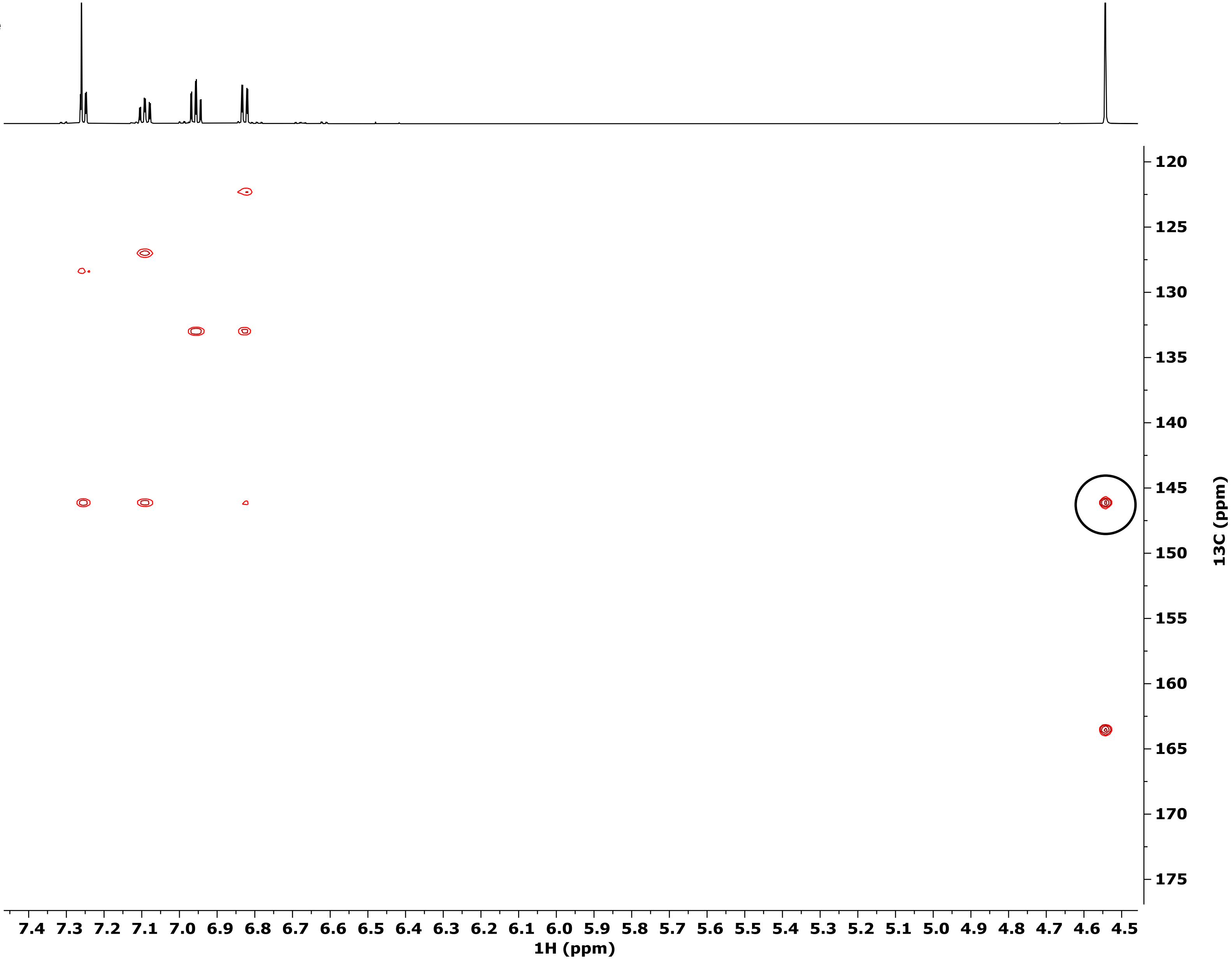

^{1}H NMR of benzodioxepinone derivative 6

$CDCl_3$, 23 ºC

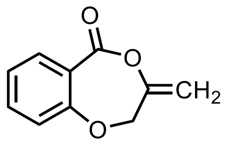

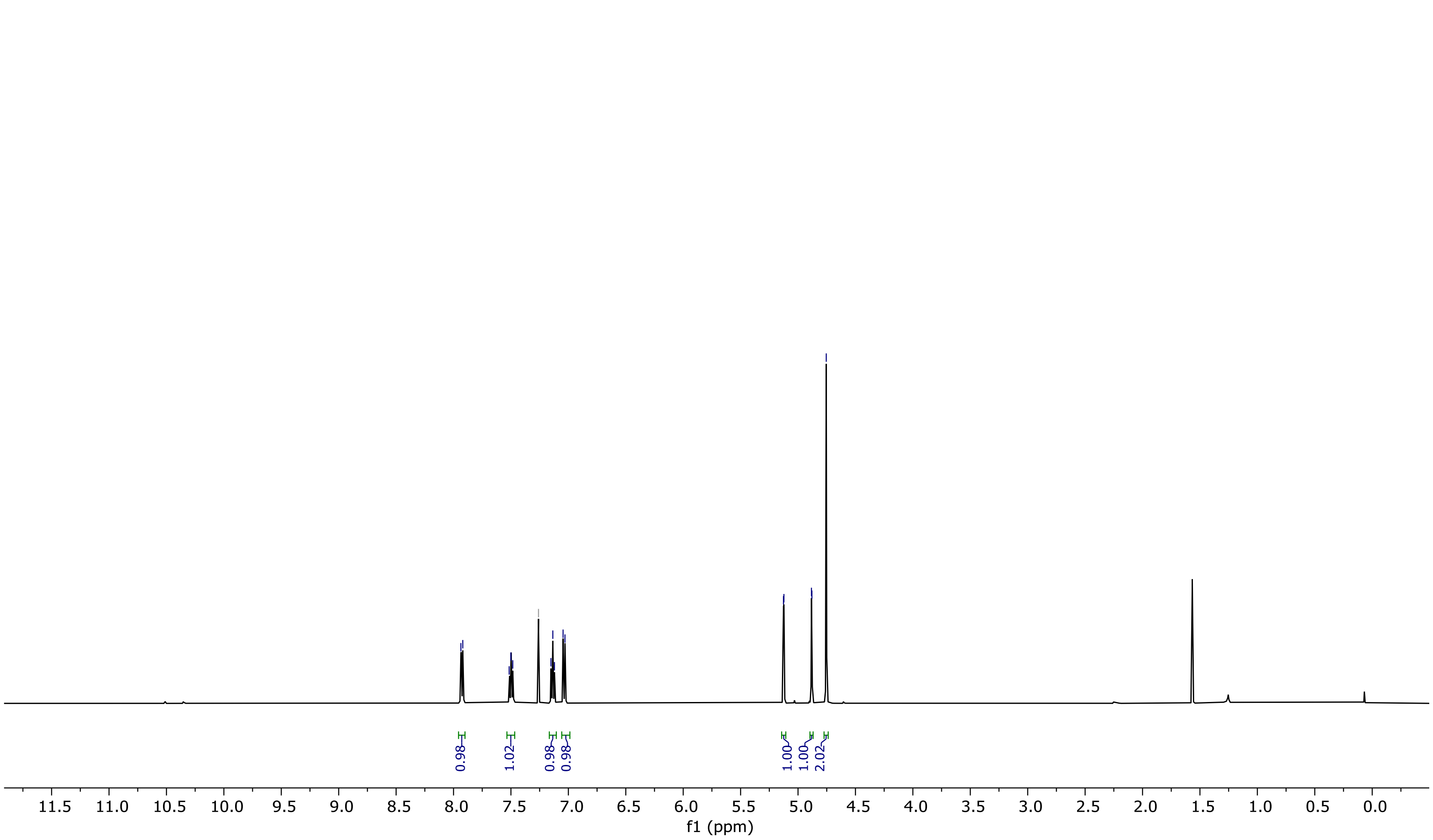

^{13}C NMR of benzodioxepinone derivative 6

$CDCl_3$, 23 ºC

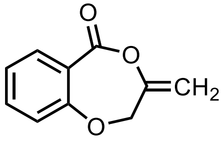

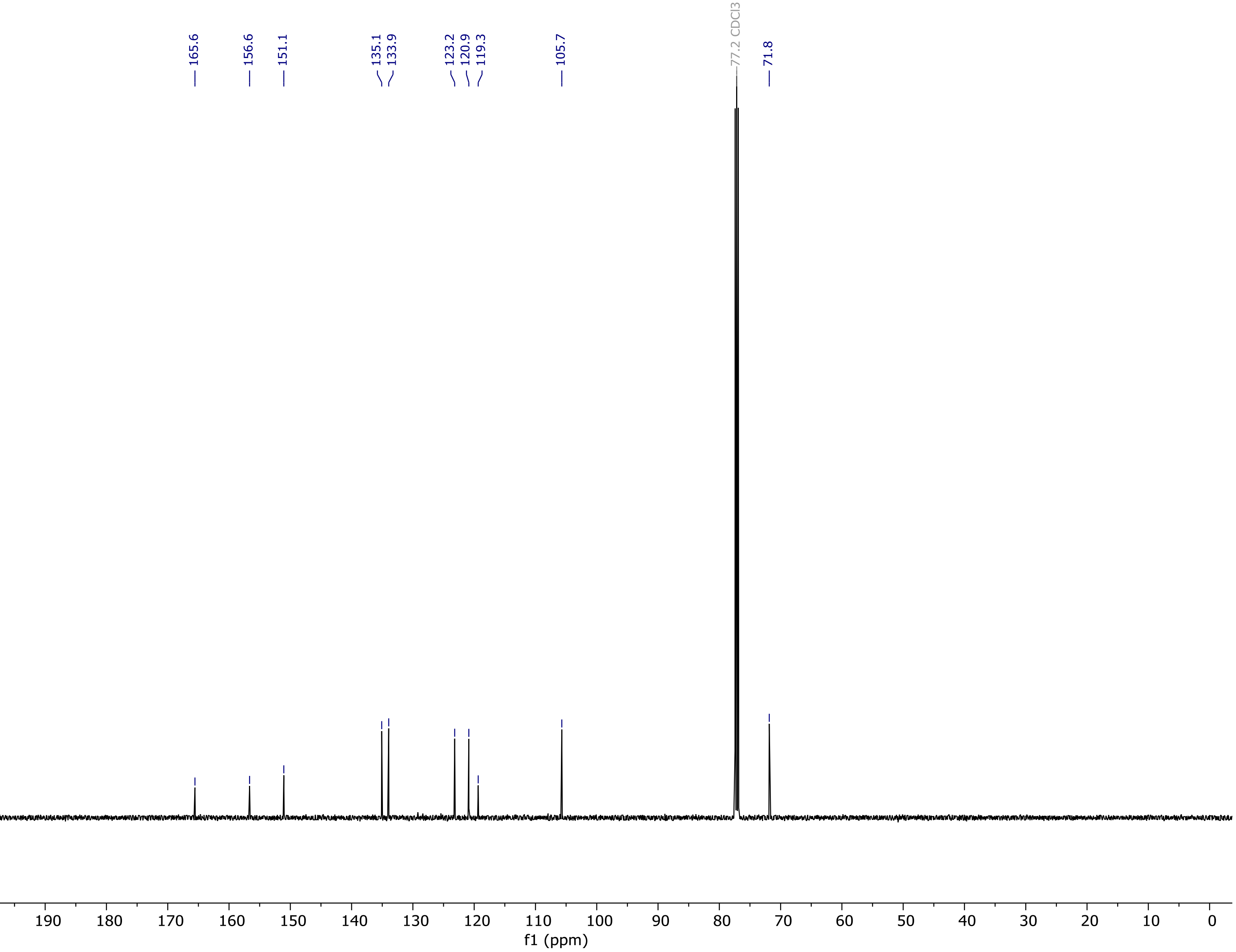

HMBC of benzodioxepinone derivative 6

$CDCl_3$, 23 ºC

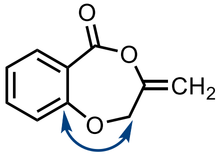

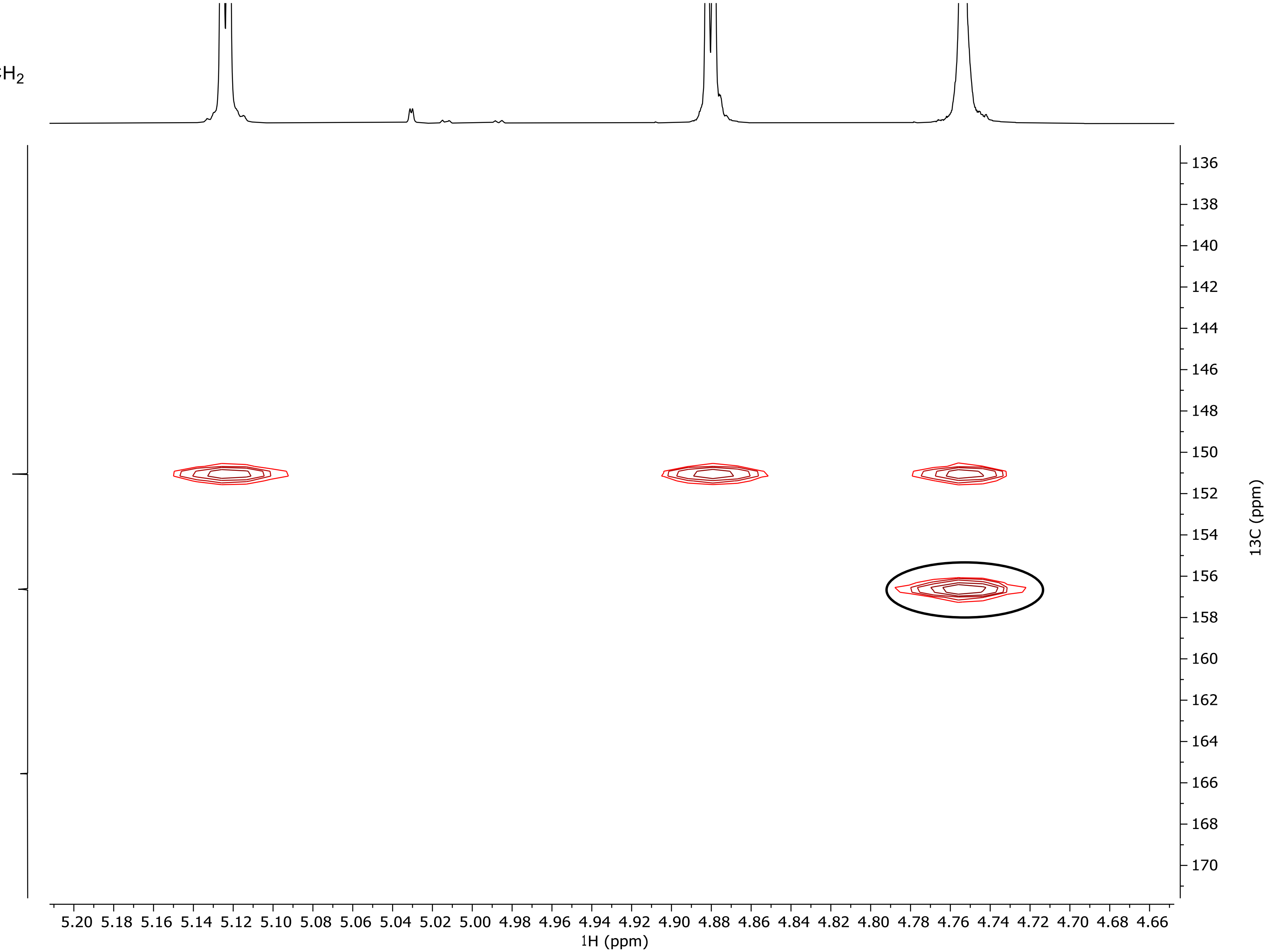

^{1}H NMR of benzoxathiepinone derivative 7

$CDCl_3$, 23 ºC

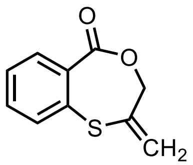

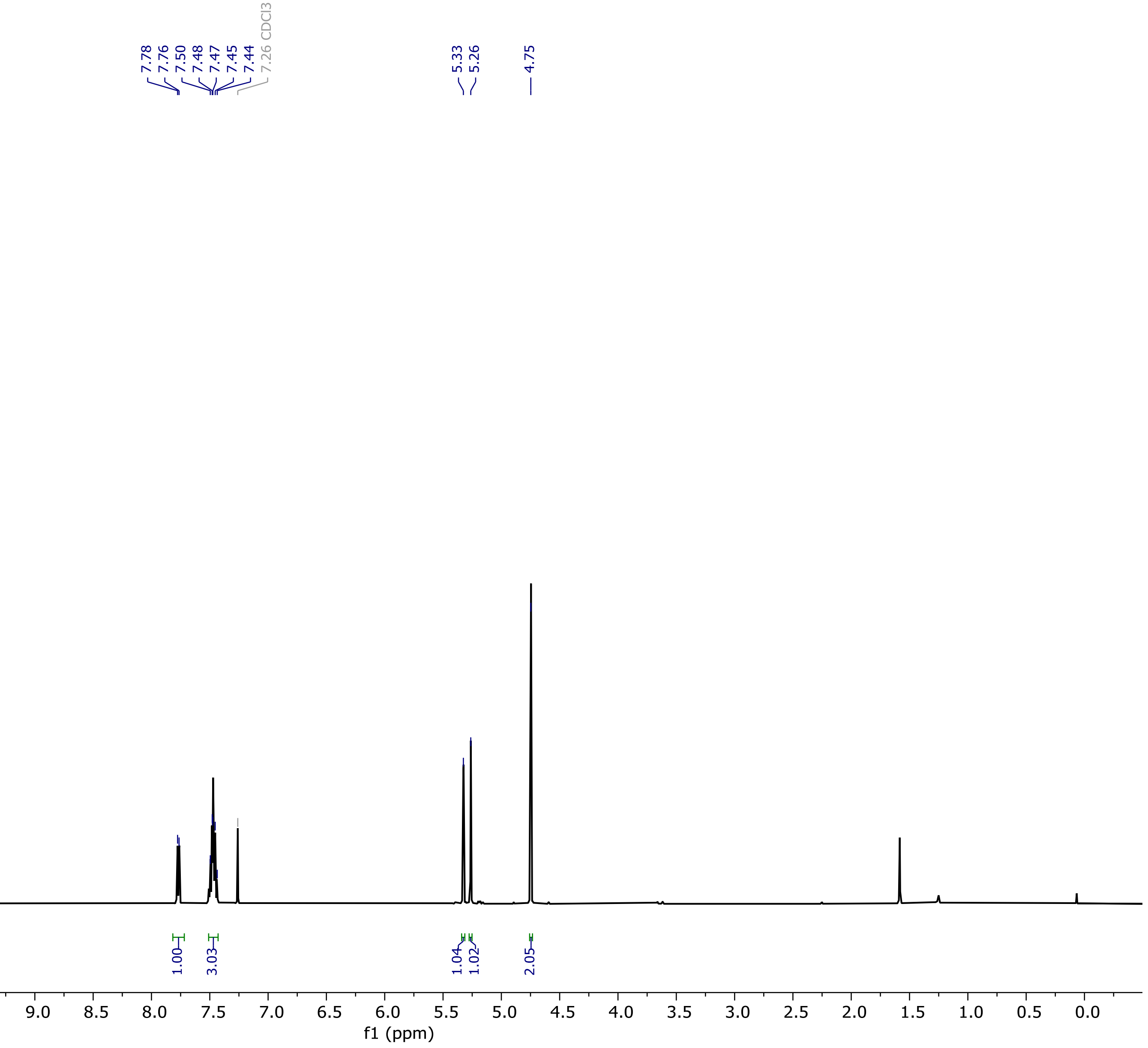

^{13}C NMR of benzoxathiepinone derivative 7

$CDCl_3$, 23 ºC

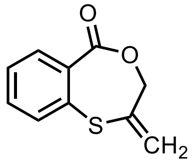

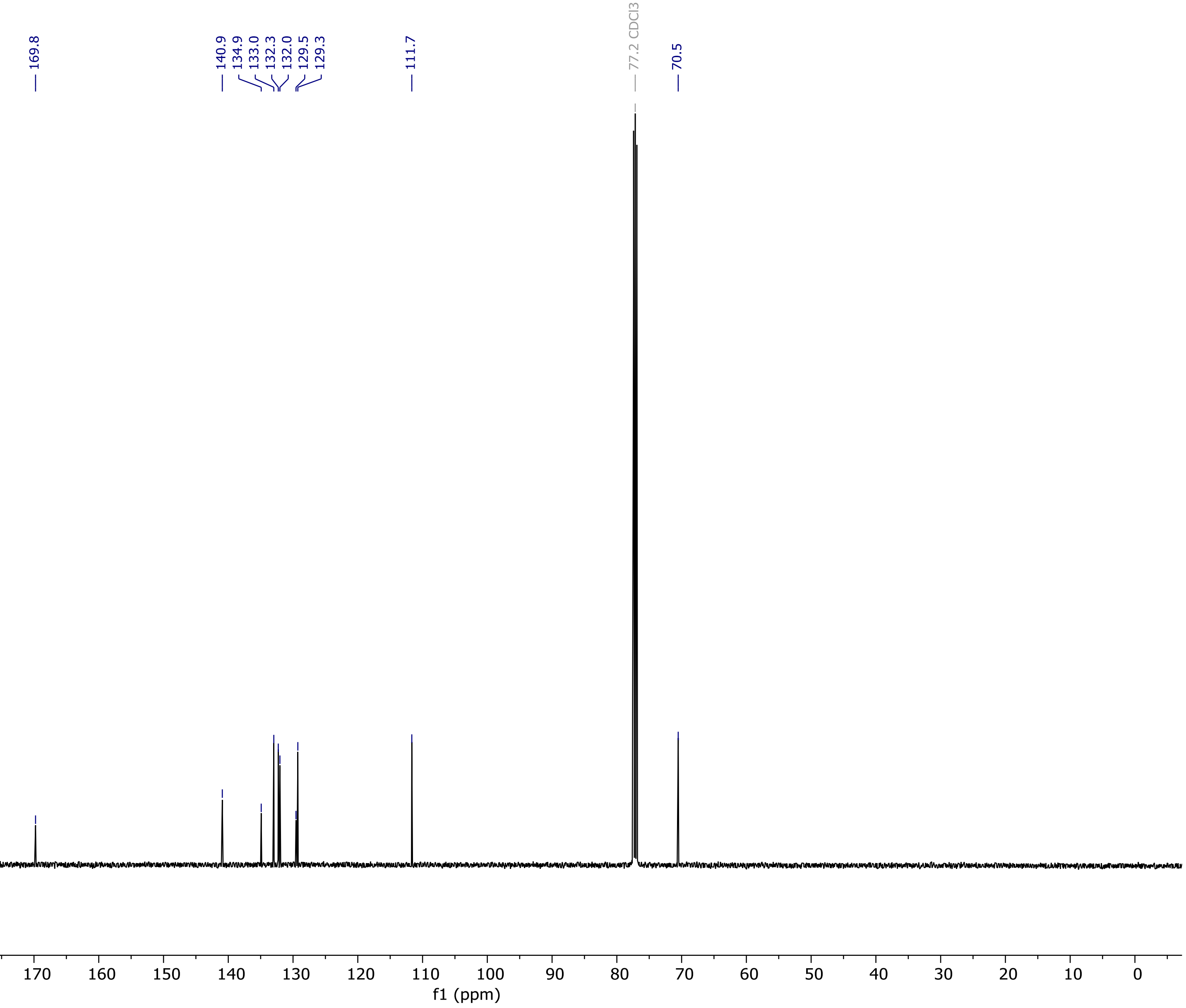

HMBC of benzoxathiepinone derivative 7

$CDCl_3$, 23 ºC

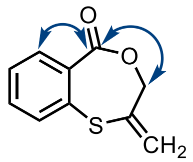

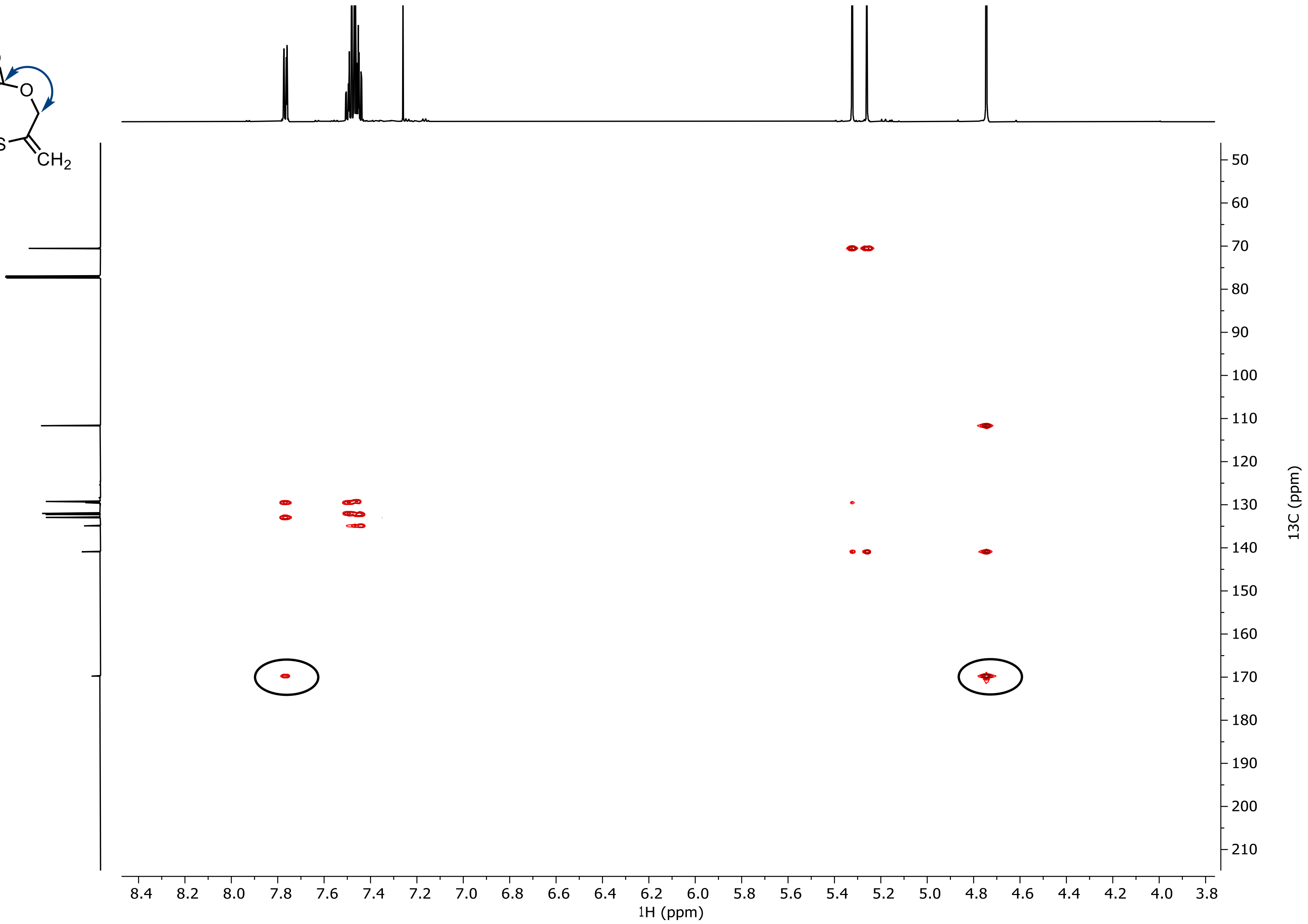

^{1}H NMR of diflunisal derivative 8

$CDCl_3$, 23 ºC

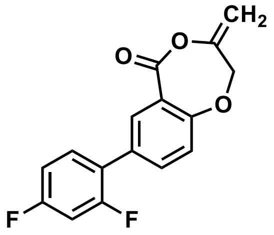

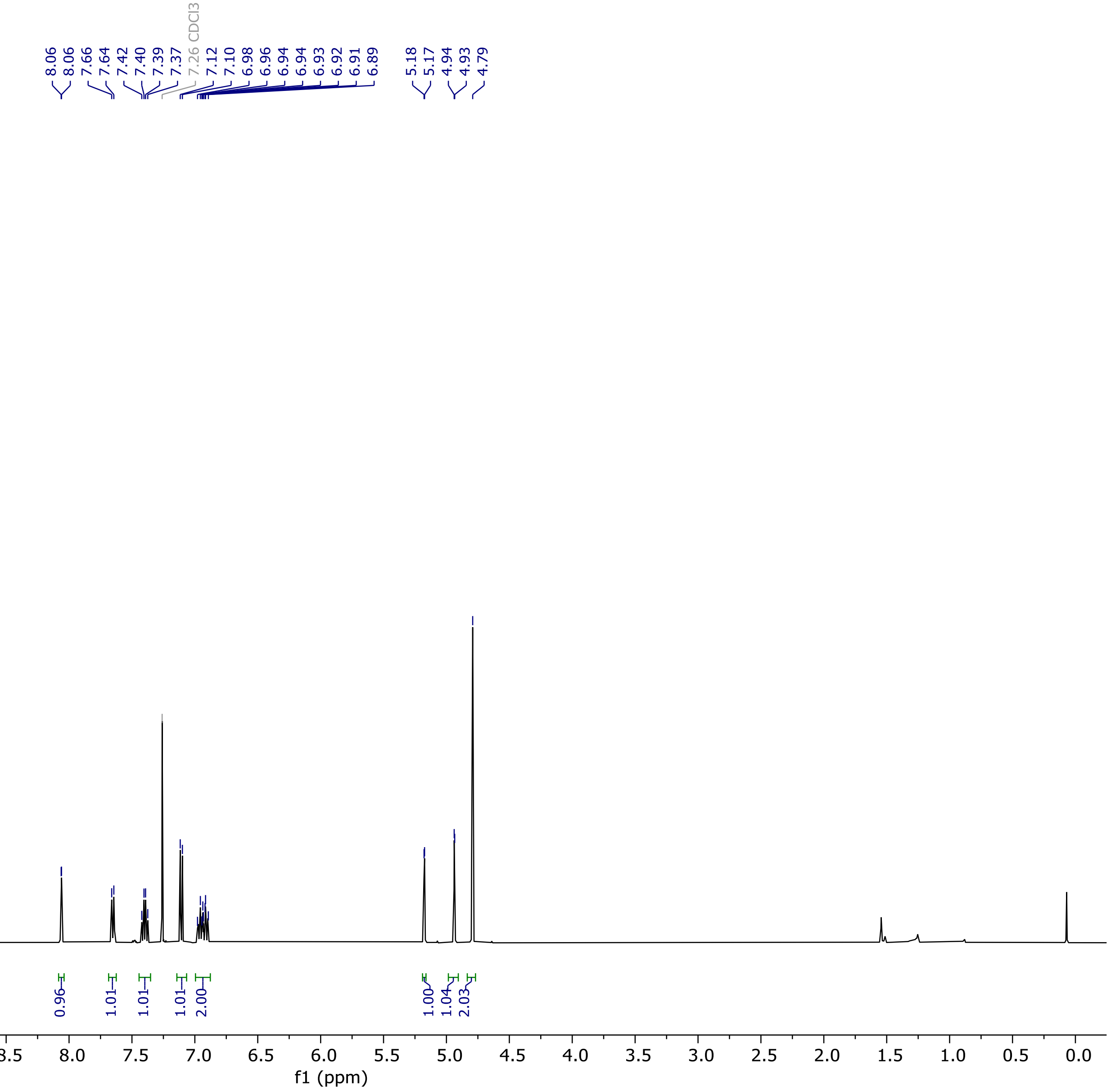

^{13}C NMR of diflunisal derivative 8

$CDCl_3$, 23 ºC

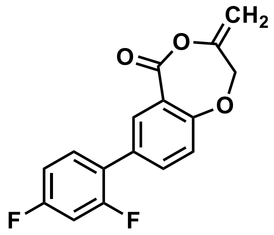

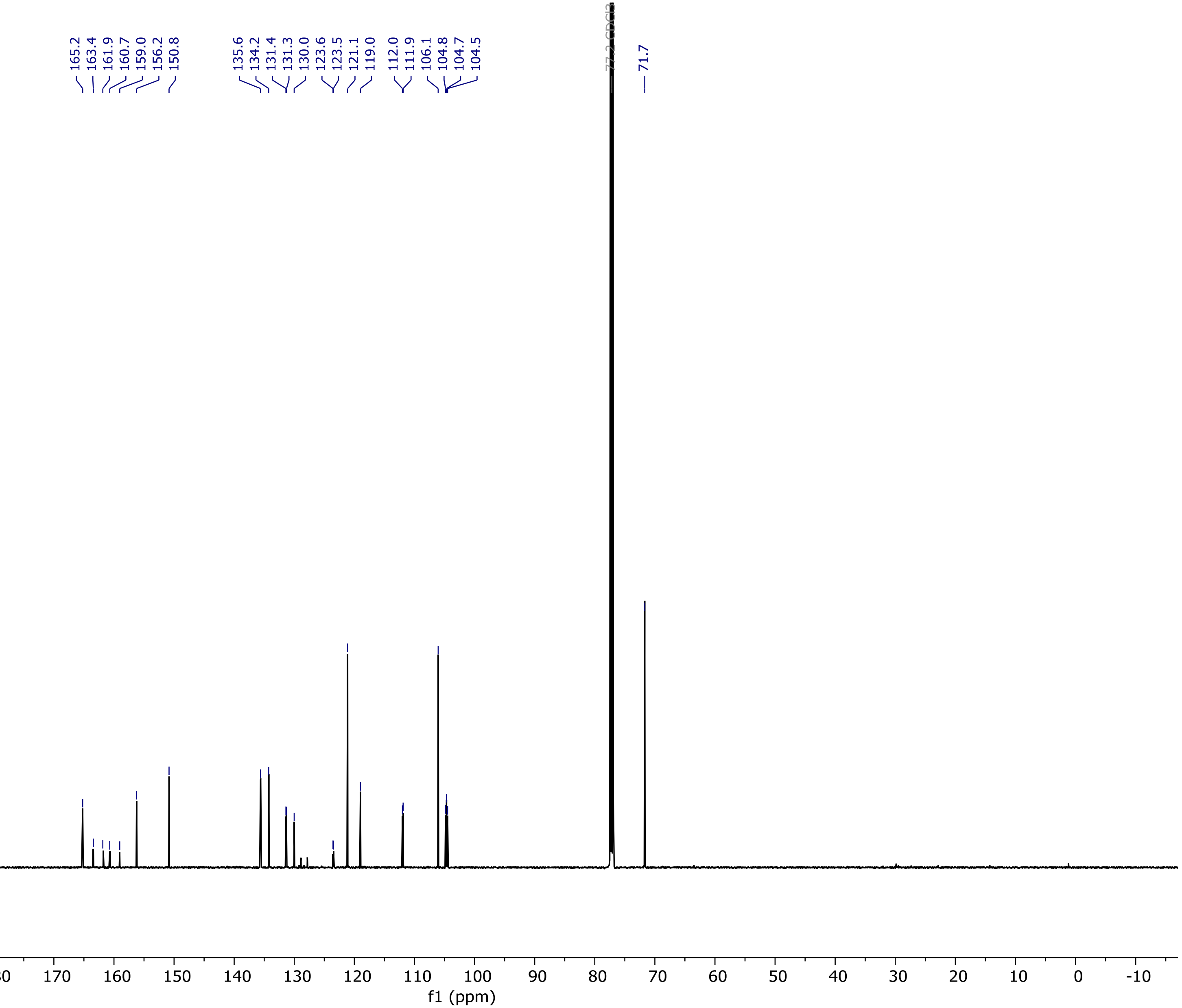

^{19}F NMR of diflunisal derivative 8

$CDCl_3$, 23 ºC

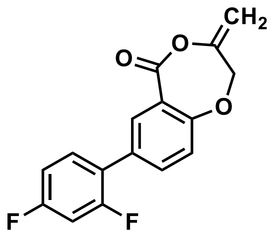

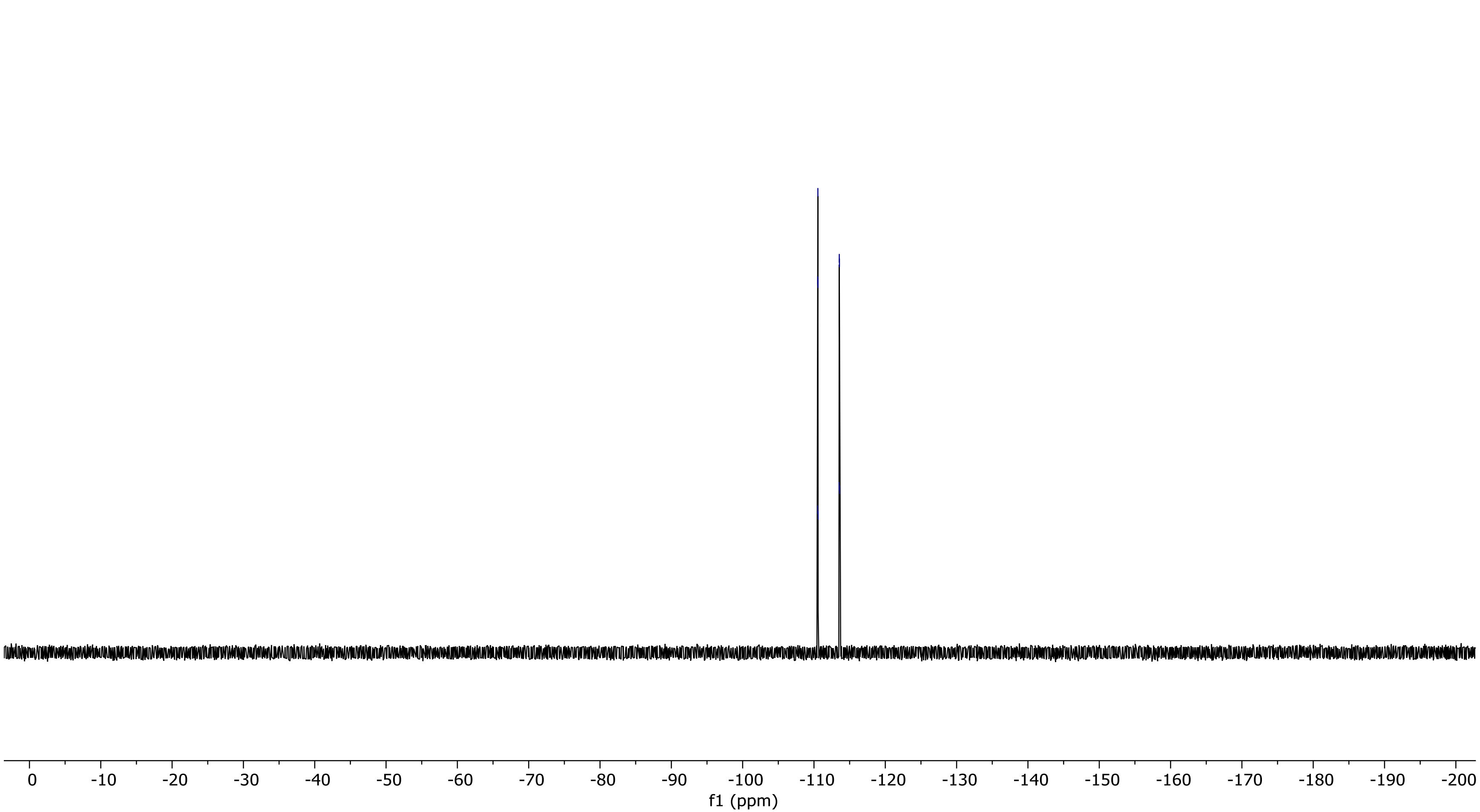

HMBC of diflunisal derivative 8

$CDCl_3$, 23 ºC

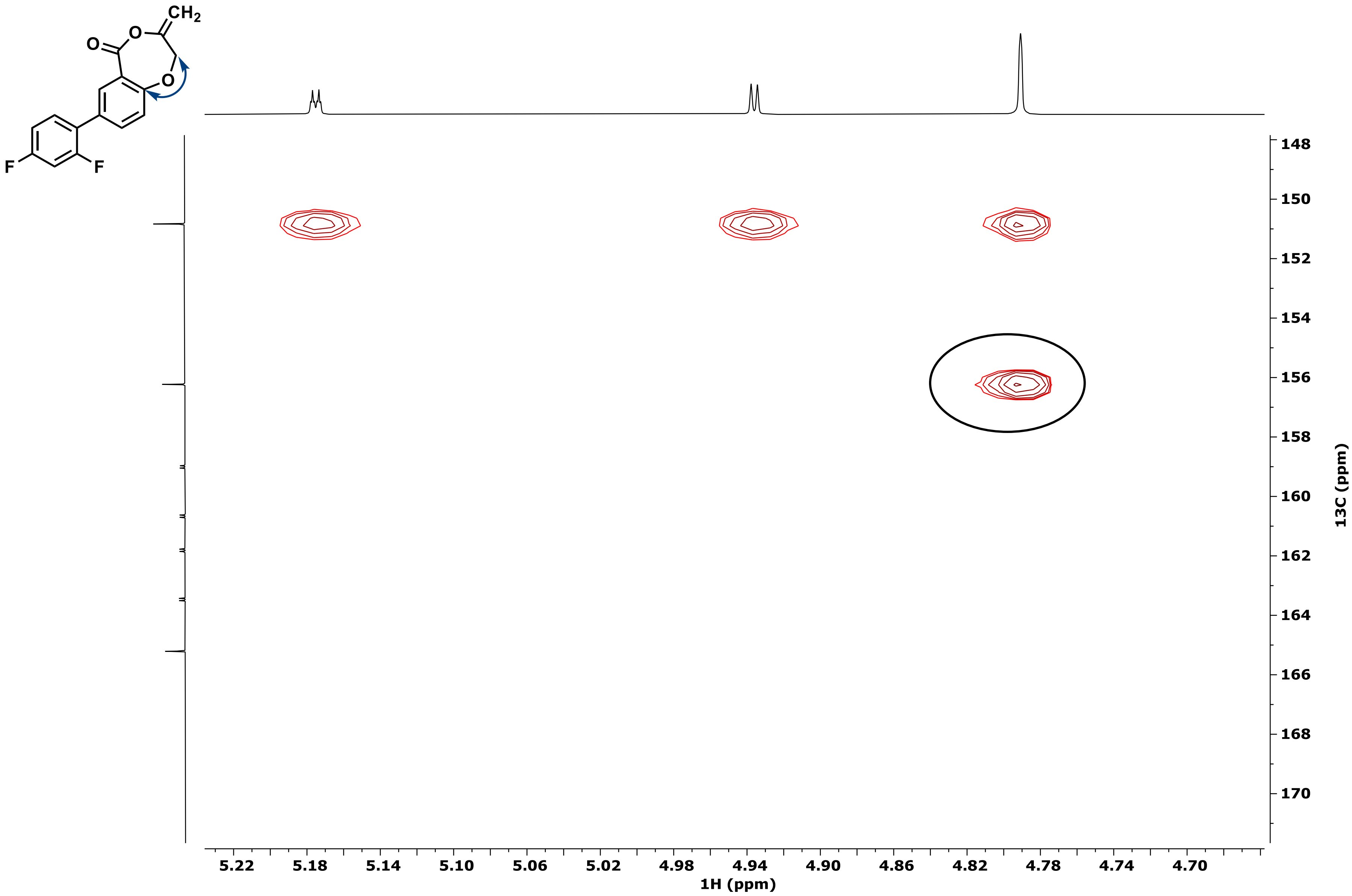

^{1}H NMR of (7,9-di-*tert*-butyl)benzodioxepinone derivative 9

$CDCl_3$, 23 ºC

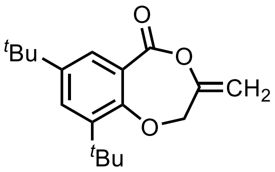

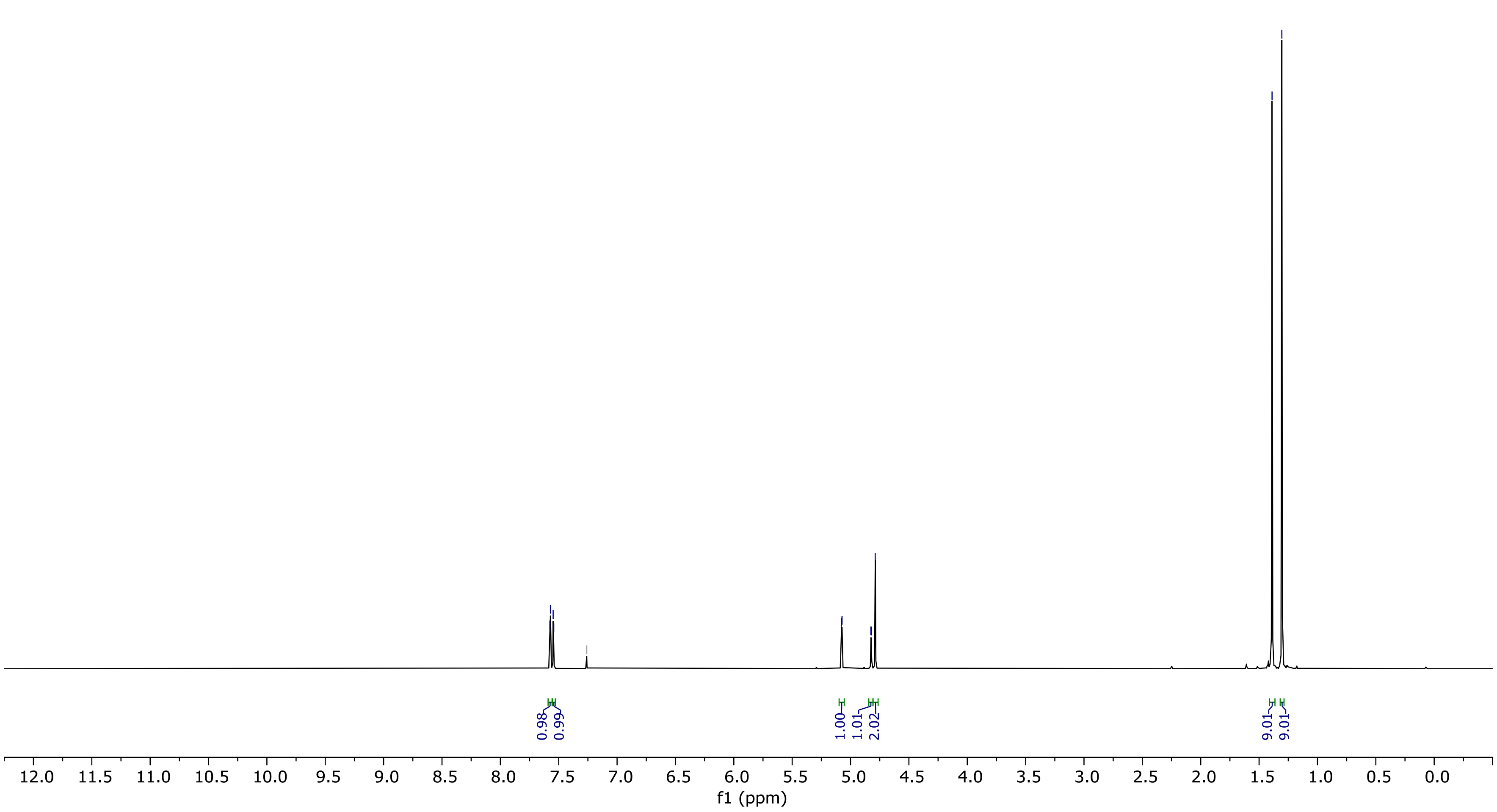

^{13}C NMR of (7,9-di-*tert*-butyl)benzodioxepinone derivative 9

$CDCl_3$, 23 ºC

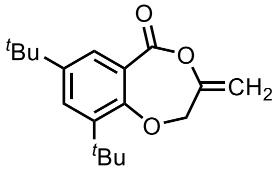

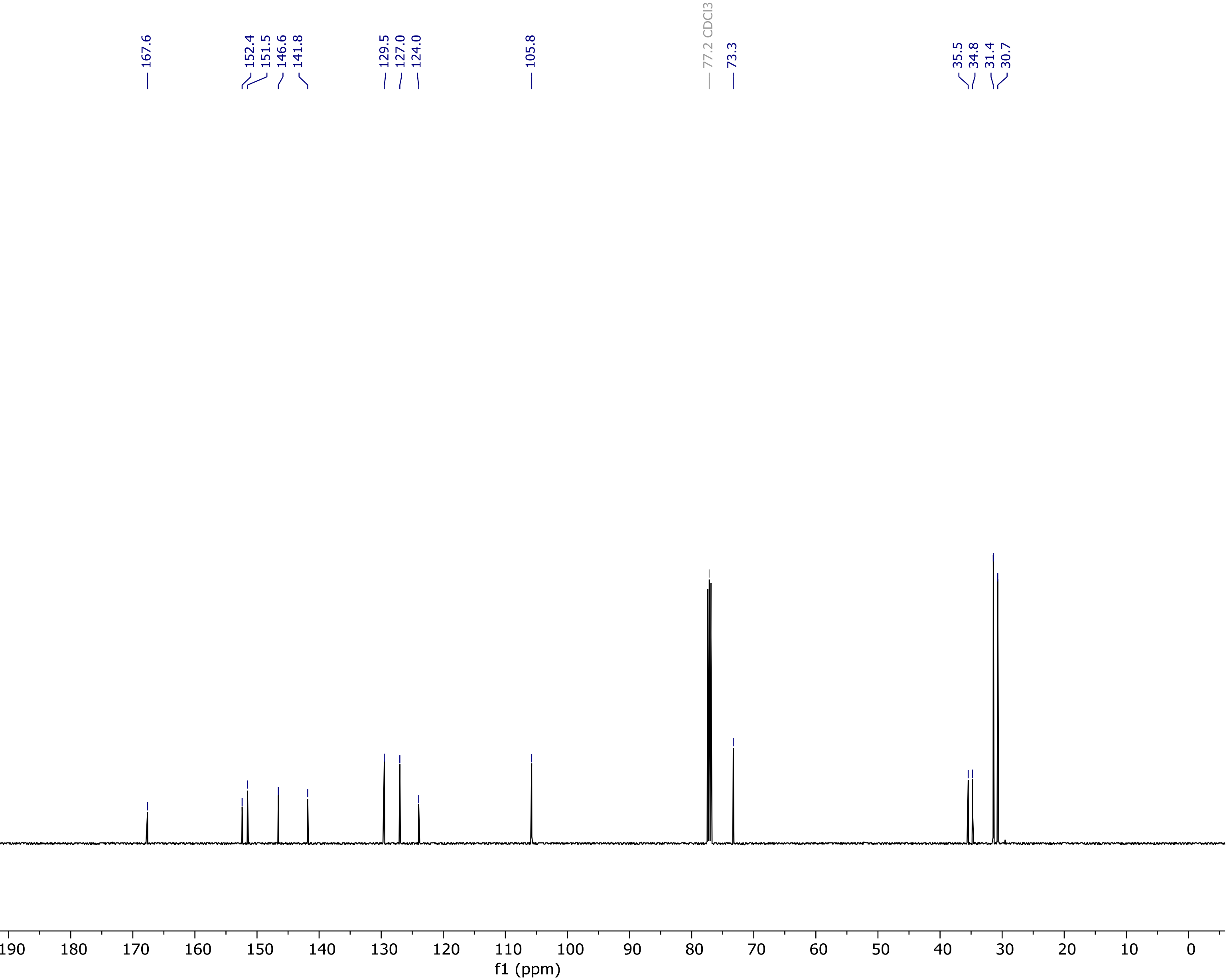

HMBC of (7,9-di-*tert*-butyl)benzodioxepinone derivative 9

$CDCl_3$, 23 ºC

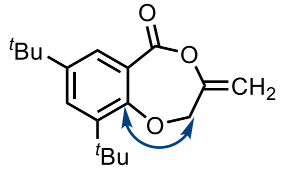

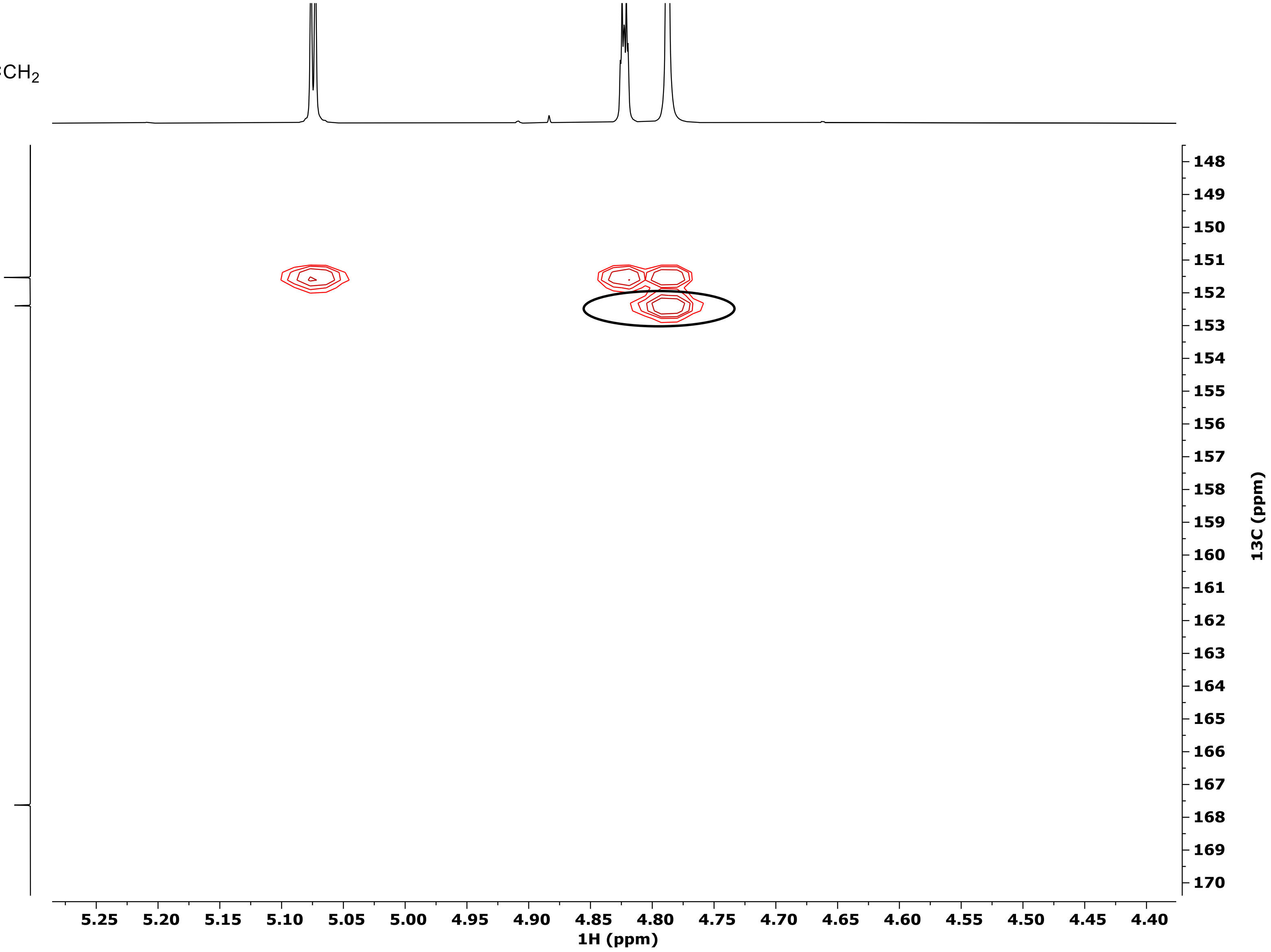

^{1}H NMR of benzodioxine derivative 10

$CDCl_3$, 23 ºC

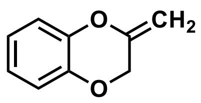

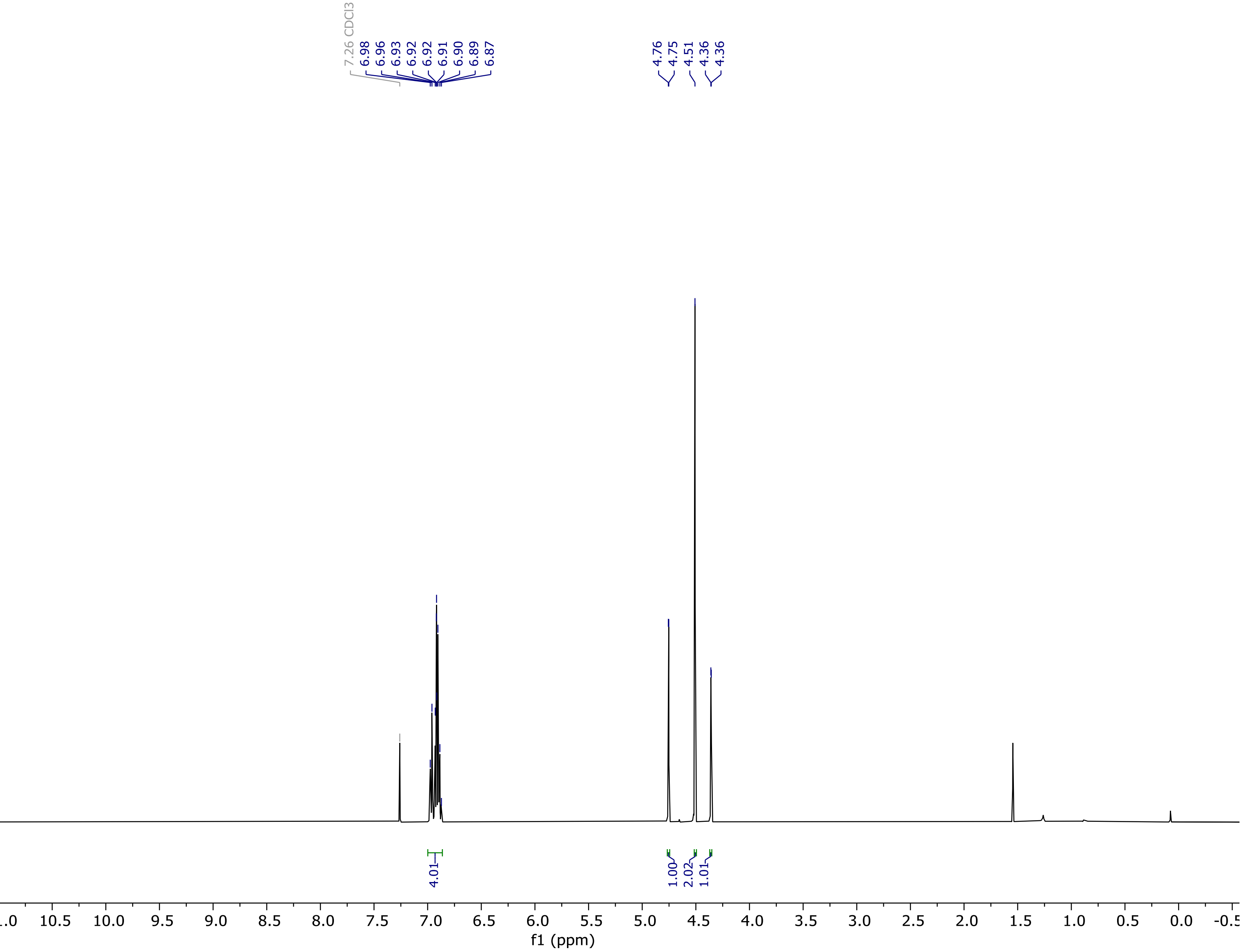

^{13}C NMR of benzodioxine derivative 10

$CDCl_3$, 23 ºC

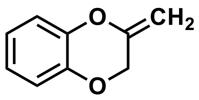

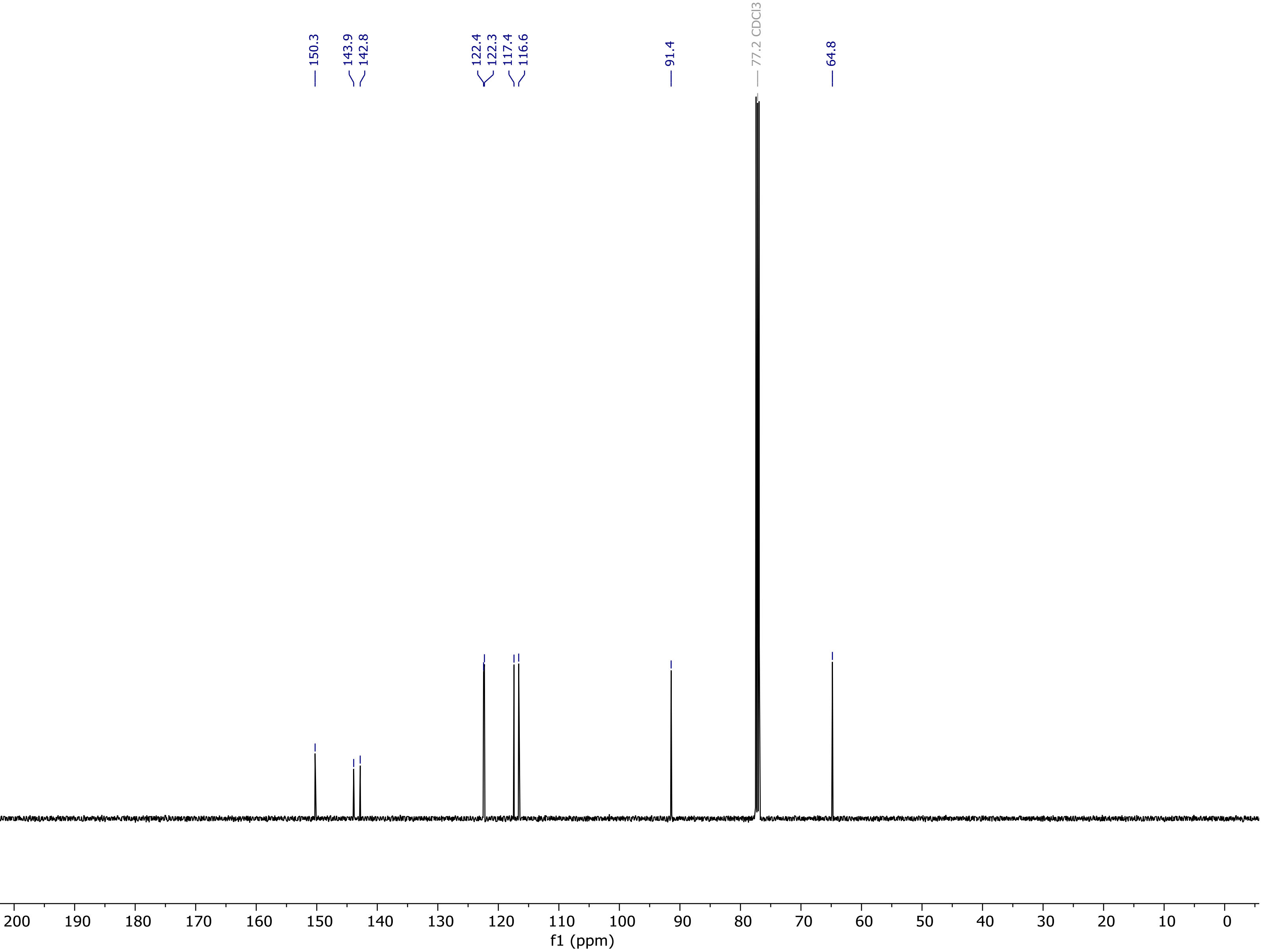

^{1}H NMR of pseudoephedrine derivative 11

CDCl$_3$, 23 ºC

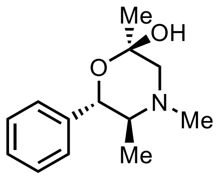

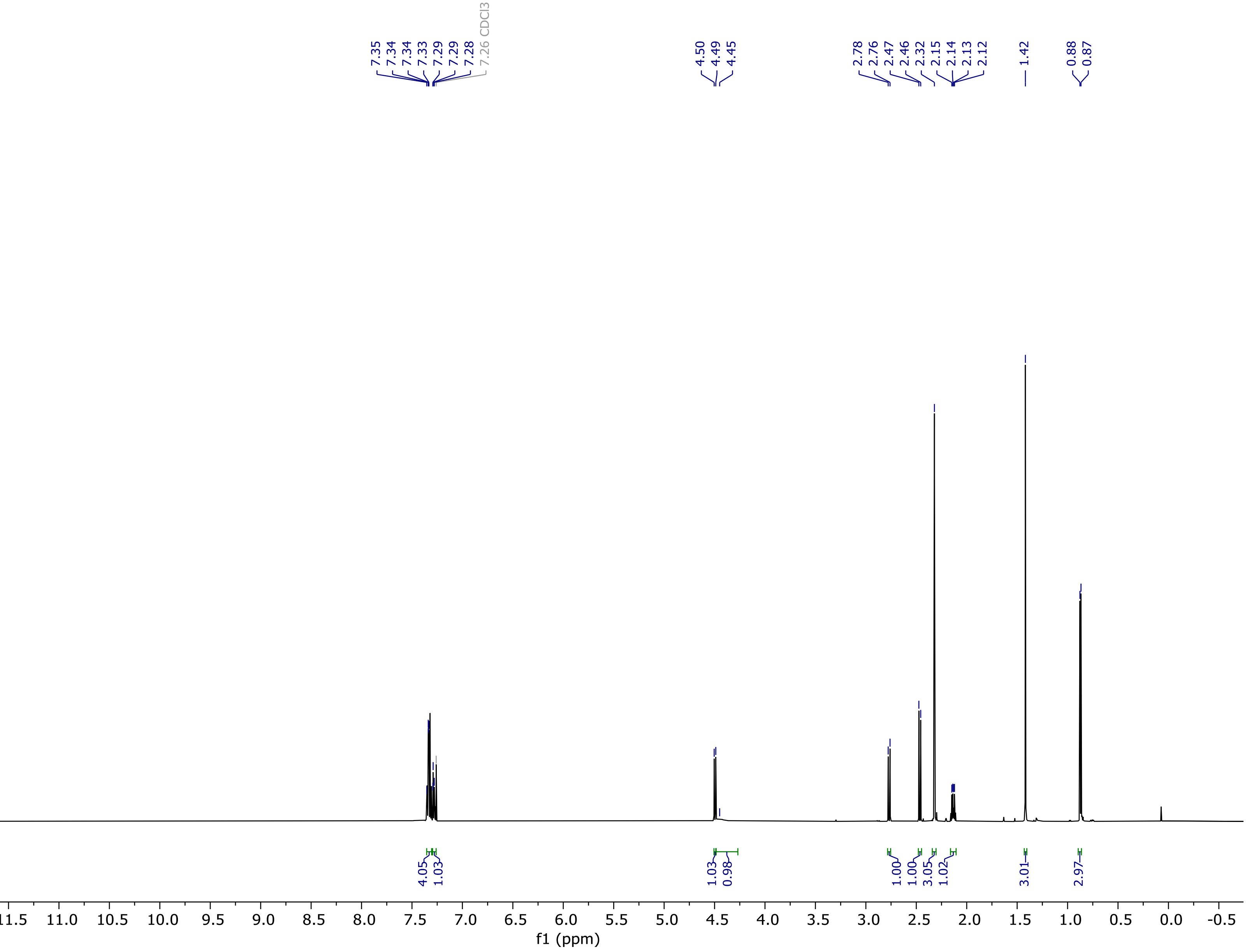

^{13}C NMR of pseudoephedrine derivative 11

$CDCl_3$, 23 ºC

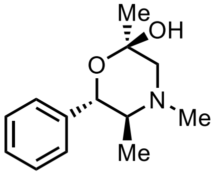

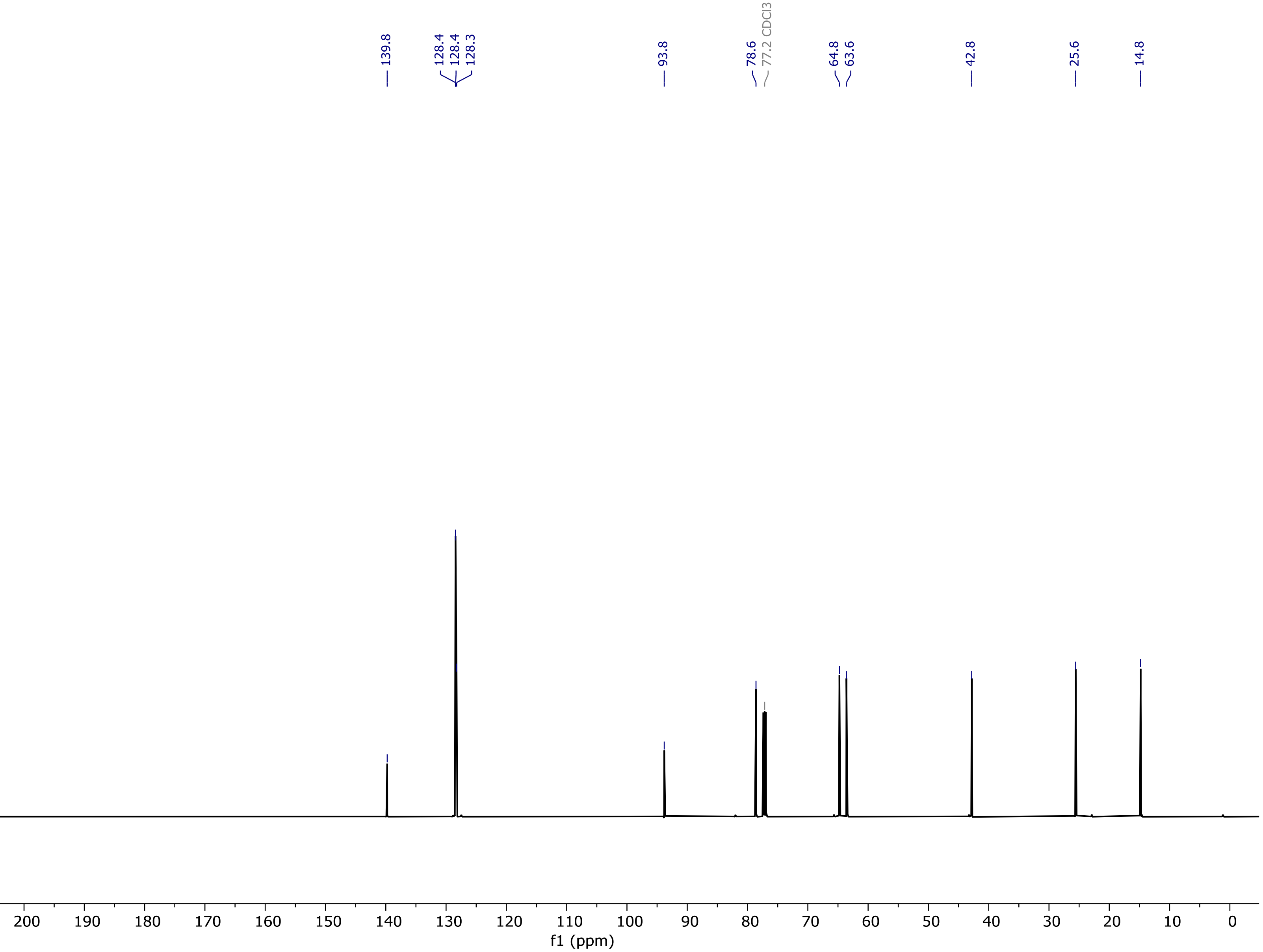

NOESY of pseudoephedrine derivative 11

$CDCl_3$, 23 ºC

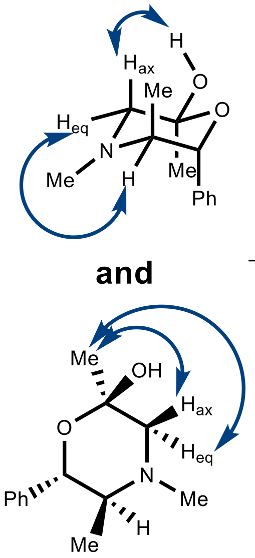

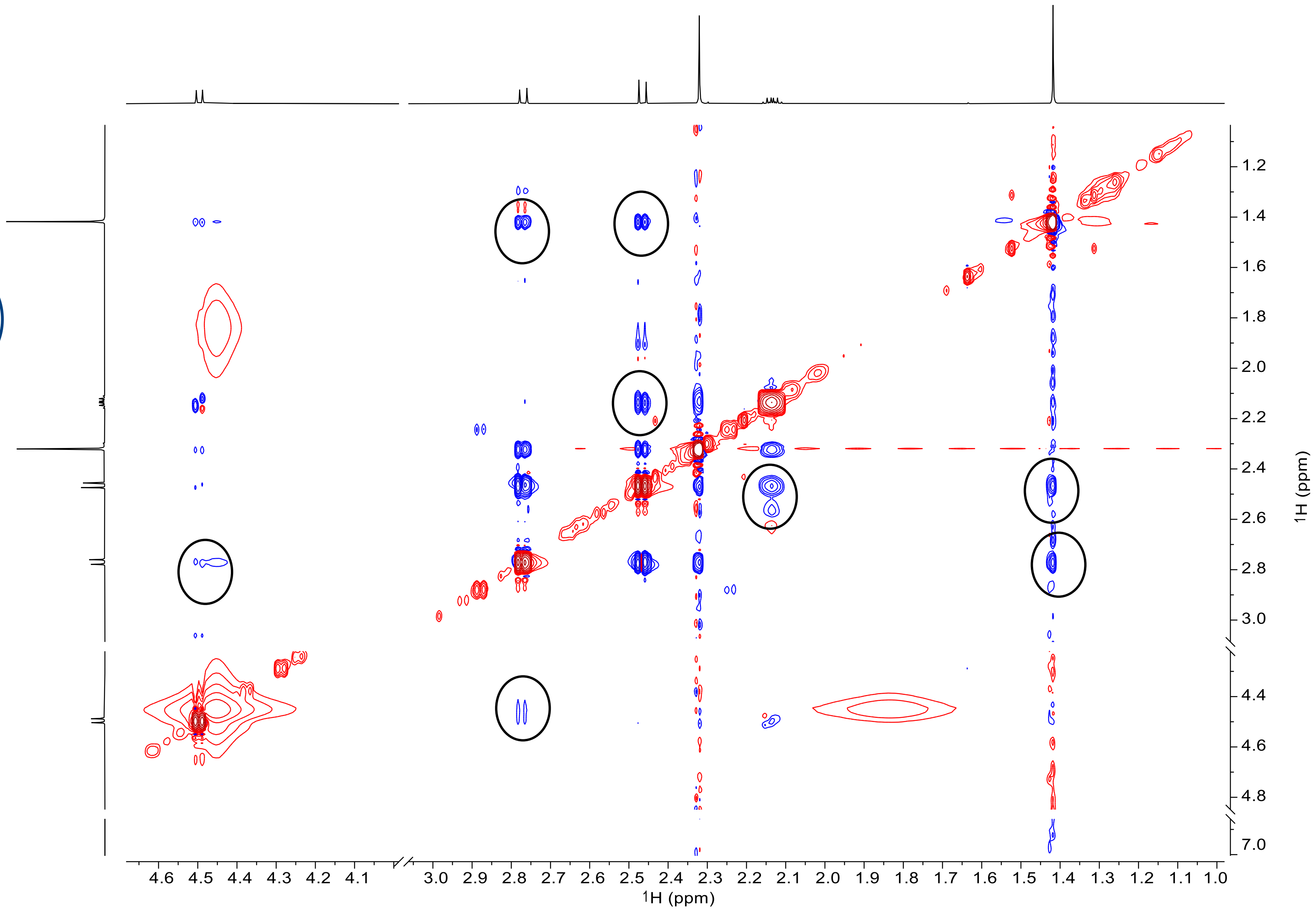

HMBC of pseudoephedrine derivative 11

$CDCl_3$, 23 ºC

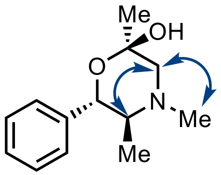

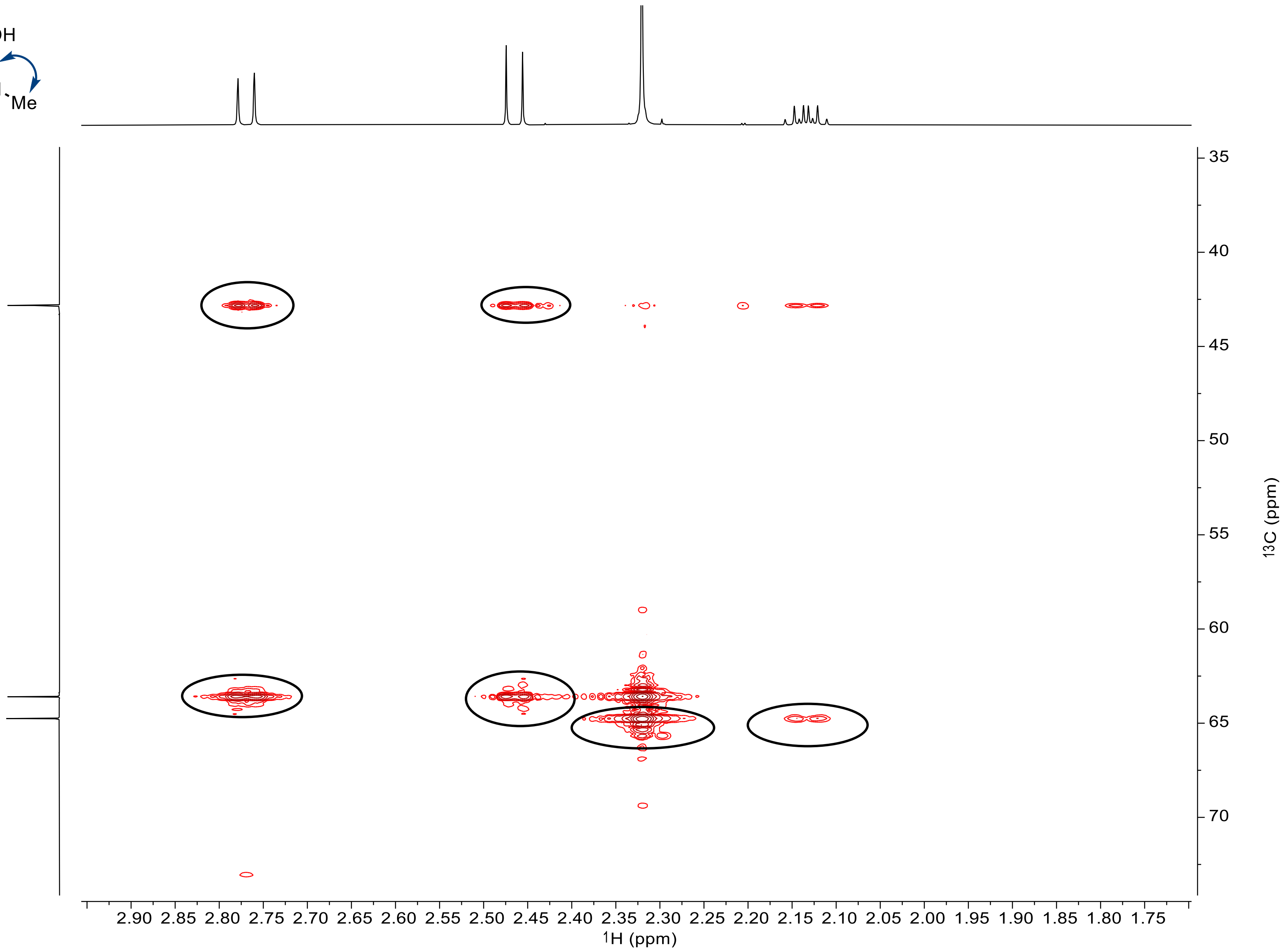

^{1}H NMR of allenyl-4-phenylbenzene (12)

$CDCl_3$, 23 ºC

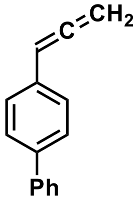

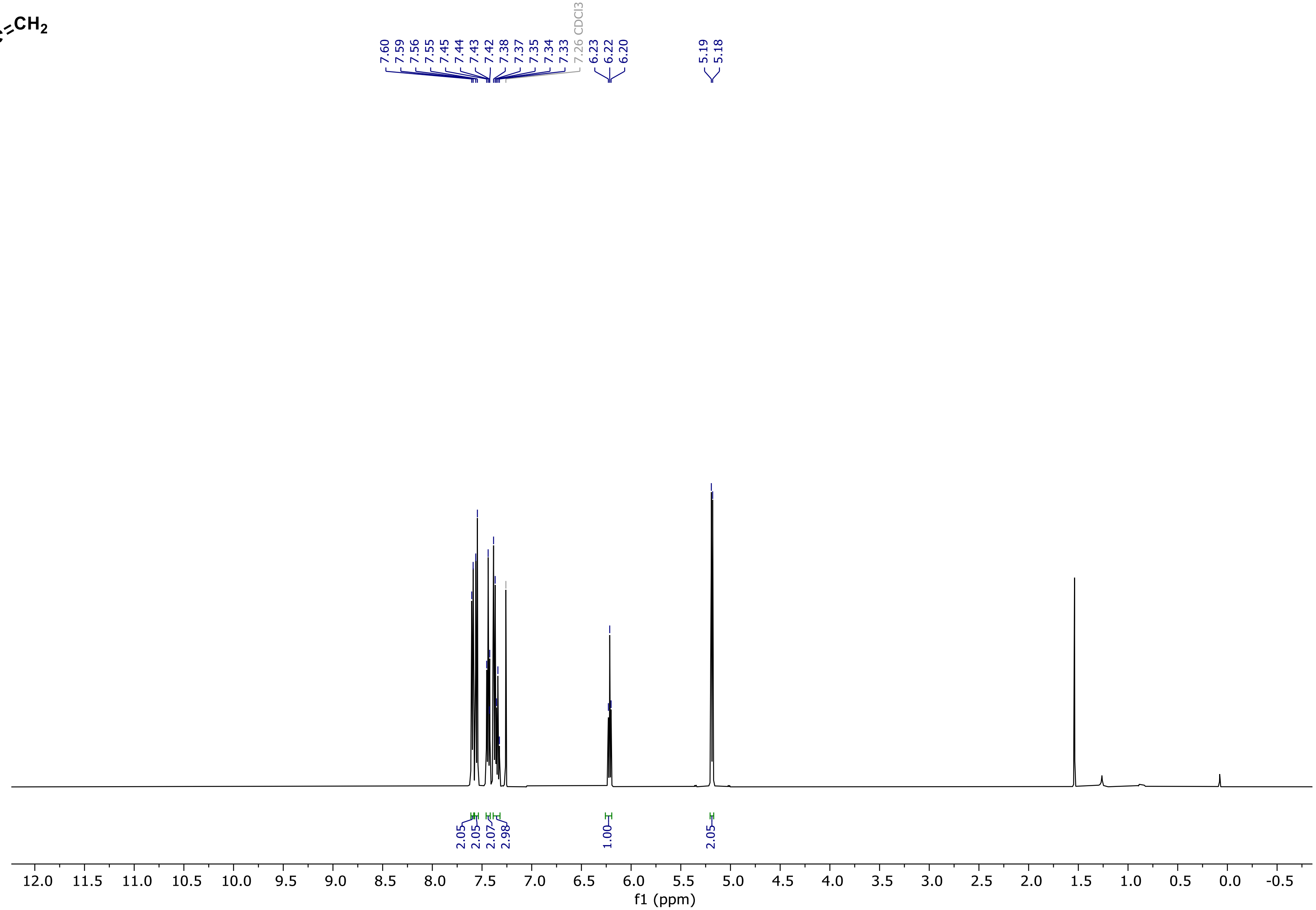

^{13}C NMR of allenyl-4-phenylbenzene (12)

$CDCl_3$, 23 ºC

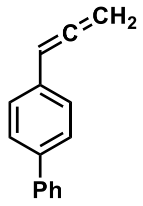

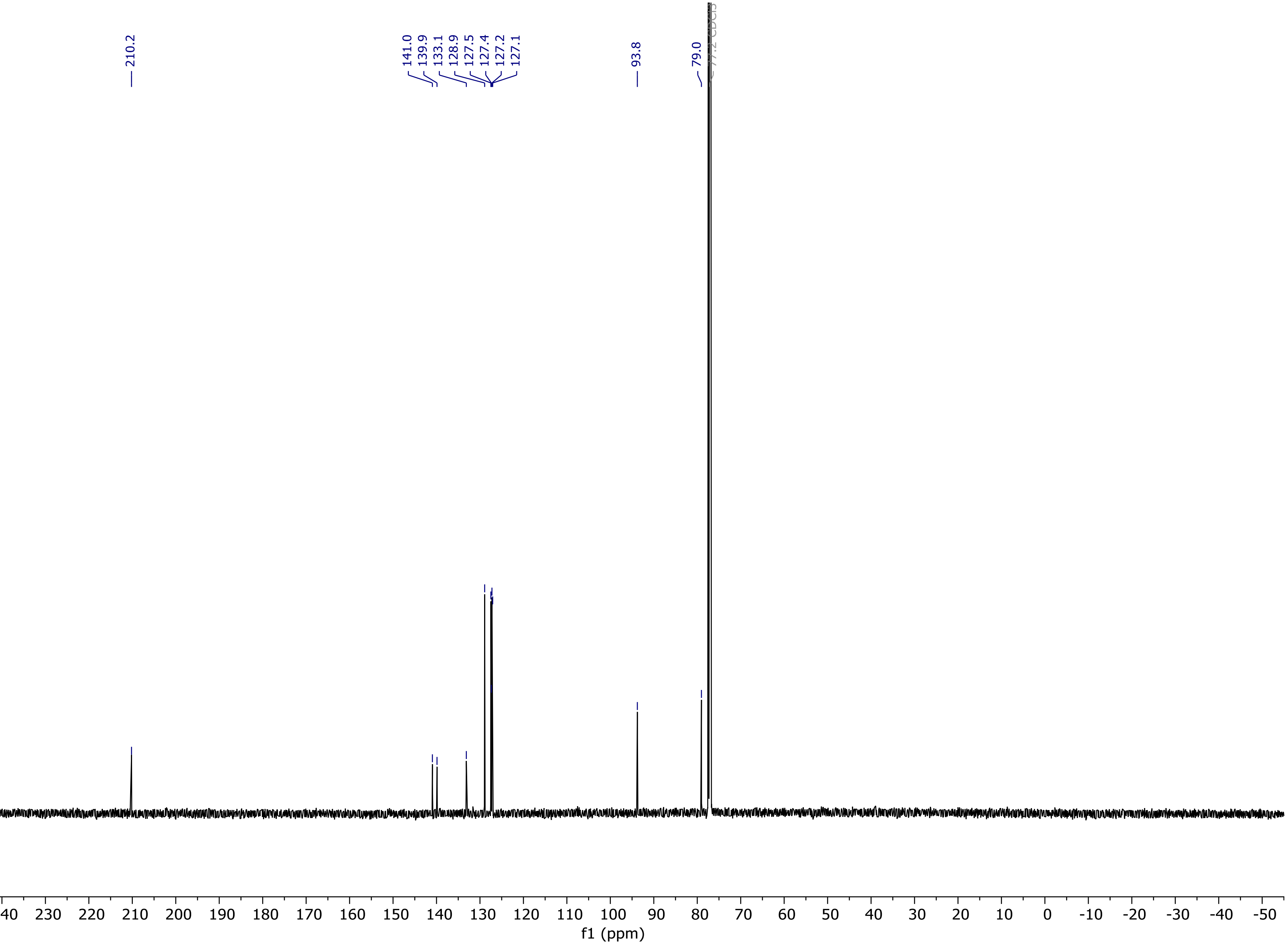

^{1}H NMR of allenyl-(2,4,6-trimethyl)benzene (13)

$CDCl_3$, 23 ºC

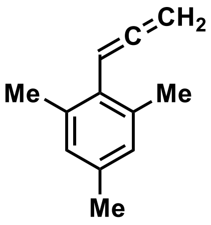

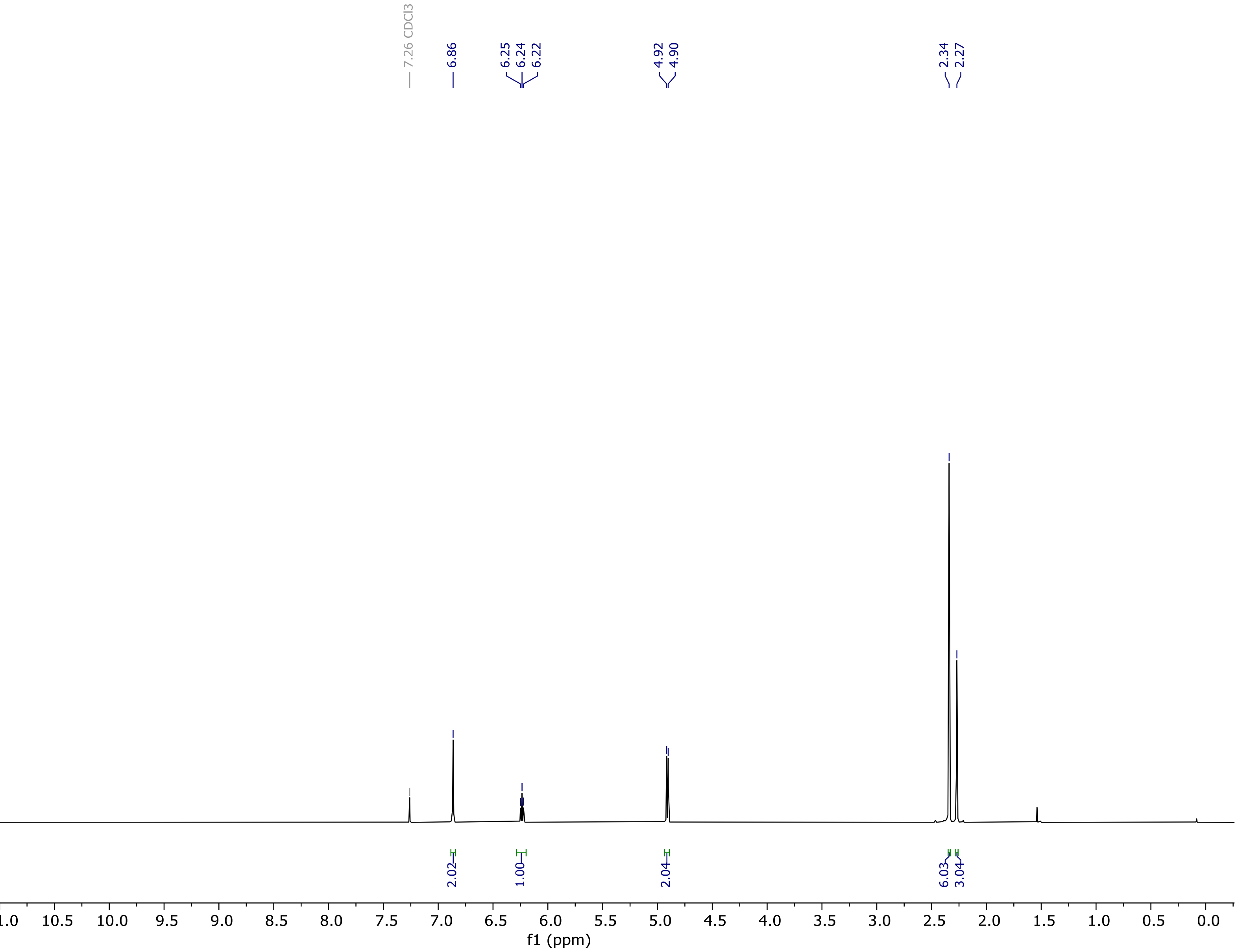

^{13}C NMR of allenyl-(2,4,6-trimethyl)benzene (13)

$CDCl_3$, 23 ºC

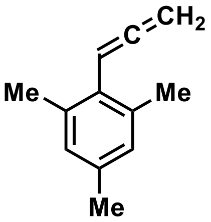

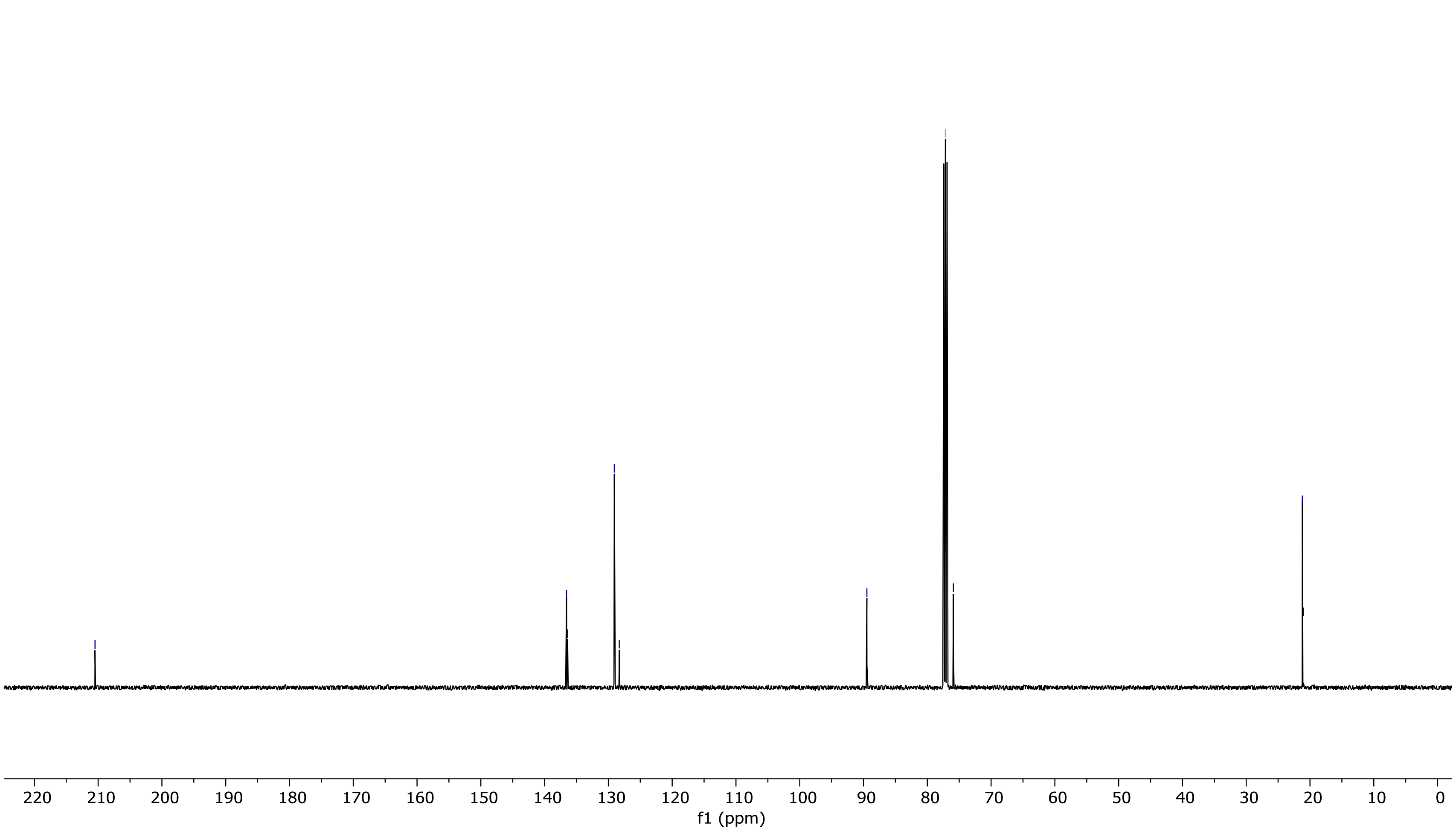

^{1}H NMR of allenyl-(4-*tert*-butyl)benzene (14)

$CDCl_3$, 23 ºC

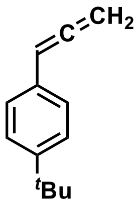

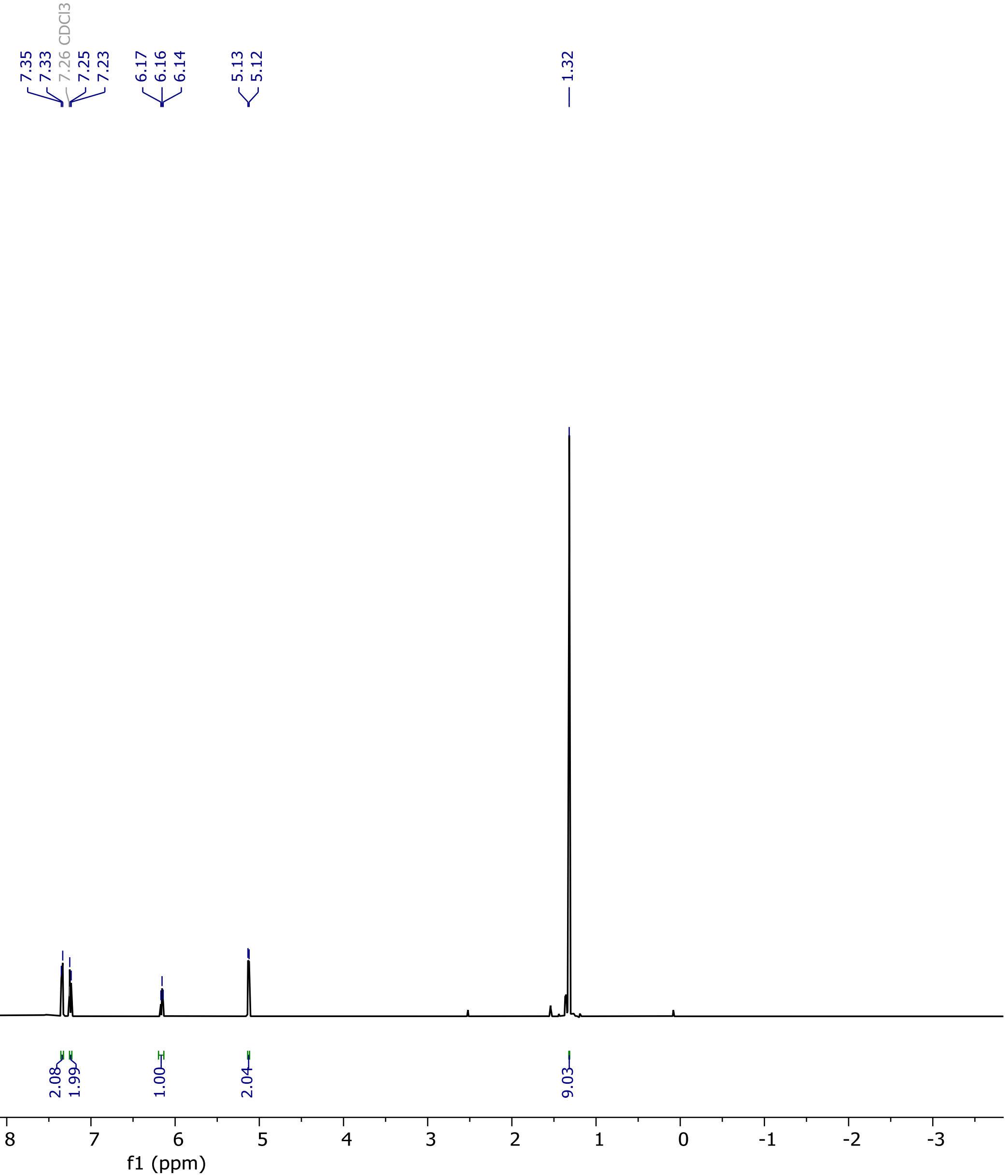

^{13}C NMR of allenyl-(4-*tert*-butyl)benzene (14)

CDCl$_3$, 23 ºC

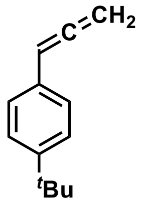

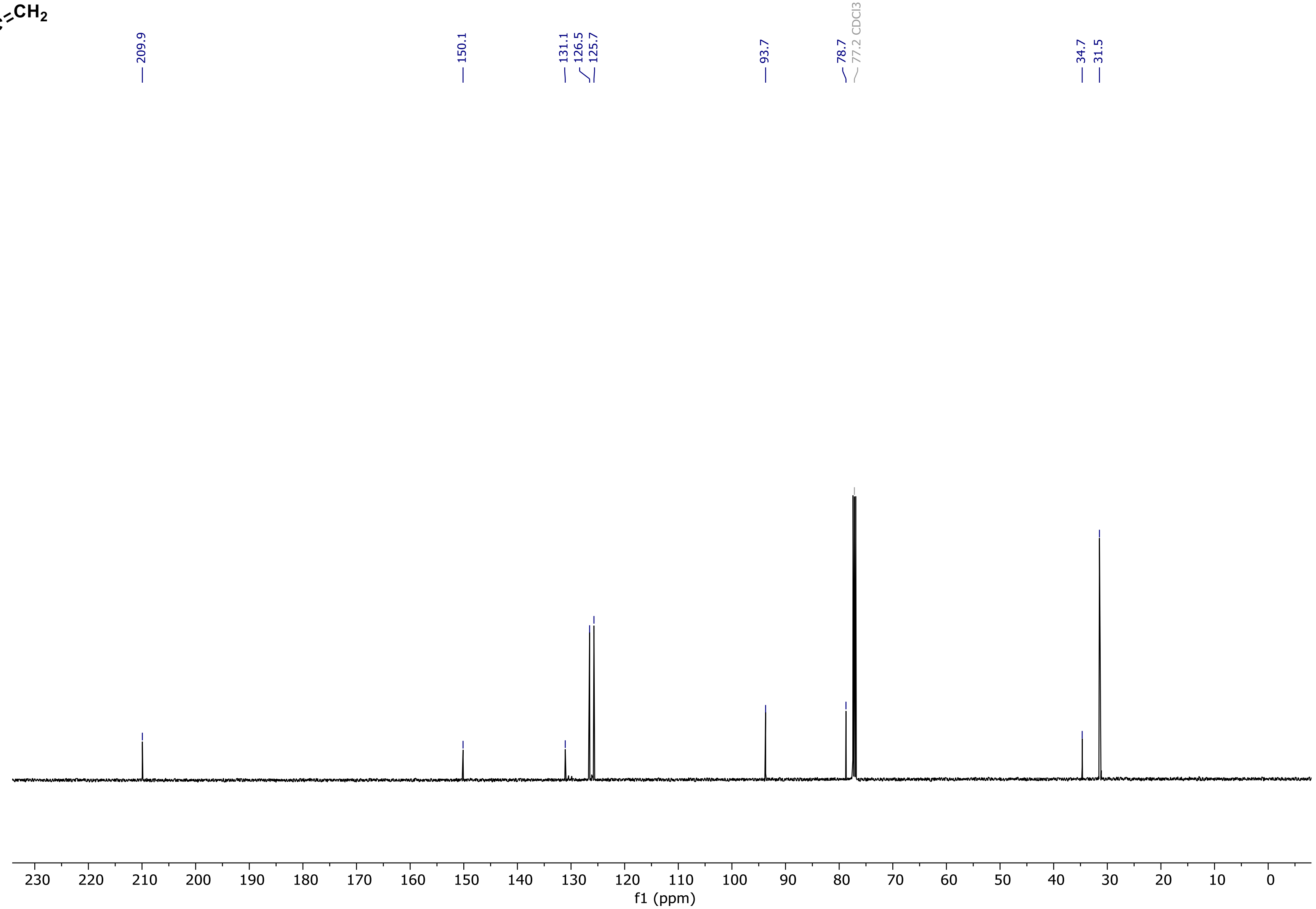

^{1}H NMR of 3-allenyl-1-boc-azetidine (15)

$CDCl_3$, 23 ºC

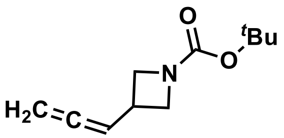

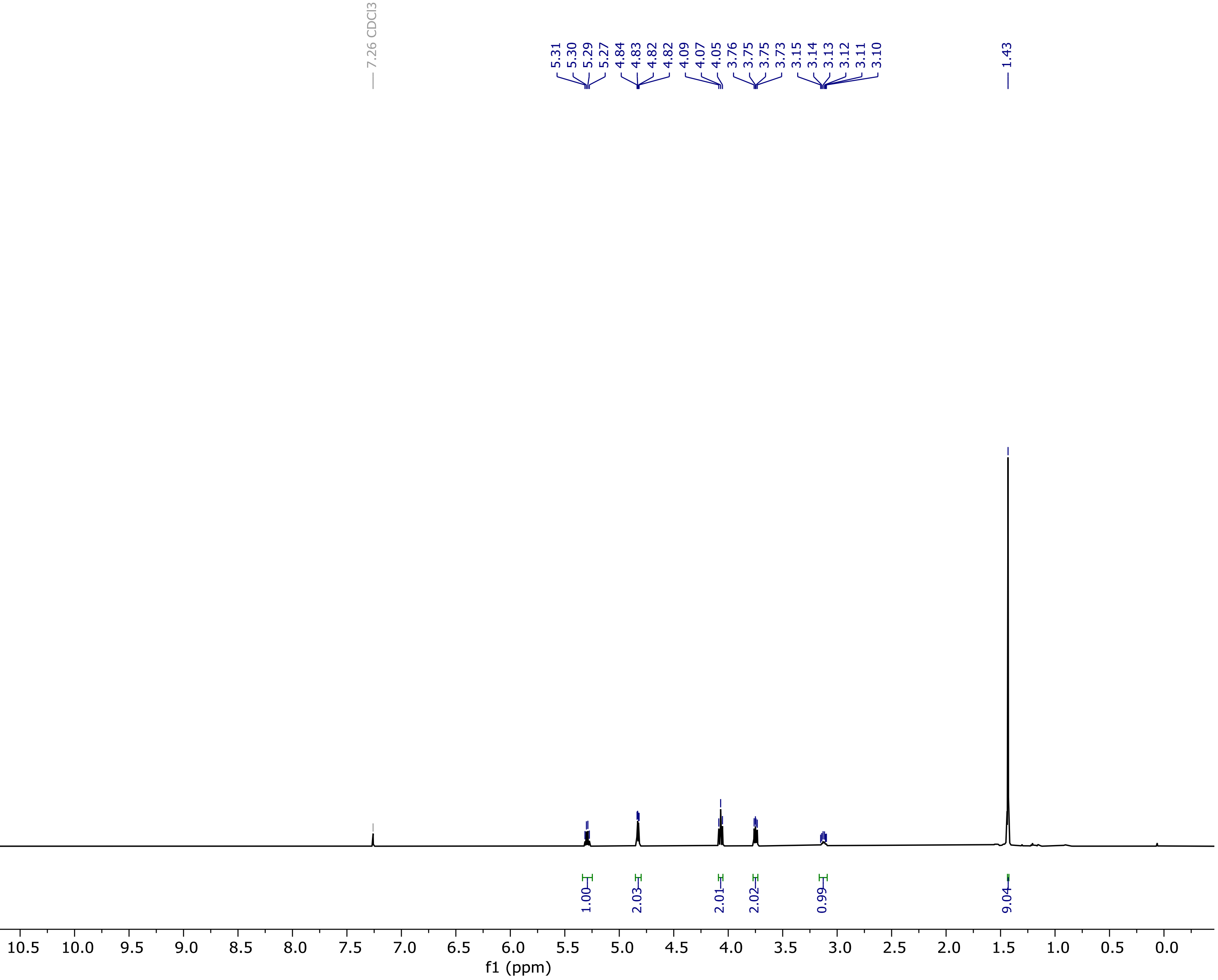

^{13}C NMR of 3-allenyl-1-boc-azetidine (15)

$CDCl_3$, 23 ºC

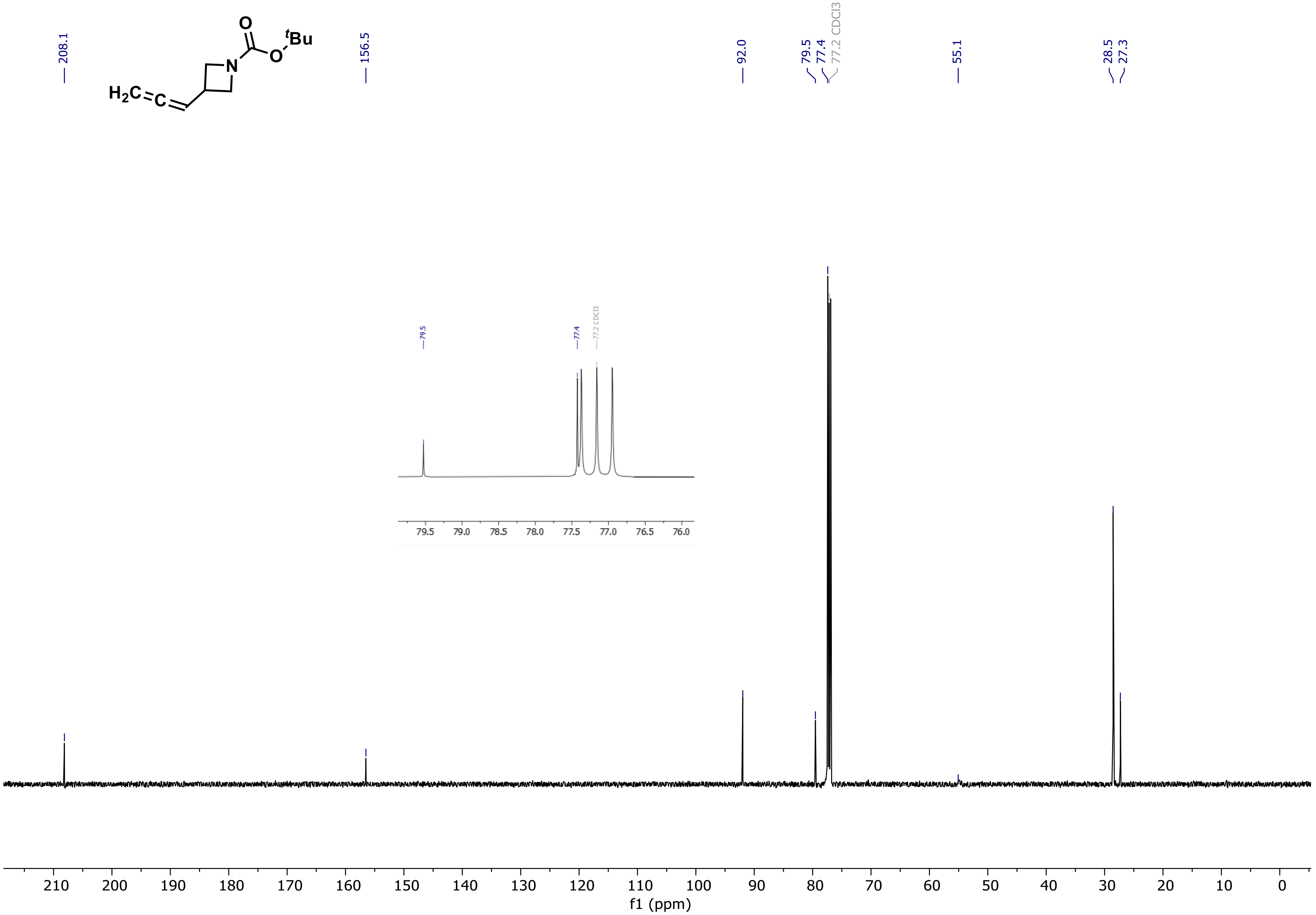

REFERENCES

1. Li, T.-S.; Li, J.-T.; Li, H.-Z. Modified and convenient preparation of silica impregnated with silver nitrate and its application to the separation of steroids and triterpenes. *J. Chromatogr. A* **1995**, *715*, 372-375.

2. Fulmer, G. R.; Miller, A. J.; Sherden, N. H.; Gottlieb, H. E.; Nudelman, A.; Stoltz, B. M.; Bercaw, J. E.; Goldberg, K. I. NMR chemical shifts of trace impurities: common laboratory solvents, organics, and gases in deuterated solvents relevant to the organometallic chemist. *Organometallics* **2010**, *29*, 2176-2179.

3. Roth, H. G.; Romero, N. A.; Nicewicz, D. A. Experimental and calculated electrochemical potentials of common organic molecules for applications to single-electron redox chemistry. *Synlett* **2016**, *27*, 714.

4. Tsai, H.; Roberts, J.D. Conformational equilibria of ephedrine and pseudoephedrine and hydrogen bonding. *Magn. Reson. Chem*., **1992**, *30*, 828-830.

5. Kashima, C.; Harada, K.; Omote, Y. The influence of a base on the methylation of aminoalcohols. *Can. J . Chem*. **1985**, *63*, 288.

6. Job, G. E.; Buchwald, S. L. Copper-catalyzed arylation of beta-amino alcohols. *Org Lett.* **2002**, *4*(21), 3703-3706.

7. Lansbergen, B.; Tewari, S.; Tomczyk, I.; Seemann, M.; Buchholz, H. L.; Rippegarten, M.; Cieminski, D. C.; Juliá, F.; Ritter T. Reductive cross-coupling of a vinyl thianthrenium salt and secondary alkyl iodides. *Angew. Chem. Int. Ed.* **2023**, *62*, e202313659.
